# Advances in Deep Learning Models for Ground Deformation Prediction Using InSAR Approach: A Systematic Review with Special Emphasis on Subsidence as a Geo-Environmental Hazard

**DOI:** 10.3390/s26196358

**Published:** 2026-10-08

**Authors:** Nitish Kumar, Mohammad Soyeb Alam

**Affiliations:** Department of Mining Engineering, Indian Institute of Technology (Indian School of Mines), Dhanbad 826004, India; 22dr0312@iitism.ac.in

**Keywords:** geo-environmental Hazard, ground deformation, subsidence, InSAR, deep learning models, prediction, hybrid neural networks, explainable AI (XAI), physics-informed neural network (PINN)

## Abstract

**Highlights:**

**What are the main findings?**

- Based on a systematic PRISMA (Preferred Reporting Items for Systematic Reviews and Meta-Analyses) review of 162 studies, a taxonomic study is performed for deep learning architectures for InSAR-based subsidence predictions, indicating that spatiotemporal hybrid models (Convolutional Neural Network–Long Short-Term Memory (CNN-LSTM), ConvLSTM, Transformer, and Graph Neural Network (GNN)) are consistently reported to outperform single-architecture baselines across the majority of reviewed studies, spanning mining, urban, and landslide applications.
- Through comparative performance analysis of 71 architectures, attention-augmented and hybrid decomposition-based frameworks are found to be the highest performing, with several architectures providing a coefficient of determination (R^2^) value above 0.97 and sub-cm accuracy.

**What are the implications of the main findings?**

- The paper addresses a major gap in the literature by focusing on the mining-induced subsidence problem, which was discussed in less than 5% of previous DL-based InSAR review papers. The presented taxonomy provides a framework for researchers developing deformation prediction models in mining contexts.
- A tangible seven-point research roadmap for the field, including explainable AI, physics-informed neural networks, GNN-Transformer integration, geospatial foundation models, and multi-source data fusion.

**Abstract:**

Ground deformation, especially subsidence, is a geo-environmental hazard that needs to be assessed and predicted in advance to mitigate its effects efficiently and in a timely manner. Interferometric Synthetic Aperture Radar (InSAR) provides high-resolution, all-weather ground deformation monitoring, while advances in Deep Learning (DL) have significantly improved its analytical capabilities. Despite growing interest in integrating DL with InSAR, existing systematic reviews have focused mainly on urban infrastructure, leaving mining-induced subsidence comparatively underexplored. This review addresses this gap by applying the PRISMA framework to systematically evaluate 162 peer-reviewed studies, including 21 core articles on DL-based mining subsidence and 58 supporting studies from related deformation applications. This paper examines DL architectures, including CNNs, LSTMs, GRUs, ConvLSTMs, Transformers, and Graph Neural Networks, and assesses their roles in data decomposition, spatiotemporal feature extraction, hyperparameter optimization, and multi-source data fusion. Comparative analysis of 71 models shows that attention-enhanced CNN-LSTM and ConvLSTM architectures consistently outperform single-model approaches. Key challenges include limited mining datasets, poor cross-site transferability, lack of standardized benchmarks, and insufficient geomechanical integration. Finally, we propose a seven-point research roadmap to support reliable, explainable, transferable, and operational early-warning systems for mining-induced subsidence monitoring.

## 1. Introduction

In recent years, Artificial Intelligence (AI) has made tremendous strides in predictive modeling across various fields of science and engineering. Deep Learning (DL), a subset of Machine Learning (ML) that relies on neural networks to automatically extract features that are relevant to a given dataset, has been at the forefront of AI’s recent revolution in predictive modeling. This is because Deep Learning can automatically learn the most relevant features of a given dataset through hierarchical representations [1,2,3]. Unlike traditional Machine Learning techniques that rely on support vector machines [4], random forests [5], and gradient boosting [6], which rely heavily on feature engineering as well as crafting input representations based on domain knowledge.

The architecture that forms the basis of Deep Learning can be traced to a network known as a Multilayer Perceptron (MLP), which relies on interconnected layers of artificial neurons to approximate any function [7]. However, it was in the 2000s that Deep Learning gained popularity, especially after the development of Deep Belief Networks (DBNs) [8]. These networks were efficient in training deep networks using a technique known as layer-wise pre-training [8,9].

Deep learning (DL) is one of the most influential technologies for artificial intelligence (AI) and has shown excellent performance in various applications, exhibiting its ability to handle large amounts of data and complex calculations [10,11,12]. This technology is found to have huge potential as a game changer for geotechnical modeling, especially for subsidence prediction, for which accurate prediction is very important for ensuring safety, sustainability, and disaster management [2,13]. The emergence of high-resolution remote sensing technology, especially Interferometric Synthetic Aperture Radar (InSAR) technology, has led to a paradigm shift from empirical and physical modeling to data-driven modeling, enabling complex spatiotemporal deformation modeling [14,15]. The conventional methods using probability integral and finite element analyses rely on certain assumptions, such as homogeneity and boundary conditions, and hence cannot guarantee accuracy for predicting subsidence, especially due to geological heterogeneity [16,17]. Conventional methods cannot predict complex phenomena, whereas deep learning models can capture nonlinear relationships in large amounts of data and make predictions in complex real-world settings [18].

In contrast, deep learning models have proven effective at discovering complex associations and underlying patterns in large-scale multivariate datasets without specific physical parameterizations [1]. Long Short-Term Memory (LSTM) models and their extensions have proven effective in time series analysis of earth deformation [19,20,21], which is effective in mitigating long- and short-term effects and gradient vanishing. The hybrid approach of using Small Baseline Subset Interferometric Synthetic Aperture Radar (SBAS-InSAR)-based displacement time series and LSTM models has proven more effective in comparison to traditional models in predicting mining-induced subsidence [22,23]. Moreover, hybrid models of Ensemble Kalman Filter (EnKF) optimized LSTMs have proven effective in improving the convergence rate and accuracy in monitoring airport runway subsidence [20]. Additionally, errors in predicting transmission tower foundations have been minimized using beetle swarm optimization (BSO) and backpropagation neural networks (BPNN) [24]. Besides, Convolutional Neural Networks (CNNs) are powerful tools for capturing spatial patterns in InSAR interferograms to detect subsidence and fault zones [14,25]. Recently, attention mechanisms and transformer models, such as the GeoTemporal Transformer (GTFormer), have further improved regional subsidence prediction by leveraging self-attention [26,27].

Several methods have emerged, in line with the development of architectures that improve stability, interpretability, and generalizability. Techniques such as Variational Mode Decomposition (VMD) [28], Singular Spectra Analysis [29], and wavelet decomposition (WD) [30] are employed for subsidence data decomposition into trend and fluctuation components. These techniques are initially premised on their ability to separate noise and facilitate the extraction of temporal patterns [31].

The multi-scale space-time frameworks like Multiscale Geographically and Temporally Weighted Regression-Convolutional Neural Network-Bidirectional Long Short-Term Memory-Attention Mechanism (MGTWR-CNN-BiLSTM-AM) [32] describe spatial non-uniform deformation and scale-dependent deformation in subsidence data. These techniques utilize weighted regression, convolutional feature extraction, and attention mechanisms. Techniques such as Dual-Attention-Mechanism Convolutional Long Short-Term Memory Network (DACLnet) [33] apply dual-attention mechanisms to improve prediction and assign relative importance to temporal and spatial features. Techniques such as Maximum Information Coefficient (MIC) analysis facilitate the extraction of information from auxiliary variables. Ensemble and transfer learning further improve robustness and generalizability in subsidence prediction [34,35,36,37]. These trends align with the development of multimodal and spatiotemporal deep learning models. These models incorporate InSAR data and auxiliary variables, such as groundwater levels, soil, and meteorological data, to further improve generalizability [21]. With growing interest in DL-InSAR integration, existing systematic reviews primarily focus on urban infrastructure monitoring [38], covering only 4% of mining subsidence cases-underscoring a critical gap that this review addresses.

This research paper provides an in-depth review of various models and methodologies of deep learning in detecting and predicting geospatial deformations, which include ground deformation, mining subsidence, landslide/slope instability, and other areas of geoscience and engineering. A synthesis of the available literature from the mining and other relevant geospatial fields is presented to provide effective modeling strategies and methodological trends. The research focuses on various deep learning-based architectures such as convolutional neural networks (CNN), recurrent neural networks (RNN), long short-term memory (LSTM), gated recurrent units (GRU), temporal convolutional networks (TCN), and transformer-based architectures, as well as their advanced forms such as Bidirectional Long Short-Term Memory (BiLSTM), Hybrid Convolutional Neural Network-Long Short-Term Memory (hybrid CNN-LSTM) architectures, and attention-based deep learning architectures. In addition, the review also includes the relevant methodologies, which are temporal decomposition methods (e.g., variational mode decomposition, singular spectrum analysis, and wavelet transforms), meta-heuristic optimization techniques, spatiotemporal modeling methods, feature engineering techniques (e.g., maximum information coefficient analysis), and ensemble-based methods. Moreover, Data Acquisition and Monitoring Technologies, such as SBAS-InSAR, Persistent Scatterer Interferometric Synthetic Aperture Radar (PS-InSAR), Distributed Scatterer Interferometric Synthetic Aperture Radar (DS-InSAR), Global Navigation Satellite System (GNSS), and atmospheric correction methods, are also discussed. Furthermore, various comparative performance evaluation techniques are also analyzed. The main aim of this research is to identify various best practices and gaps in existing research to provide a solid foundation for designing efficient deep learning-based frameworks for predicting subsidence.

## 2. Method of Review Process

**Figure 1:**
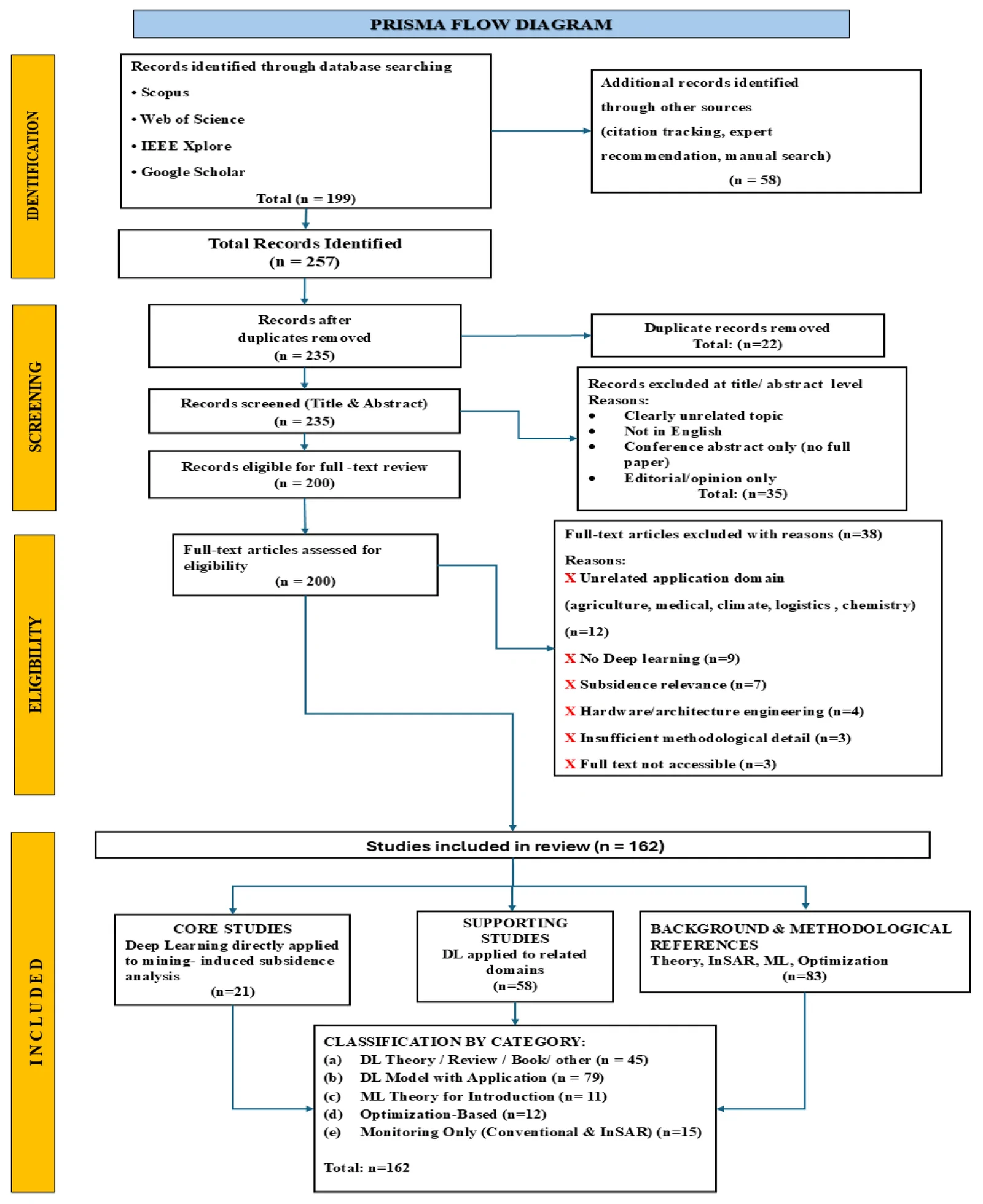
PRISMA flow diagram illustrating the systematic process of record selection and the stages involved in identifying the final set of relevant studies.

This review was conducted in accordance with the Preferred Reporting Items for Systematic Reviews and Meta-Analyses (PRISMA) 2020 guidelines. The completed PRISMA 2020 checklist is provided as Supplementary Material, and the study identification, screening, and inclusion process is summarized in the PRISMA flow diagram (Figure 1). Two independent reviewers performed the title and abstract screening, followed by full-text eligibility assessment according to the predefined inclusion and exclusion criteria. Any disagreements during the screening and study selection process were resolved through discussion until consensus was reached. This review was not registered in a public registry (e.g., PROSPERO), and no separate review protocol was prepared prior to conducting the review.

### 2.1. Research Objective and Scope

The objective of the review is to perform a systematic analysis of the available literature on deep learning-based methods for subsidence/deformation prediction using InSAR time-series data. The research questions considered for conducting this systematic review are as follows:

RQ1: What have been the developments and evolutions of deep learning architectures for InSAR-based surface deformation prediction?

RQ2: How are InSAR time-series data pre-processed, decomposed, and integrated with deep learning models for deformation forecasting?

RQ3: What optimization strategies and hybrid modeling frameworks have been proposed to enhance prediction accuracy?

RQ4: What are the prevailing challenges, limitations, and future research directions in this domain?

### 2.2. Search Strategy

To conduct the systematic review, a comprehensive literature search was conducted using several databases, including Scopus, Web of Science, IEEE Xplore, Google Scholar, ScienceDirect, and SpringerLink. The literature search used Boolean logic-based keywords and phrases as follows:

Group 1 (Remote Sensing): “InSAR,” “Interferometric Synthetic Aperture Radar,” “SBAS-InSAR,” “PS-InSAR,” “DInSAR,” “MT-InSAR,” “SAR Interferometry”.

Group 2 (Deformation and its factors): “surface deformation,” “land subsidence,” “ground subsidence,” “mining subsidence,” “surface settlement,” “ground displacement,” “deformation prediction,” “deformation monitoring”.

Group 3 (Deep Learning): “deep learning,” “neural network,” “LSTM,” “Long Short-Term Memory,” “CNN,” “Convolutional Neural Network,” “ConvLSTM,” “Transformer,” “BiLSTM,” “recurrent neural network,” “attention mechanism,” “GRU,” “time series prediction,” “deep neural network”.

To formulate the search query, terms from these three groups were combined using the AND operator, and terms within groups were combined using the OR operator. The search for research articles was conducted iteratively from January 2024 to July 2025, and from time to time updates were made to include the latest research articles. Also, research papers on DL for other forms of land subsidence, such as groundwater-induced and urbanization-induced, that have methodological relevance to mining-related land subsidence, as well as fundamental papers on DL architectures and DL-related papers from geoscience-related fields, were included.

### 2.3. Inclusion and Exclusion Criteria

To ensure the relevance and quality of the selected literature, the following inclusion and exclusion criteria were applied:

Inclusion criteria:

- Peer-reviewed journal articles, conference papers from tier-1 conferences, book chapters, and foundational literature from previous years are included for theoretical purposes.
- Journal articles applying DL models for subsidence prediction or deformation-related studies.
- Journal articles presenting new DL models for spatiotemporal geoscience predictions.
- Journal articles presenting new DL models for the prediction of subsidence, although not applied previously.
- Journal articles from Q1 or Q2 journals (Journal Citation Reports or Scimago Journal Ranking).
- Comparative studies involving traditional methods, baseline machine learning models, or alternative deep learning architectures.
- Journal articles written in English.

Exclusion criteria:

- Papers without peer review from journals (non-peer-reviewed preprints without publication).
- Research papers not using deep learning models for predictions (although some of the ML models are included for the Section 1).
- Papers from other fields such as agriculture, medicine, logistics, chemistry, ecology or climate classification that have no direct relationship to geospatial deformation prediction.
- Research papers that fail to provide any quantitative analysis of the prediction performance.
- Publications of the same study appearing more than once.
- Research papers for which full-text access cannot be obtained due to lack of institutional access

#### Classification of Core and Supporting Studies

Of the 162 included studies, 21 are classified as core studies: papers that directly apply deep learning to InSAR-derived, mining-induced subsidence prediction or detection. The remaining 141 studies (of which 58 are drawn from closely related deformation domains) are classified as supporting studies: deep learning or InSAR studies from other deformation contexts (urban groundwater-related subsidence, landslide and slope deformation, infrastructure and heritage monitoring) or foundational deep learning architecture papers, retained where they contribute methodological approaches that are transferable to mining-induced subsidence. This classification was applied during data extraction (Section 2.5): core studies are consolidated in the primary core studies summary table, while supporting studies are distributed across subsequent domain-specific tables (non-mining subsidence, landslide/slope deformation, and other geoscience/engineering deformation, respectively).

### 2.4. Study Selection Process

Study selection and data extraction were carried out by the lead reviewer and cross-checked by a second reviewer at both the title/abstract and full-text stages; disagreements on inclusion or extracted values were resolved through discussion, with a third party consulted where consensus could not be reached.

The study selection process is depicted in Figure 1. The search results provided a pool of approximately 257 articles. After removing duplicates, two rounds of filtering were conducted:

i. Title and Abstract Screening: The first screening of research articles was done through the title and abstract to see if they belong to the scope of InSAR-based deformation prediction using deep learning techniques. Those studies not meeting the above criterion were automatically rejected.
ii. Full-Text Assessment: In this round, the remaining research articles were assessed based on their full texts regarding inclusion criteria and the scientific novelty of the approach.

Through this process, 162 articles were finally chosen for the study. These articles were research papers on predicting deformation using deep learning with InSAR. They were augmented with more theories from different sources related to deep learning, InSAR, optimization, and other fields of study. These theories served as background information for the study.

### 2.5. Data Extraction and Categorization

The information for each selected article is as follows:

- Bibliographic information: The author list, publication year, publication title, and publication type.
- InSAR technique: The InSAR technique used by the author. The InSAR technique used is either SBAS-InSAR, PS-InSAR, MT-InSAR, or a combination.
- Deep learning technique: The deep learning technique used by the author. The technique used is either CNN, LSTM, BiLSTM, GRU, ConvLSTM, or a combination.
- Data decomposition and pre-processing techniques: Variational Mode Decomposition (VMD), Wavelet Transform, Empirical Mode Decomposition, etc.
- Optimization techniques: Particle Swarm Optimization (PSO), Sparrow Search Algorithm, Bayesian Optimization (BO), etc.
- Application domain: Mining subsidence, land subsidence in cities, infrastructure monitoring, landslide displacement, permafrost, etc.
- Study area and geographic scope.
- Performance evaluation criteria used: Root Mean Square Error (RMSE), Mean Absolute Error (MAE), Coefficient of Determination (R^2^), etc.
- Findings of the research, limitations reported in the research, and gaps in the research.

### 2.6. Synthesis and Analysis Framework

**Figure 2:**
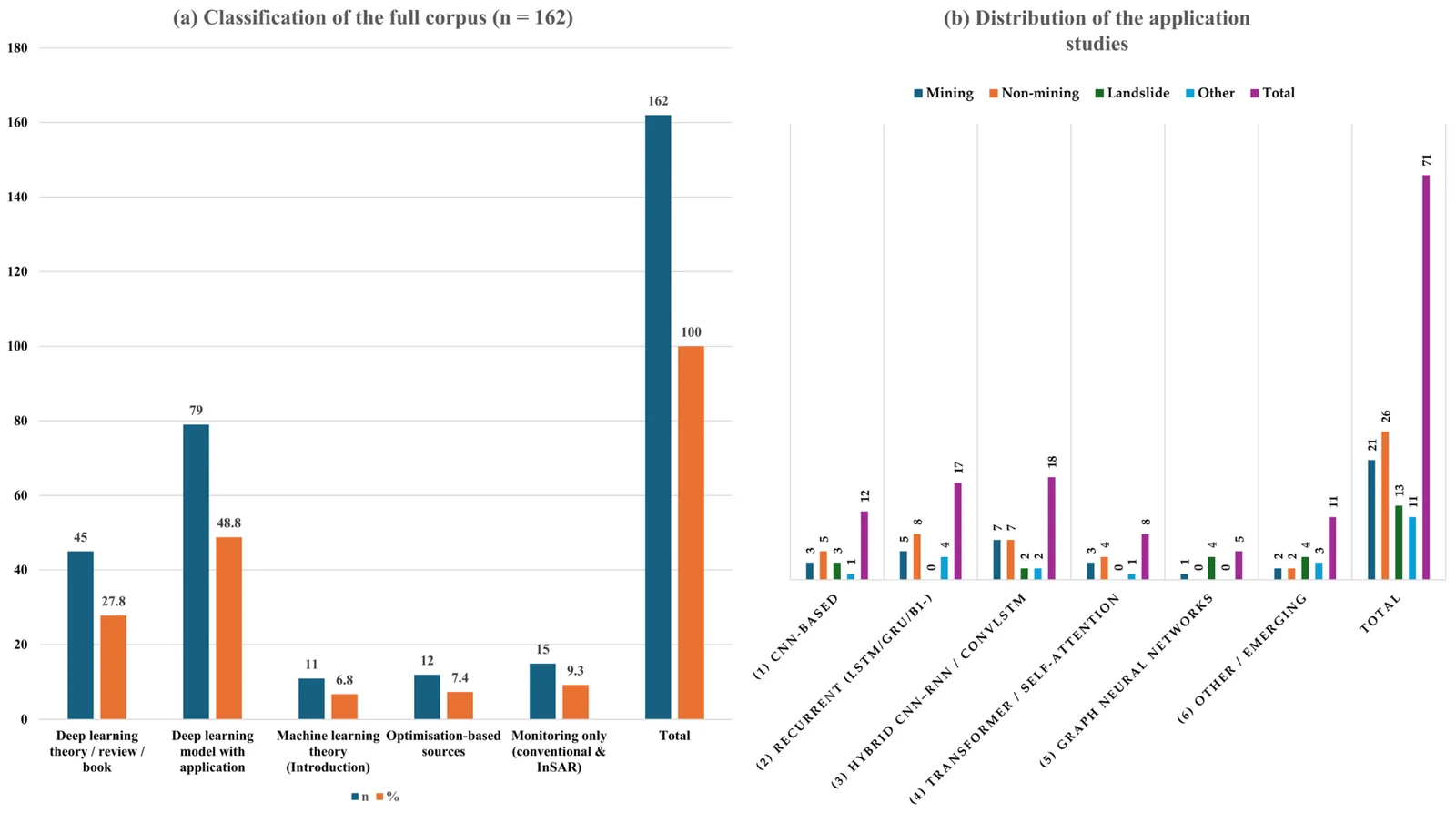
Composition of the reviewed literature. (**a**) Classification of the full corpus (n = 162) by study category, consistent with the PRISMA flow diagram in Figure 1. (**b**) Distribution of the application studies with quantitative prediction results (n = 71; Section 2.5) by primary deep learning architecture and application domain.

The extracted information was synthesized using a thematic and comparative analysis framework. The reviewed research articles were grouped into specific themes based on the primary deep learning architecture used in the research (Figure 2):

- Convolutional Neural Network (CNN)-informed deep learning architecture-based research
- Recurrent Neural Network (RNN) and Long Short-Term Memory (LSTM)-informed deep learning architecture-based research
- Hybrid CNN and LSTM/Convolutional LSTM-based research
- Deep learning architecture informed by the transformer and attention mechanism
- Optimization technique-enhanced deep learning architecture
- Ensemble and other emerging trends in deep learning architecture-based research

The research articles were compared according to the architectural innovations used in the research, the prediction accuracy of the proposed deep learning architecture-based models, and the application domains of the proposed models. Comparative tables and figures were included to show the trends in the deep learning architecture used in the research articles. A critical analysis of the research was carried out based on the findings, limitations, and gaps reported in the research articles. This ensures a comprehensive and transparent assessment of the current trends in deep learning architecture-based research in the field of InSAR-based surface deformation prediction.

**Figure 3:**
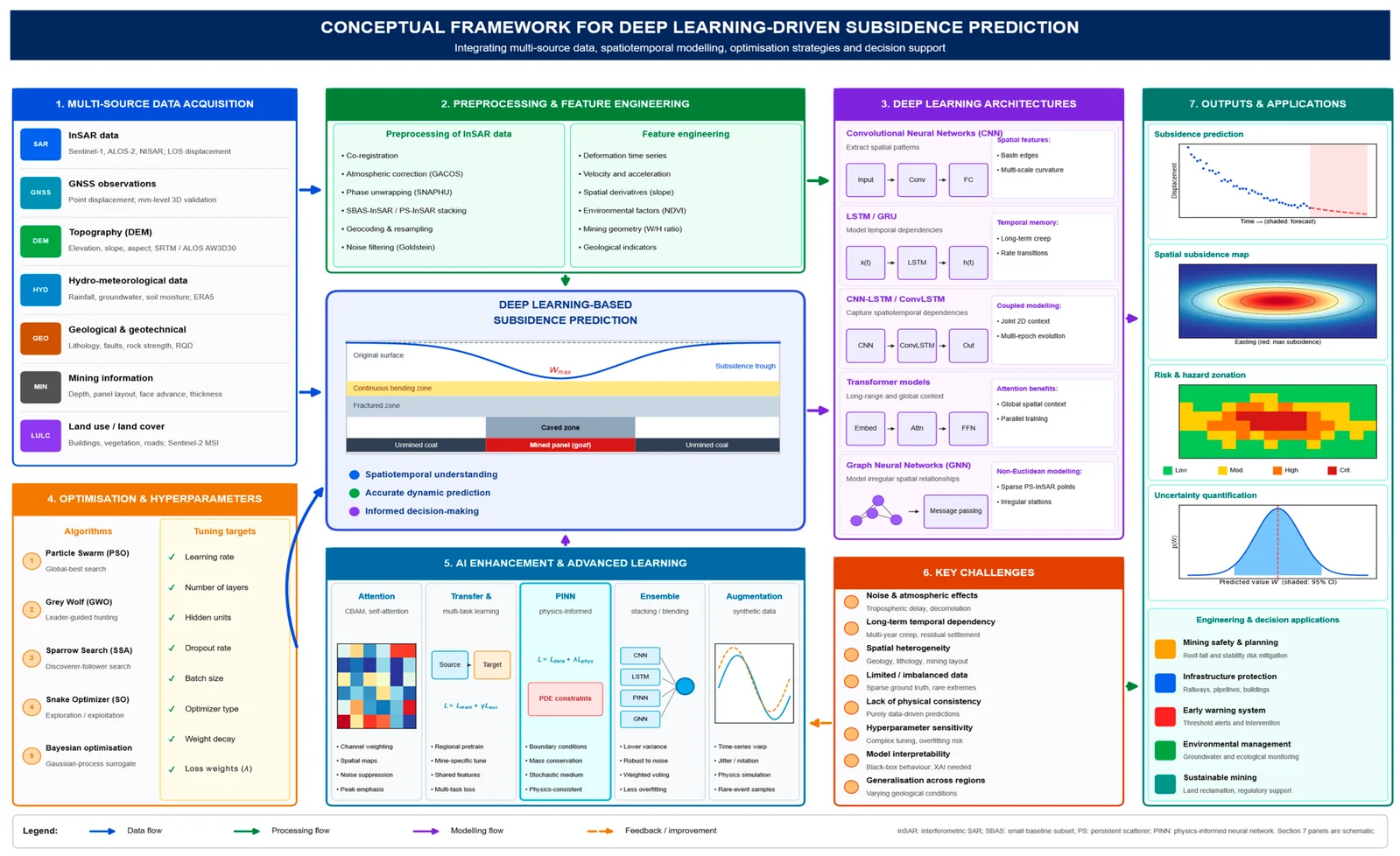
Conceptual framework of Deep Learning-based Subsidence prediction that combines geospatial datasets, preprocessing and feature extraction, spatiotemporal learning models, optimization approaches, uncertainty evaluation, and practical decision-support applications.

Based on the analysis and synthesis of the reviewed literature, a comprehensive conceptual framework for subsidence prediction is presented in Figure 3. This framework integrates multi-source geospatial data, preprocessing and feature engineering methods, deep learning-based spatiotemporal architectures, optimization techniques, uncertainty analysis, and decision-support applications.

## 3. Data Acquisition and Monitoring Technologies

To describe the prediction methods, it is necessary to know the structure of the data on which deep learning is applied. The combination of Remote Sensing and ground-based monitoring is a key point in the prediction of subsidence [39,40]. Synthetic Aperture Radar (SAR) is an active microwave remote-sensing system, whereas Differential Interferometric Synthetic Aperture Radar (DInSAR) estimates surface deformation from the phase difference between SAR acquisitions. SBAS-InSAR is a key technique in the acquisition of spatiotemporal subsidence data. SBAS-InSAR is a technique that provides time-series deformation data with high spatial resolution over large areas [41,42]. This technique has been successfully applied in mining areas with monitored subsidence rates. MT-InSAR (Multi-Temporal Interferometric Synthetic Aperture Radar) is used to improve the coverage of the PS-InSAR and DS-InSAR techniques [43,44,45]. The combination of the atmospheric correction technique using GACOS (Generic Atmospheric Correction Online Service) has improved the quality of the acquired data by correcting the effects of phase delay in SAR interferometry [14,46]. Recently, Bayesian-based techniques for phase optimization have proven their effectiveness in terms of accurate determination of deformation parameters [47]. Also, the Global Navigation Satellite System (GNSS) is another technique that has provided a source of ground truth in the acquisition of subsidence data [48,49,50,51]. These techniques have provided robust data sets from which deep learning models can be trained and validated.

## 4. Advances in Deep Learning Models Relevant to Subsidence Prediction

This Section discusses the architectural theory of different deep learning models. In this section of the article, the evolution of neural networks is briefly discussed with a focus on how these neural networks have been developed from traditional to specialized deep learning models capable of dealing with the spatiotemporal complexities of land subsidence or surface deformation. These developments show how deep learning models have moved beyond simple pattern recognition to process complex satellite data.

The subsections below follow the six architecture classes defined in Section 2.6 and Figure 2b: CNN-based models (Section 4.1), recurrent models (Section 4.2), hybrid CNN–RNN and ConvLSTM models (Section 4.3), Transformer and self-attention-centric models (Section 4.4 and Section 4.6), graph neural networks (Section 4.7) and other emerging architectures (Section 5.2 and Section 5.6).

To avoid duplicating content between this section and Section 5, Chapter 4 is restricted to the general architectural theory and mathematical formulation of each model family as originally proposed in the literature. Section 5 revisits these families only to the extent that their formulation is adapted, combined, or applied specifically to subsidence prediction; general mechanism descriptions are not repeated there, and readers seeking the base formulation of a given architecture (e.g., ConvLSTM, CNN-LSTM, attention, Transformer) should refer to the corresponding subsection here.

### 4.1. Convolutional Neural Networks for Spatial Feature Extraction

Convolutional Neural Networks (CNNs) have proven to be the basic deep learning structure utilized for spatial feature extraction in the context of raster geospatial data, especially those from the InSAR deformation map and the Digital Elevation Model [52,53,54]. The CNN algorithm involves several convolutional layers, whereby learnable filters are applied to the input features, resulting in the automatic detection of image hierarchy without the need for human-defined features [14,55]. This capability proves invaluable in subsidence analysis, where deformation patterns exhibit complex spatial structures at multiple scales.

Generally, the CNN architecture in subsidence applications includes several convolutional layers along with activation functions, usually ReLU (Rectified Linear Unit) functions and pooling processes for dimensional reduction [56,57]. Convolutional layers can extract low-level information from image inputs, such as edges and gradients, whereas deeper layers are capable of capturing high-level information like the morphology of subsidence basins, slope information, and spatial correlation with geological features [58,59,60,61]. Pooling retains useful spatial features, thereby making computation more efficient and allowing the analysis of large-scale regional subsidence data sets [62,63].

In mining subsidence applications, the utilization of CNNs has proven to be especially efficient in detecting subsidence troughs in SAR interferograms, achieving automated detection accuracy exceeding human observers’ ability and enabling fully unsupervised operational deployment [54]. This has been achieved through the incorporation of CNN alongside other neural network architectures for subsidence analysis, as illustrated by the CNN-LSTM architecture in [14].

**Figure 4:**
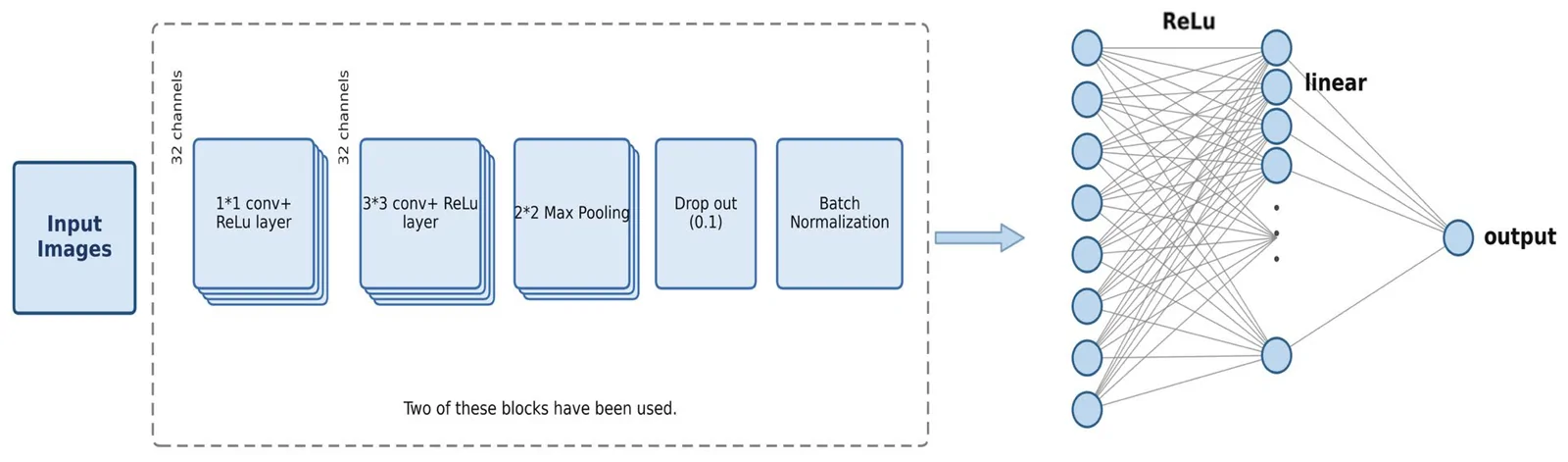
Conceptual illustration of a Convolutional Neural Network (CNN) architecture for image classification, redrawn based on the general CNN design principles described in Azarm et al. [52].

Recent developments in CNN architecture have enabled several variations, such as dilated convolutional networks, group convolution networks, and depth-wise separable convolutions. The benefit of using these variations is that deeper CNNs can be built, allowing for better feature extraction in an efficient manner [64,65,66]. These architectural innovations enable deeper networks with larger receptive fields, enabling the capture of broader spatial contexts relevant to subsidence processes that operate across multiple scales, from local failure mechanisms to regional basin-scale effects. A diagram of the CNN model is presented in Figure 4 [52].

### 4.2. Recurrent Cell Architectures

#### 4.2.1. Recurrent Neural Networks (RNNs)

**Figure 5:**
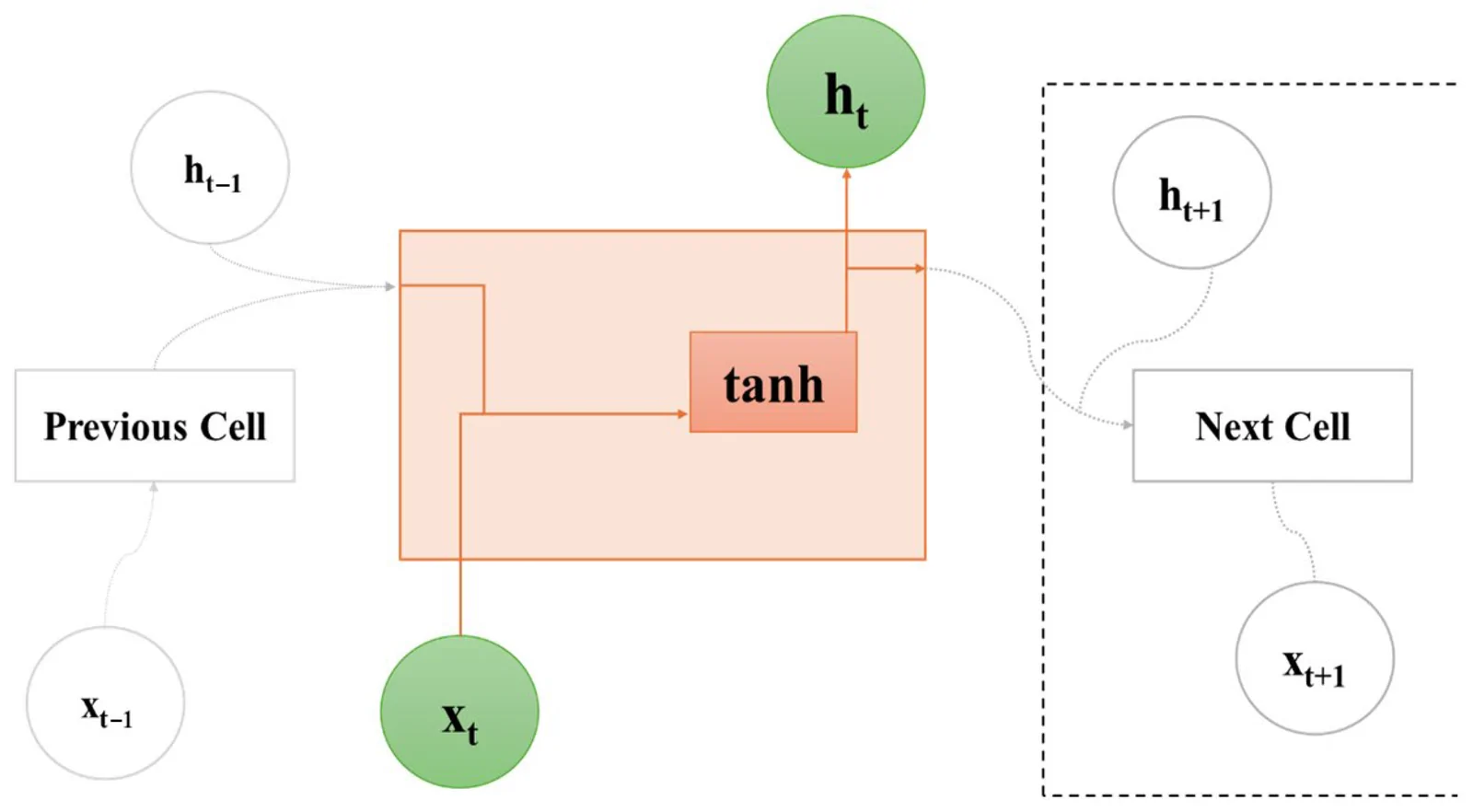
Original schematic illustrating the temporal state transition and nonlinear activation (tanh) within a standard RNN cell, based on the concept described by Waqas and Humphries [73].

To comprehensively address the limitations of convolutional networks in modeling persistent, long-term chronological relationships, **Recurrent Neural Networks (RNNs)** [67] and their advanced architectural variants, Long Short-Term Memory (LSTM) networks and Gated Recurrent Units (GRU), are deployed [68]. ANNs with recurrent connections are called recurrent neural networks (RNNs), which are capable of modeling sequential data for sequence recognition and prediction [69,70]. RNNs are made of high-dimensional hidden states that model temporal dependencies in time-series and sequential data using recurrent connections [71]. The basic structure of an RNN cell is shown in Figure 5, where the hidden state works as memory conditioned on its previous state [72].

Waqas and Humphries [73] identify a critical weakness that RNNs face: the issue of vanishing gradients, which impedes their ability to model long-term dependencies. To address this, variants like LSTMs and GRUs have been developed with “memory cells and gating mechanisms” that improve performance [74,75]. While RNNs performed well in modeling short-term sequences, they faced difficulties with long-term dependencies as a result of the vanishing gradient problem. Long Short-Term Memory (LSTM) networks have been developed to overcome this problem [76].

#### 4.2.2. The Long Short-Term Memory (LSTM)

**Figure 6:**
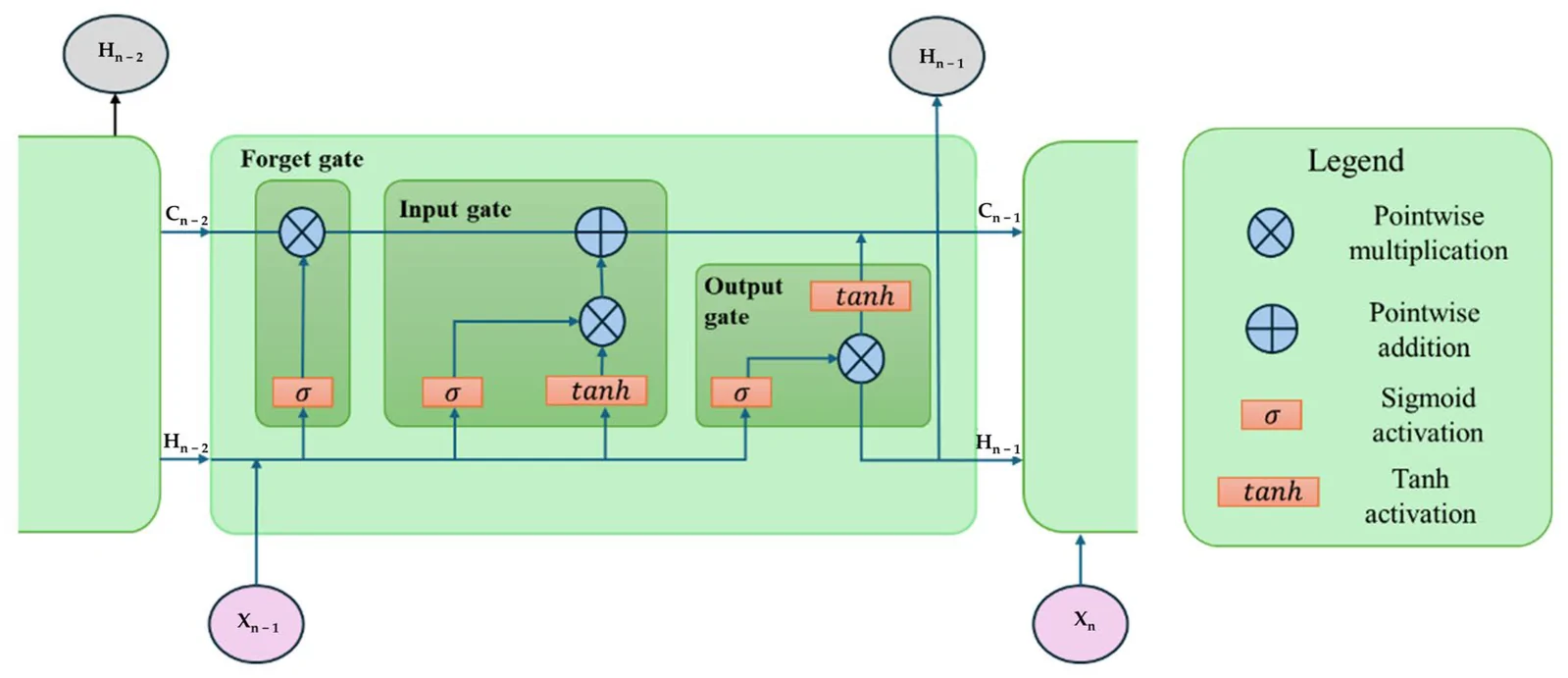
Conceptual schematic of the standard LSTM architecture (input/forget/output gates and cell state), illustrated based on the description in Ma et al. [77].

The Long Short-Term Memory networks mark an architectural advance through the development of gating units to regulate the flow of information into the network, its retention or forgetting over time [19]. An LSTM network architecture has been devised such that three types of gates work (input gate, forget gate, and output gate) along with a memory cell to manage information flow in the network [76]. The complete LSTM architecture with all gates is illustrated in Figure 6. The architecture of the LSTM directly addresses the problem of vanishing gradients through the creation of gradient highways from the memory cell to other parts of the network [75].

Regarding the problem of predicting the development of underground mines, LSTM networks are one of the most commonly used models within deep learning techniques, being especially useful for predicting future deformations based on previous deformation series obtained from InSAR [77,78]. An important strength that distinguishes LSTM networks from traditional models like ARIMA (Autoregressive Integrated Moving Average) and exponential smoothing is their ability to automatically extract temporal features without any prior feature engineering, an advantage that can be especially valuable in scenarios where there are no clear stationary or linear properties [79]. The combination of SBAS-InSAR displacement measures with LSTM prediction models has led to significant gains in terms of predictive accuracy, since the models were able to predict both the trends and rapid accelerations of deformations [22].

In recent times, more sophisticated LSTM-based methods have been introduced with the aim of improving prediction accuracy in addition to solving problems associated with subsidence prediction. In Bidirectional LSTM (BiLSTM), input data can be scanned and processed in the forward and backward directions. Therefore, every prediction made at a particular time will consider data from the past and the future, a feature that is extremely helpful when performing post-event analysis of deformations and filling in any missing data [80]. Through bidirectional processing, BiLSTM networks are better able to model subsidence patterns affected by the mining history as well as the future [81].

The inclusion of LSTM networks within optimization techniques has made them even more accurate at forecasting deformations, whereby meta-heuristic strategies have been used to explore hyperparameter spaces and find the optimal neural network architecture [74]. For instance, the Particle Swarm Optimization algorithm (PSO), Sparrow Search Algorithm, and Bayesian Optimization (BO) are some of the meta-heuristics that have been effectively incorporated into LSTM networks for tuning parameters such as learning rates, the size of hidden layers, and dropout rates among others [20,82].

#### 4.2.3. Gated Recurrent Units (GRU)

**Figure 7:**
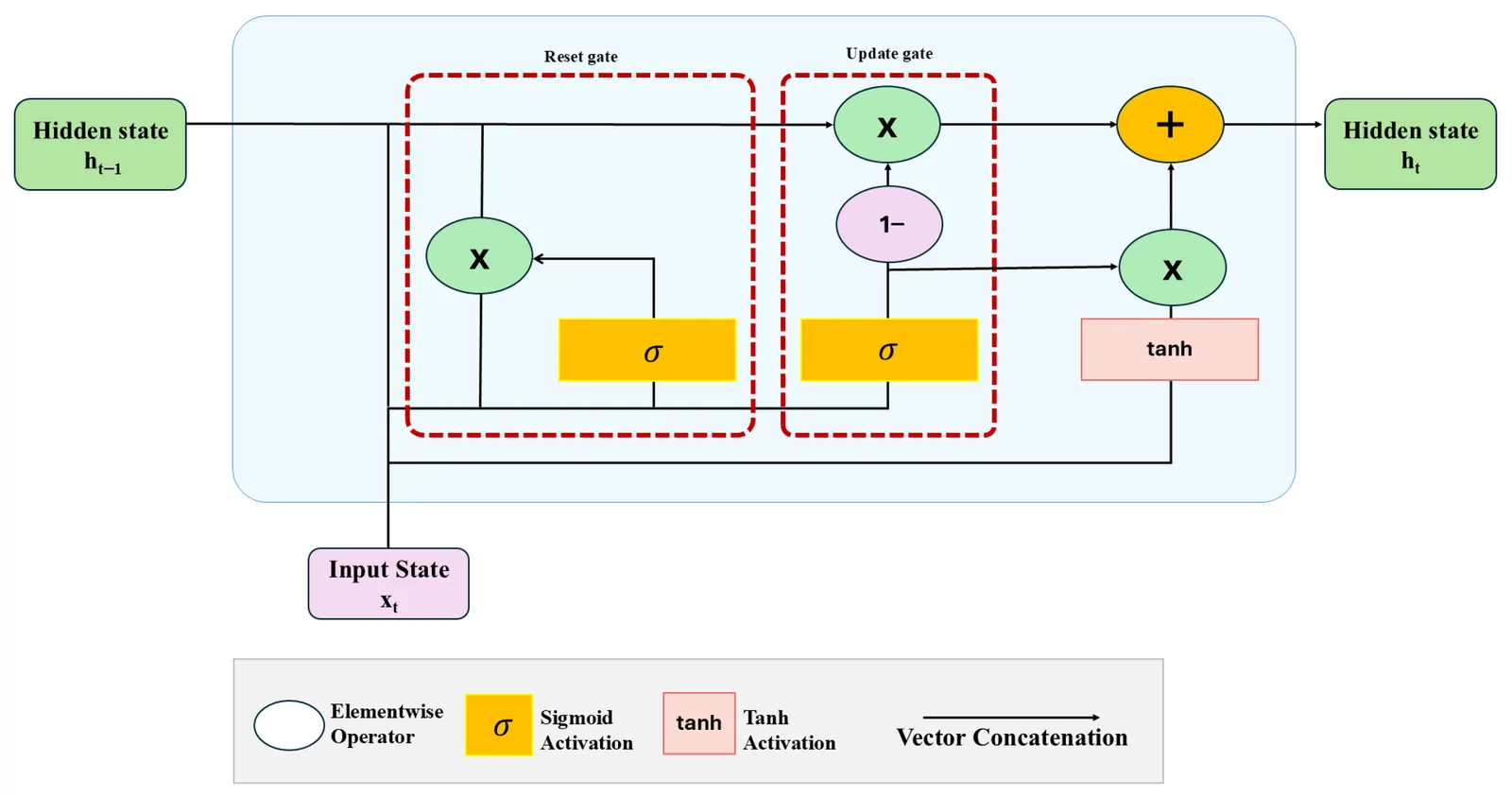
Original schematic of the Gated Recurrent Unit (GRU) update/reset gate mechanism, illustrated based on the concept described in Waqas and Humphries [73].

The Gated Recurrent Unit (GRU) is a streamlined variant of the LSTM that merges the cell state and hidden state and combines the forget and input gates into a single update gate. GRU’s update gate and reset gate mechanisms are shown in Figure 7, making it computationally efficient compared to LSTM. Additionally, GRU has fewer parameters and a less complicated structure compared to LSTM models [23,83,84].

- Efficiency: GRUs have fewer parameters than LSTMs, making them faster to train and less prone to overfitting on smaller datasets.
- Snake Optimization (SO-GRU): Xiao et al. [68] demonstrated the power of GRUs when combined with advanced optimization strategies. They used Snake Optimization (SO), a biomimetic algorithm, to fine-tune the GRU’s hyperparameters (learning rate, hidden units), achieving <8 mm error in 99.1% of samples.

#### 4.2.4. Bidirectional RNNs (BiLSTM/BiGRU)

**Figure 8:**
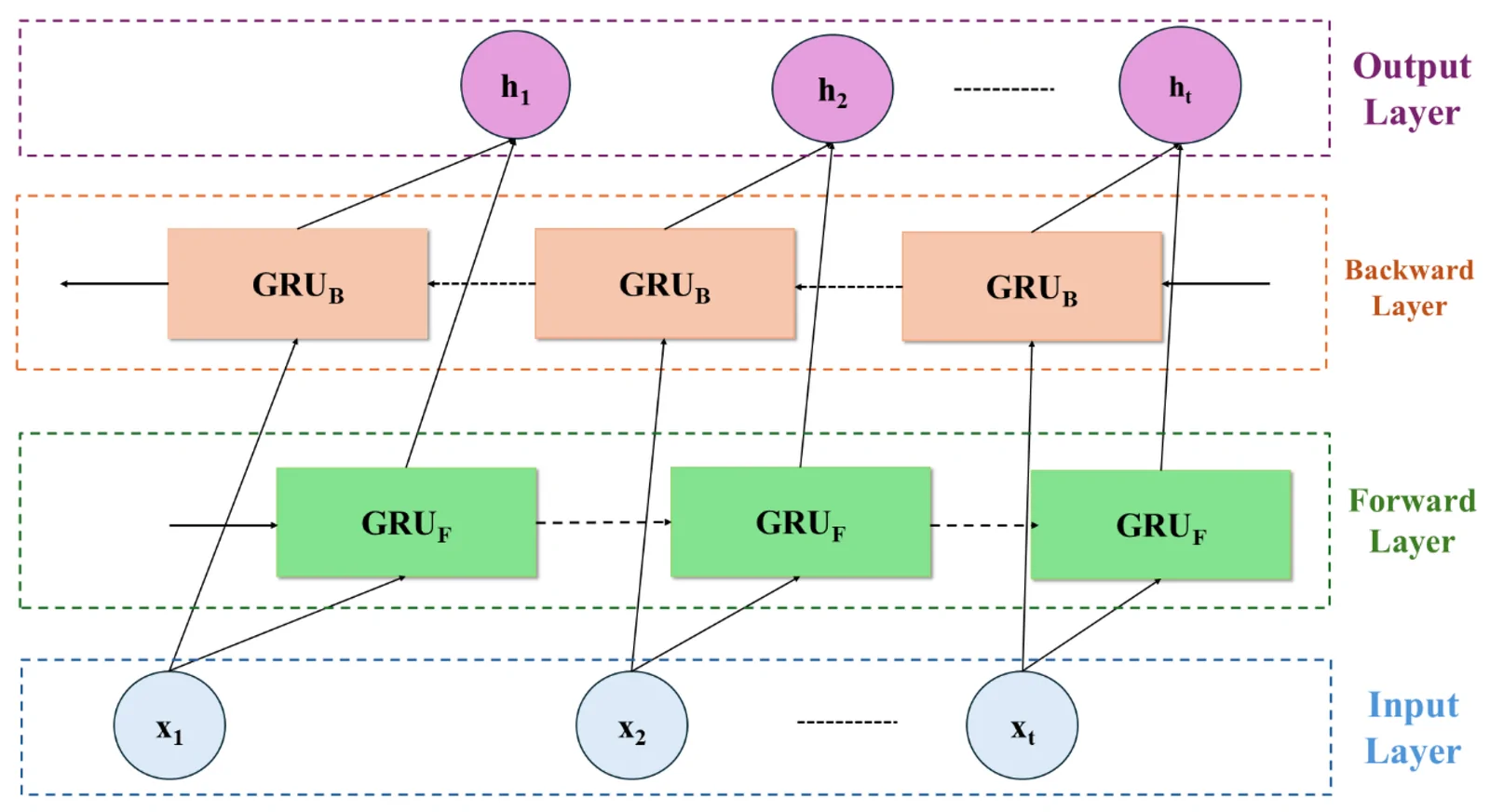
Conceptual illustration of Bidirectional GRU (Bi-GRU) architecture with forward/backward layers, based on the design described in Ma et al. [86].

The Bidirectional recurrent neural networks (BiRNNs) like BiLSTM and BiGRU enhance temporal sequence modeling by capturing dependencies in both forward and backward directions, resulting in a much richer context than unidirectional RNNs, which depend only on past contexts [80,85,86]. The Bi-GRU architecture with parallel forward and backward layers is illustrated in Figure 8. Such bidirectional learning is useful for analyzing geophysical time series data because deformation trends are affected both by previous trends and future developments. This was proven by Ma et al. [87], who used a BiGRU model to improve atmospheric delay corrections for InSAR measurements. In a related study, Wu et al. [88] combined CNN and BiGRU units in feature extraction, demonstrating their application in a prediction task. Collectively, these findings indicate that bidirectional structure improves model robustness, minimizes atmospheric and stochastic interference, preserves physical consistency in spatiotemporal deformation modeling, and thus makes the framework more suitable for subsidence prediction in dynamic environments [89,90,91].

### 4.3. Spatiotemporal Integration

#### 4.3.1. ConvLSTM Networks

**Figure 9:**
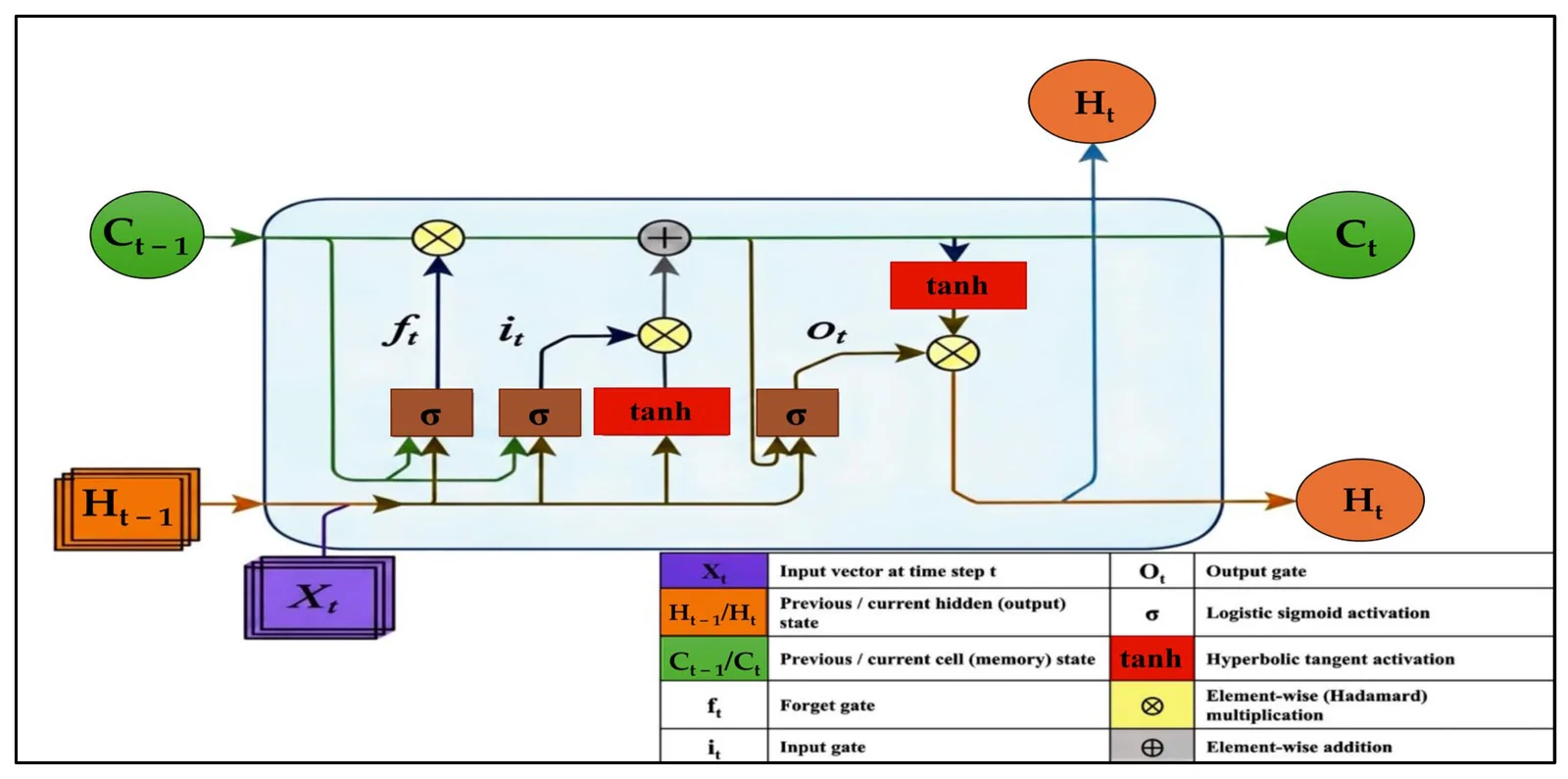
Original schematic of the ConvLSTM architecture illustrating convolutional replacement within LSTM gates, based on the concept in Leng et al. [93].

ConvLSTM networks form an elegant combination of the convolutional approach and recurrent neural network models, with the traditional fully connected layers in LSTM cells replaced by convolutional layers to concurrently learn spatial and temporal dependencies [92]. The ConvLSTM structural diagram showing convolutional replacement in LSTM gates is presented in Figure 9 [93]. As opposed to regular LSTM models, the ConvLSTM model employs the same gating mechanism while incorporating spatial features via convolutional layers, which allows it to simultaneously capture spatial correlations and temporal dynamics in spatiotemporal sequences. This type of neural network is especially suitable for subsidence prediction due to the presence of spatial correlation dependencies (the neighboring points move in a similar way) and temporal dependencies (accumulated deformation propagates over time).

ConvLSTM applications in subsidence prediction tasks have revealed many benefits compared to techniques that separately model spatial and temporal dimensions, by combining processing in space and time to better simulate deformation propagation mechanisms [94]. Domain knowledge-based physics-guided ConvLSTM models with tailored loss functions and architecture designs may prove beneficial in improving the physical fidelity of models and improving their generalization performance [95,96]. Convolution operations in the ConvLSTM cell contribute to learning invariant spatial features with respect to translations, so that the knowledge gained in one particular spatial area can be extended to other deformation zones.

Subsidence prediction at the regional scale by ConvLSTM has also been successfully shown, where ConvLSTM networks process sequences of displacement maps from InSAR to create deformation maps in the future with very wide extents of space [93]. The capacity of ConvLSTM to keep spatial resolutions high and perform time-based predictions makes it much more efficient than other pointwise prediction methods. Attention, residual learning, and multiscale processes have been added to advanced ConvLSTM structures to make them even better at predicting subsidence.

#### 4.3.2. CNN-LSTM Architectures

**Figure 10:**
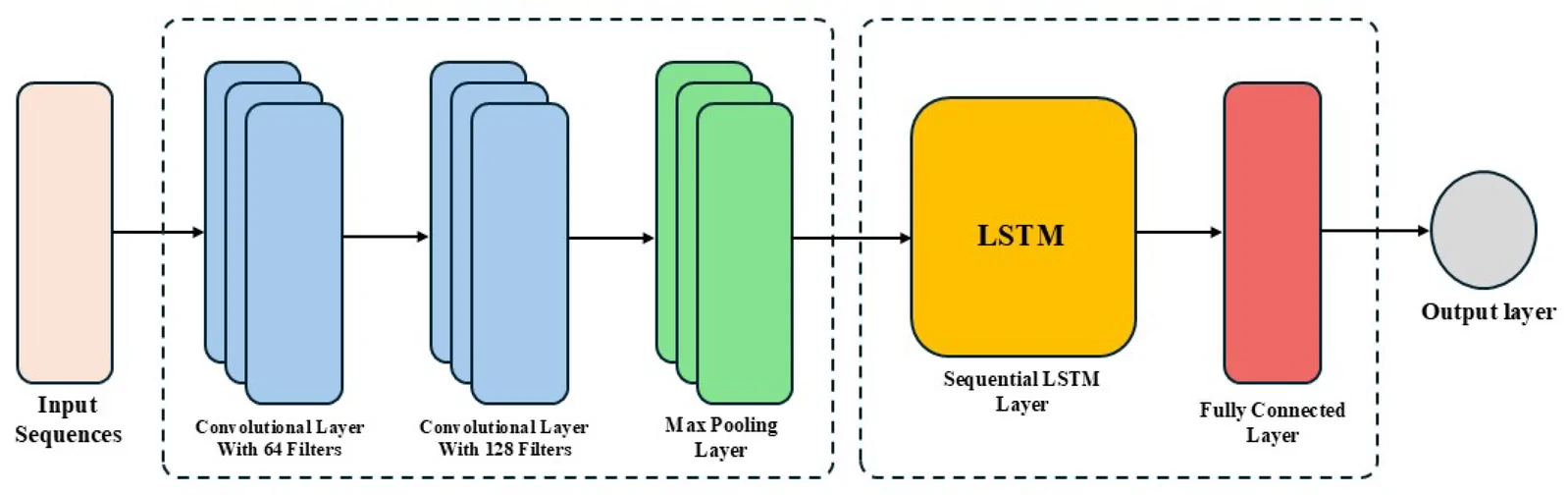
Conceptual illustration of the hybrid CNN-LSTM framework, based on the architecture described in Wang et al. [14].

To achieve a comprehensive, physics-consistent framework for land subsidence modeling, it is crucial to incorporate the localized spatial feature extraction and topological pattern recognition capabilities of Convolutional Neural Networks with the long-term temporal dependency learning and sequential forecasting strengths of LSTM networks. This integration results in the development of hybrid models called **CNN-LSTM models**, which have the capability to incorporate the spatial heterogeneity and temporal changes in the data for more robust prediction of land subsidence phenomena [97,98,99]. Such hybrid CNN-LSTM models generally consist of two major stages; the first stage uses convolutional layers to extract spatial features from InSAR displacement images or interferograms, whereas the second stage involves using LSTM layers to process these features and model temporal aspects of the data for forecasting future events. The CNN-LSTM framework demonstrating spatial feature extraction followed by temporal learning is shown in Figure 10 [14].

The implementation of the CNN-LSTM models in predicting nonlinear InSAR deformation has been proven to be extremely effective, where multiple research articles show that the model is capable of delivering precise multi-step-ahead predictions regarding nonlinear subsidence trends [23,100]. While the CNN model is effective at extracting spatial deformation gradients, subsidence funnel structures, and spatial anomalies, the LSTM model works well in predicting the temporal progression of these spatial factors in relation to the acceleration, deceleration, and eventual stabilization of subsidence development. Hybrid models based on dual-attention mechanism architecture for both CNN and LSTM layers have been developed for accurate predictions in nonlinear InSAR deformation cases [33].

Combination of CNN-BiLSTM architecture with the use of bidirectional recurrent neural networks for temporal data along with convolutional neural network layers for spatial data extraction is promising in predicting subsidence where high-resolution spatial data and temporal prediction are needed [99]. Bidirectional recurrent networks allow each prediction to be made using information both before and after the actual prediction, improving prediction and allowing retroactive analysis of the deformation process. The combination of attention models with CNN-BiLSTM results in enhanced models that weigh spatial data, temporal positions, and channels to extract optimum information from the subsidence data [101,102].

### 4.4. Advanced Hybrid Architectures with Attention Mechanisms

Attention mechanisms have become a central architectural component in modern deep learning models for land subsidence prediction, significantly enhancing the ability to capture and prioritize complex spatiotemporal dependencies from heterogeneous geospatial data. The core innovation lies in dynamically weighting the importance of different input features (e.g., spatial grids, temporal sequences, or influencing factors), allowing models to focus on the most relevant information for a given prediction task [32,103,104].

In particular, recent deep learning models for subsidence prediction employ attention mechanisms to dynamically adjust the weights of spatial and temporal features, leading to improvements over models that focus on relevant information [22,33].

Attention mechanisms enhance model performance by allowing neural networks to selectively focus on the most relevant information while suppressing noise and irrelevant patterns [22,102,105]. The dual-Attention-mechanism CNN-LSTM network (DACLnet) integrates CNNs for processing spatial features and LSTM networks for temporal learning, achieving an 85.09% and 68.57% reduction in RMSE compared with traditional LSTM and CNN-LSTM in the Turpan Basin over-extracted groundwater section [33].

Attention mechanisms demonstrate significant usefulness for heritage building displacement prediction studies. The combination of the LSTM model and attention with the SBAS-InSAR technique achieved a precise extraction of building clusters, with correlation coefficients of 0.95 and maximal subsidence rates of 60 mm/yr [26].

**Example of deformation prediction with the aid of deep learning based on InSAR deformation:**

**Figure 11:**
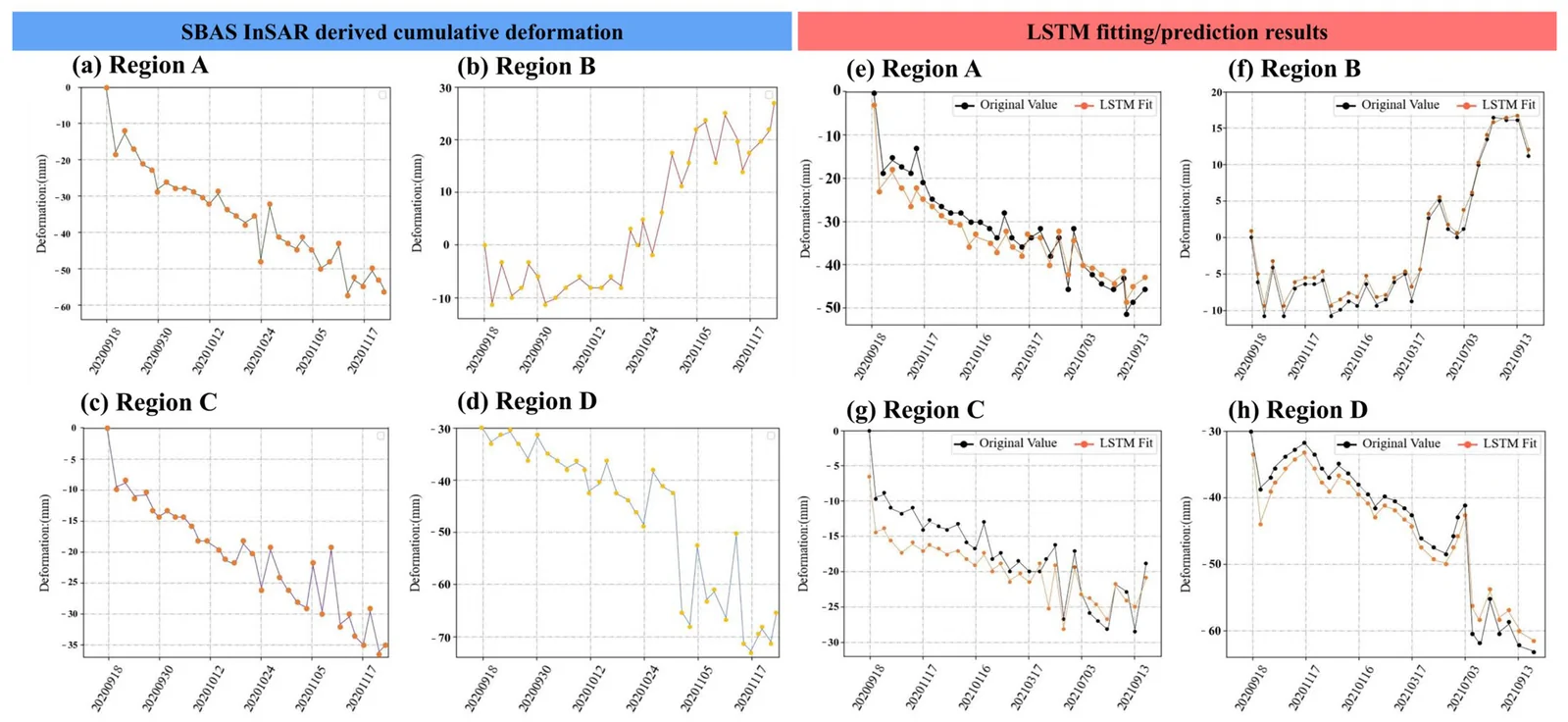
Example of LSTM-based deformation prediction using SBAS-InSAR-derived time-series observations: (**a**–**d**) cumulative time-series deformation for Regions A–D obtained from SBAS-InSAR; and (**e**–**h**) corresponding settlement fitting results using the LSTM model for Regions A–D. Reproduced from Ma and Lu [26] under a CC BY 4.0 license.

One example of deformation prediction based on real deformations inferred from the use of InSAR was carried out by Ma and Lu [26]. The work considered the use of Sentinel-1A (European Space Agency (ESA), Paris, France) SAR data processed via the SBAS-InSAR approach to infer the rates of spatial deformation and the deformation time series. The inferred deformation points and deformation time series served as the inputs for the LSTM-based prediction model. It is important to note that the SBAS-InSAR processing provided information about the heterogeneous spatial distribution of deformation, where there were areas of subsidence and uplift. In order to fit the deformation time series inferred from the InSAR data, the LSTM-based fitting was applied, as shown in Figure 11. From the comparison presented, it can be seen that the LSTM model was capable of fitting the observed deformation time series despite the presence of seasonal variations and noise in some regions. This study demonstrates the practical integration of real InSAR-derived deformation measurements with deep-learning-based time-series prediction.

The multi-head attention model, in which multiple parallel attention mechanisms are used to learn various temporal features, has proven effective in complicated forecasting tasks where multiple interacting processes are present [106]. Using the multi-head attention model for predicting subsidence in mines allows modeling of short-term changes, mid-term trends, and long-term developments, thus enabling the description of multi-scale temporal patterns. In addition, self-attention models, in which attention weights are calculated using correlations between various positions in one and the same sequence, can be used to forecast periodicity in subsidence series.

### 4.5. Integration with Temporal Decomposition Methods

In advancing subsidence prediction models, time-series decomposition methods such as variational mode decomposition (VMD) and singular spectrum analysis have been integrated with deep learning models to achieve more accurate results [29,101,107]. The VMD-SO-CNN-LSTM-MATT model decomposes the complex subsidence signal into stable intrinsic modes, which are represented by CNN-LSTM networks with Multi-Head Attention (MATT) and further optimized using the Snake Optimizer algorithm. This model increased the Mean Absolute Error (MAE), Root Mean Square Error (RMSE), and Mean Absolute Percentage Error (MAPE) by 29.85%, 8.42%, and 33.69%, respectively, indicating that it is a powerful method for predicting agricultural land subsidence in hilly regions [108].

In the mining cluster, the variational mode decomposition-singular spectrum analysis-Long Short-Term Memory (VMD-SSA-LSTM) model is introduced for surface subsidence analysis and provides high-precision time series forecasts. The rate of surface subsidence caused by mining, land-use changes, and geological conditions ranged from −34 mm/yr to 34 mm/yr in the mining areas of Jincheng City [29]. It is helpful to extract trend information from oscillations.

**Example of deep-learning-based prediction using InSAR-derived subsidence observations:**

**Figure 12:**
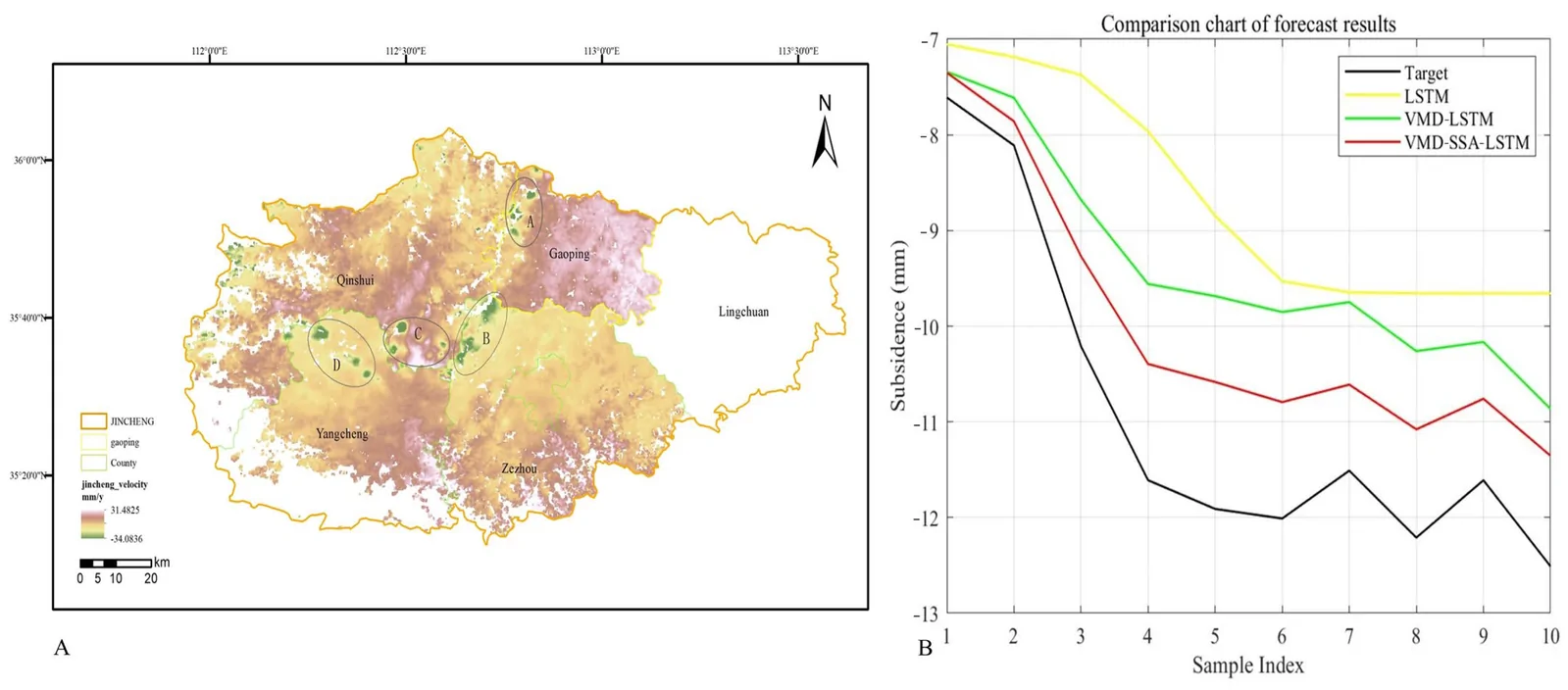
Representative example of deep-learning-based mining subsidence prediction using real InSAR-derived deformation observations: (**A**) spatial distribution of average surface deformation in the Jincheng City mining clusters (letters A–D indicate four specific high-subsidence mining regions: A-Gaoping, B-Yangcheng, C & D-Qinshui/Zezhou), and (**B**) comparison of target subsidence observations with LSTM, VMD-LSTM, and VMD-SSA-LSTM predictions. Reproduced from Yang et al. [29] under a CC BY-NC-ND 4.0 license.

An example of subsidence prediction due to mining activity based on real InSAR observations is illustrated in Figure 12. Yang et al. [29] conducted an assessment of surface deformation due to mining activities in Jincheng City mining clusters with Sentinel-1 time-series InSAR observations in the period 2018–2021. The distribution of the obtained average surface deformation shows clear subsidence areas related to the mining clusters (Figure 12A). The time-series observations of subsidence derived from InSAR observations were used in this study for evaluating the deep-learning-based prediction models. For conducting prediction experiments, time-series subsidence observations in the Yangcheng County area of the Jincheng mining cluster were chosen. The conventional LSTM, VMD-LSTM, and VMD-SSA-LSTM models were evaluated. The predicted results show that the prediction of the VMD-SSA-LSTM model agrees better with target observations of the subsidence (Figure 12B). This study showed RMSE, MAE, and MAPE of the test set equal to 0.58664 mm, 0.5647 mm, and 5.2252%, respectively, for the VMD-SSA-LSTM model. This example demonstrates how InSAR-derived deformation measurements can be directly integrated with deep-learning models to characterize and predict the temporal evolution of mining-induced subsidence.

### 4.6. Transformer-Based Architectures for Subsidence Prediction

**Figure 13:**
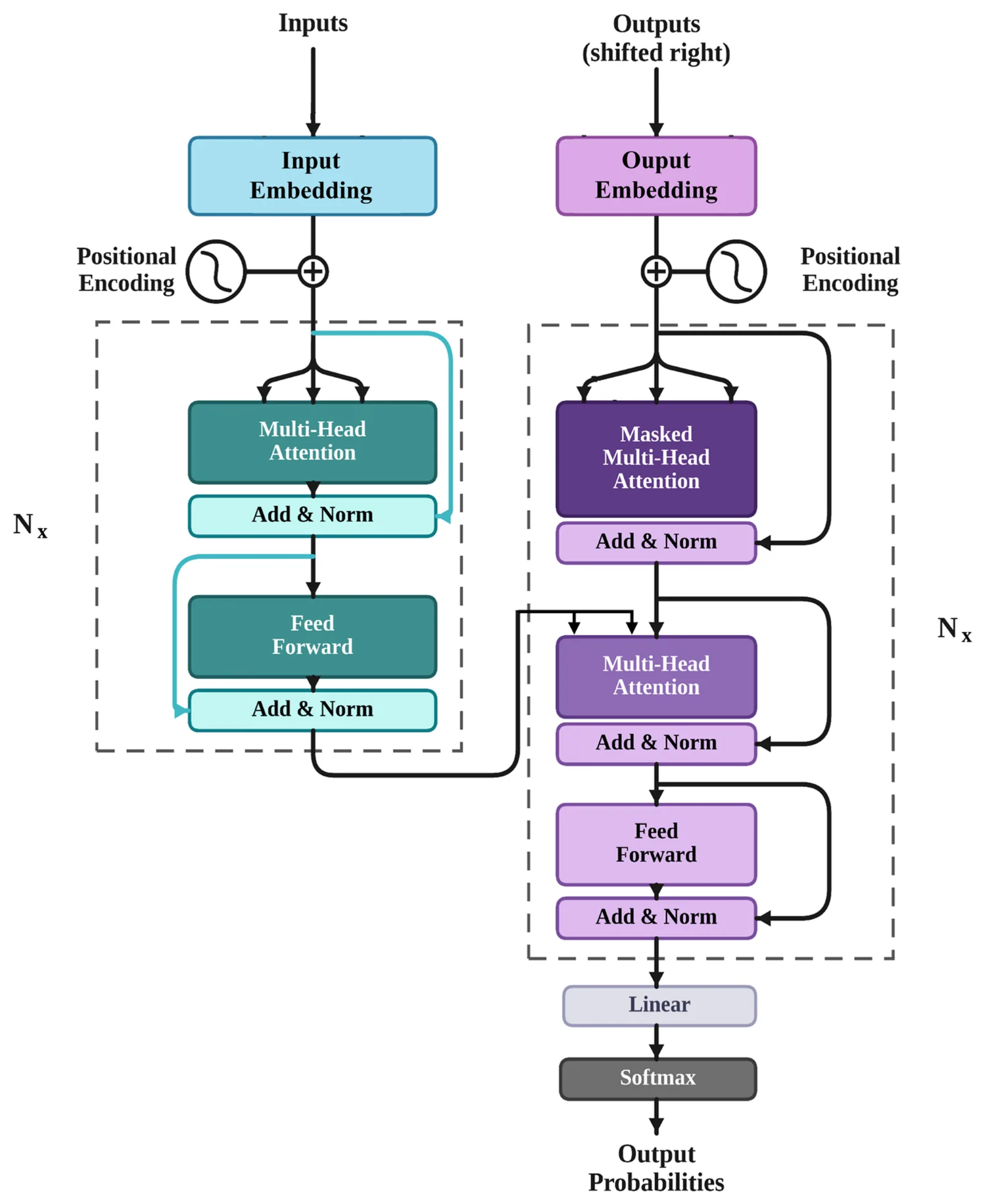
Original schematic of the standard Transformer encoder-decoder architecture, based on the design described in Vaswani et al. [109].

The introduction of the Transformer architecture by Vaswani et al. [109] constitutes a breakthrough in the shift of paradigms from recurrent models to an exclusive focus on the attention-based approach in modeling sequences devoid of any dependency issues, allowing computations in parallel for all timestamps. The transformer model makes use of only self-attention networks alongside positionwise feed-forward networks in an encoder-decoder format consisting of multiple layers of neural networks, layer normalization, and residual connections [55,110]. The standard Transformer architecture with encoder-decoder stacks and multi-head self-attention is illustrated in Figure 13 [109]. This architecture effectively addresses the limitations of previous sequence-processing models, such as recurrent neural networks (RNNs) [67] and long short-term memory networks (LSTMs) [19], by eliminating the need for sequential data processing.

Transformers specifically designed for time-series forecasting, such as the Informer model, make use of advancements like the ProbSparse attention mechanism for efficient computation, self-attention distillation mechanisms for feature learning, and generative-style decoder models for multi-step forecasting [111]. These modern Transformers have proved their superiority over LSTM models in time-series forecasting, having achieved impressive results on various benchmark tests. The application of Transformers in the prediction of land subsidence has been an emerging topic of research in recent times, with many papers demonstrating success in applying GNSS troposphere products, InSAR time-series deformation, and Transformer models [50,112].

The Spatiotemporal Transformer model (SpacetimeFormer) is an extension of the basic transformers for the purpose of analyzing two dimensions–spatiotemporal analysis using the techniques of spatiotemporal attention. Such an approach is ideal for studying subsidence cases where deformation patterns show correlations in space among nearby points as well as evolve over time in complicated ways [113]. The use of transfer learning based on pre-trained transformer models has also been considered for recognizing patterns of deformations [36].

### 4.7. Graph Neural Networks (GNNs)

Graph Neural Networks (GNNs) constitute a newly proposed type of deep learning architecture that learns to operate on graph-based data, thus making it possible to account for spatial irregularities and network structures that cannot be described effectively in terms of regular grid data [114,115]. In the problem of predicting subsidence processes, GNNs may account for spatial correlations in relation to irregularly located measuring points like GNSS points or PS-InSAR points by representing the data in the form of a graph whose nodes correspond to measurement points and edges indicate spatial proximity or geological similarity [116].

**Figure 14:**
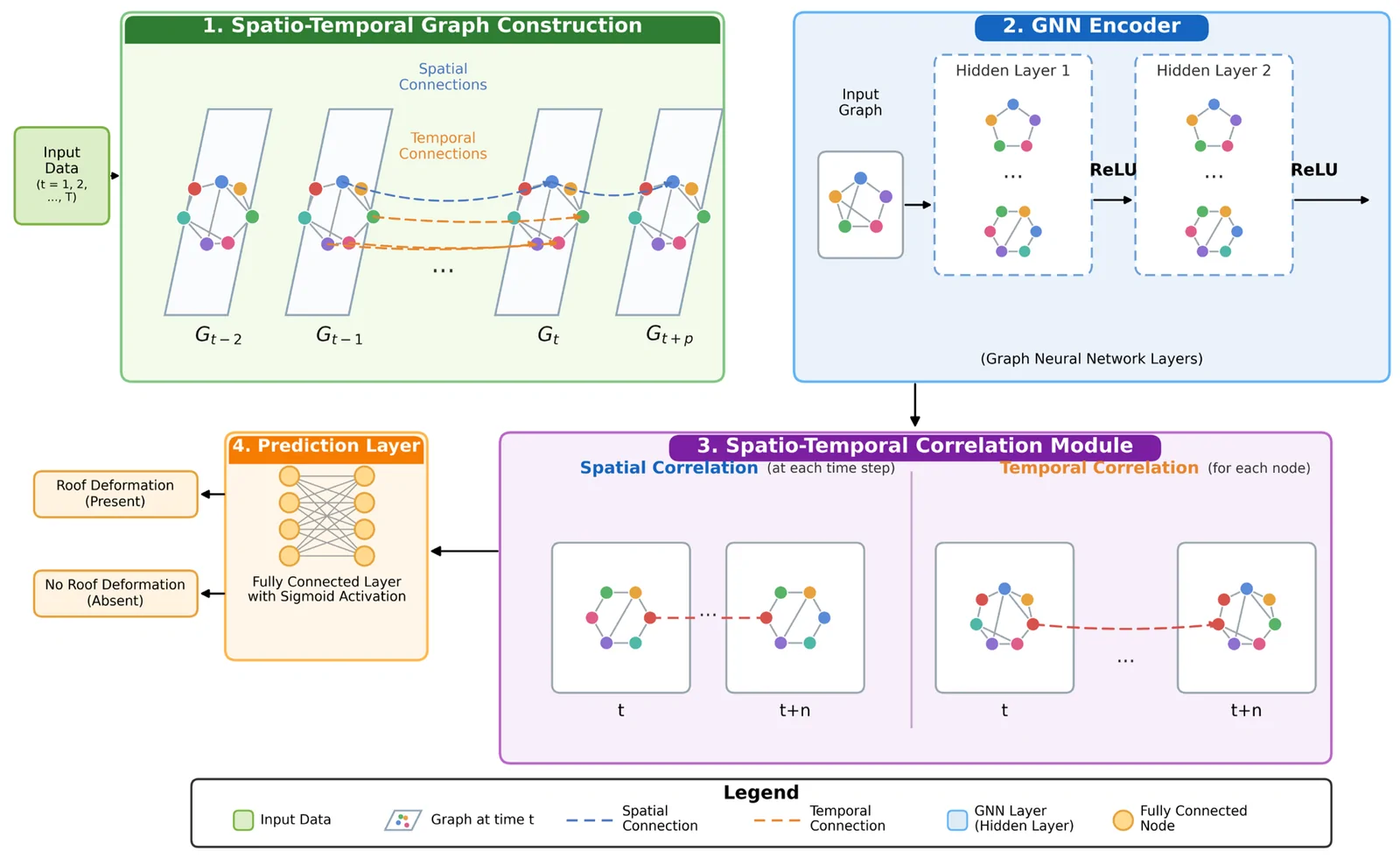
Conceptual illustration of a spatio-temporal graph neural network (ST-GNN) framework, based on the concept described in Zhang [115].

The use of Graph Convolutional Network (GCN) models combined with GRU neural networks for predicting landslide displacements has been successful in validating the potential of spatiotemporal graph-based methods in geohazard prediction [24]. The spatiotemporal graph neural network (ST-GNN) framework for mining-induced roof deformation prediction is shown in Figure 14 [115]. Attentive Graph Neural Networks, which adaptively allocate edge weights using spatiotemporal correlations, can be used to predict deformations based on GNSS data [117]. The combination of GNN models with InSAR deformations allows for multiple relationships to be merged and predictions made, considering the geological heterogeneity and stress redistribution caused by mining [118,119].

Prediction of effective elastic moduli of rocks using Graph Neural Networks: Graph-based deep learning methods specifically designed for predicting landslides show better performance than simple single-point time-series approaches, highlighting the importance of incorporating spatial context and inter-point correlations [116]. The flexibility of GNN architectures to deal with irregular sampling, including different types of edges with geological/geophysical features, and varying topology depending on the application scenario makes these models more appropriate for complicated subsidence problems that involve multiple mining panels and non-uniform overburdens [120,121].

### 4.8. Multi-Scale Spatiotemporal Modeling

Multi-scale Spatiotemporal Modeling refers to a framework that addresses the complex, nonlinear relationships between deformation and its driving factors across different spatial and temporal scales [122,123,124]. Majda and Chen [125] establish the foundational theory for “complex multiscale systems” and “reduced-order nonlinear modeling strategies,” directly supporting the nonlinear relationships component. The MGTWR-CNN-BiLSTM-AM model identifies subsidence as a spatially variable process that is conditioned on local factors and calls for multi-scale analysis. The MGTWR-CNN-BiLSTM-AM model is such a model that synchronizes monthly and annual analysis, while considering monthly groundwater extraction and precipitation, but only considering land use and soil type on an annual basis. This model enhanced predictive accuracy up to 0.99347 (RMSE: 1.8643 mm) for Shanghai, revealing deformation contributors [32].

The Sparrow Search Algorithm–Convolutional Neural Network–Long Short-Term Memory Model (SSA-CNN-LSTM) approach is another multiple-scale method for urban surface deformation prediction, utilized in Hohhot city. It also included ground temperatures at three depths (5 cm, 10 cm, and 15 cm). This approach optimized CNN-LSTM hyperparameters in the course of training and obtained correlation coefficients of 0.92, 0.83 and 0.93 at different monitoring points. It showed better performance than conventional models [82]. Inclusion of the subsurface temperature measurements was found to be important for soil-deformation relationships.

**Figure 15:**
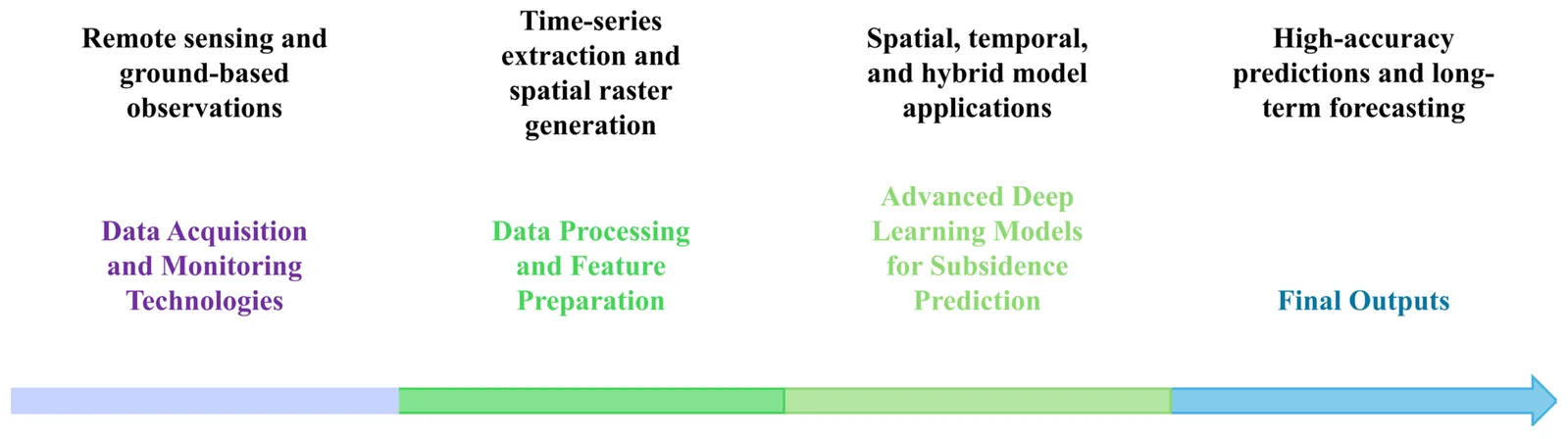
End-to-end framework for subsidence prediction illustrating data acquisition, data preprocessing and feature preparation, advanced learning-based modeling, and final prediction outputs.

An end-to-end framework for subsidence prediction, illustrating data acquisition, preprocessing, modeling, and prediction outputs, is presented in Figure 15.

## 5. Methodological Advances in Deep Learning for Subsidence Prediction

Recently, methodological advancements in deep learning have substantially revolutionized the prediction of land subsidence by enabling reliable, interpretable, and scalable modeling. This advance is driven by the coupling of neural architectures with high-resolution geospatial datasets, particularly Interferometric Synthetic Aperture Radar (InSAR)-derived measurements of ground deformation. Deep learning applied to InSAR data has enabled enhanced modeling of complex subsidence processes across diverse geological and anthropogenic environments.

The methods reviewed here correspond to the enhancement strategies recorded in Section 2.6, which can be applied to any of the six architecture classes: attention augmentation (Section 5.3), hyperparameter optimization and transfer learning (Section 5.5), ensembling (Section 5.6), physics-informed constraints (Section 5.7) and signal decomposition and feature engineering (Section 5.8).

This section discusses the working principles and advanced implementation context of the deep learning models adopted by the authors for prediction. For each model family, the discussion covers the fundamental mathematical operations, architectural design choices, rationale for adoption in the subsidence prediction context, and the specific ways researchers have implemented and adapted these models. The section is organized according to architectural complexity and progress.

Consistent with the scope defined in Section 4, the ‘Working Principles’ subsections below present only the equations and formulation details that are adapted, modified, or newly combined for subsidence prediction (each explicitly marked as such); they are not a restatement of the general architecture already introduced in Section 4. The ‘Adoption’ subsections that follow each architecture then summarize how that formulation has been applied in subsidence-related studies specifically.

### 5.1. Hybrid Deep Learning Architectures for Spatio-Temporal Modeling

#### 5.1.1. CNN-LSTM Models

(a) **Working Principles**

The CNN-LSTM model is a type of hybrid model that integrates the ability of CNNs to perform spatial feature extraction with the temporal modeling capability of LSTMs. The working principle of CNN-LSTM is based on a cascaded structure that comprises two stages:

**Stage 1 (Spatial Feature Extraction)**: At each time step, the CNN module processes a spatial input (which could be a gridded map of groundwater levels, a geological feature raster, or an InSAR displacement image) through convolutional and pooling layers, obtaining a compact feature vector that represents the most important patterns in the image.

**Stage 2 (Temporal Sequence Modeling)**: The CNN module processes the sequence of feature vectors from several time steps, passing the feature vector sequences to the LSTM module. The LSTM module’s hidden state encodes the temporal evolution of the spatial feature vector, allowing the model to forecast future spatial states from the history of the spatial feature vector.

The mathematical formulation can be expressed as:

(1) $${\hat{y}}_{t}=g\left(\phi_{\mathrm{LSTM}}\left(h_{t-1},\;\phi_{\mathrm{CNN}}\left(X_{t}\right)\right)\right)$$

where *X_t_* is the spatial input at time step *t*, *h_t_*_−1_ is the LSTM hidden state, $\hat{y_{t}}$ is the predicted output and *ϕ_CNN_* and *ϕ_LSTM_* represent mapping functions.

Equation (1) is modified and adapted from [16].

(b) **Adoption of CNN-LSTM**

The prediction performance of the CNN-LSTM model was satisfactory in practice, such as in a mining subsidence prediction project where CNN was adopted to extract features and LSTM to extract features on the surface with respect to time [14]. The proposed model can break through the limitation of applying a single LSTM model to predict spatiotemporal features in other positions and obtain low prediction accuracy. Based on the prediction results, it can be seen that the ranking of model performance was CNN–LSTM (R^2^ = 0.98–0.99) > LSTM (R^2^ = 0.95–0.98) > CNN (R^2^ = 0.75–0.97). Compared with single models, the proposed model benefited from an increase of 0.24 in R^2^ value and a reduction of 0.02 mm in RMSE and 0.01 mm in MAE.

#### 5.1.2. Convolutional LSTM (ConvLSTM) Networks

(a) **Working Principles**

Convolutional LSTM Networks are proposed by Shi et al. [92] and can be considered a more integrated spatio-temporal model where fully connected operations in a conventional LSTM network are replaced with convolutional operations. This allows a ConvLSTM network to handle both spatial and temporal information simultaneously within a single cell, avoiding the need for a separate feature-extraction step in a conventional CNN. In a ConvLSTM, all inputs (*X_t_*), cell outputs (*C_t_*), hidden states (*H_t_*), and internal gates (*i_t_*, *f_t_*, *o_t_*) are strictly maintained as 3D tensors (where the last two dimensions denote spatial rows and columns).

The mathematical formulation of ConvLSTM, expressed in Equations (2)–(4), follows the spatiotemporal extension of standard LSTM gates proposed by Shi et al. [92].

(2) $${\left(i_{t},f_{t}\right)}^{T}=\sigma \left({\left(W_{\mathrm{xi}},W_{\mathrm{xf}}\right)}^{T}*X_{t}+{\left(W_{\mathrm{hi}},W_{\mathrm{hf}}\right)}^{T}*H_{t-1}+{\left(W_{\mathrm{ci}},W_{\mathrm{cf}}\right)}^{T}\circ C_{t-1}+{\left(b_{i},b_{f}\right)}^{T}\right)$$

*C_t_* = *f_t_* ∘ *C_t_*_−1_ + *i_t_* ∘ *tanh* (*W_xc_* ∗ *X_t_* + *W_hc_* ∗ *H_t_*_−1_ + *b_c_*) (3)

*o_t_* = σ(*W_xo_* ∗ *X_t_* + *W_ho_* ∗ *H_t_*_−1_ + *W_co_* ∘ *C_t_* + *b_o_*) (4)

where ∗ denotes the convolution operation (replacing matrix multiplication in standard LSTMs), ∘ denotes element-wise multiplication.

(b) **Adoption for ConvLSTM**

ConvLSTM was initially introduced by Shi et al. [92] for precipitation nowcasting, achieving better results than FC-LSTM and the ROVER optical-flow baseline. Its application to subsidence is straightforward, since InSAR products are already on a grid: Leng et al. [93] used ConvLSTM for spatio-temporal prediction of regional land subsidence, and Yao et al. [94] for forecasting mining-area surface deformation from SBAS-InSAR time series, both inputting successive displacement maps as 3-D tensors to let the model handle spatial neighborhoods and temporal dynamics in the same cell, instead of first using a CNN to extract spatial features and then an LSTM to model temporal dynamics.

#### 5.1.3. Other Spatio-Temporal Models

Zhu et al. [99] proposed a CNN-BiGRU-Attention hybrid model for the spatial and temporal prediction of subsidence in the mining area. In the proposed model, the CNN layers are used to learn the local temporal features of each subsidence time series with a small convolutional kernel and pooling operation, in which noise is removed and dimensionality is reduced while maintaining the major deformation patterns. The learned local temporal features are then input into the proposed BiGRU network to better encode the dynamic changes at different times, such as short-term fluctuations and long-term subsidence trends.

### 5.2. Temporal Convolutional Networks

(a) **Working Principles**

Bai et al. [126] made use of Temporal Convolutional Networks (TCNs), which are considered to be an alternative to traditional RNNs, as the network replaces the recurrent structure with one-dimensional convolutions over the temporal dimension. TCNs involve the incorporation of two fundamental design features that allow the network to effectively model the sequence information. These features are as follows:

i. **Causal convolutions:** In the TCNs, the convolution operation is made causal such that the network is allowed to use the information from the present as well as the past instances, without any information from the future instances being considered while making the predictions at the current instance t. This is accomplished through the appropriate padding of the output of the convolution operation.
ii. **Dilated convolutions:** Although the use of causal convolutions would allow the network to consider the information from the present as well as the past instances, the network would be able to consider the information from the past instances to a limited extent as the depth of the network increases linearly with the history size. In order to overcome the problem of linear growth of the history size with the network depth, the use of Dilated Convolutions is made as per the study of [127], which enable an exponentially large receptive field [128].

Formally, given a one-dimensional input sequence *x* ∈ ℝ*^n^* and a convolutional filter *f*: 0, …, *k* − 1 → ℝ, the dilated convolution operation *F* evaluated at position *s* in the sequence is defined as:

(5) $$F_{s}=\sum_{i=0}^{k-1}f_{i}\cdot x_{s-d\cdot i}$$

Equation (5) is adapted and reformulated from Bai et al. [126].

**Figure 16:**
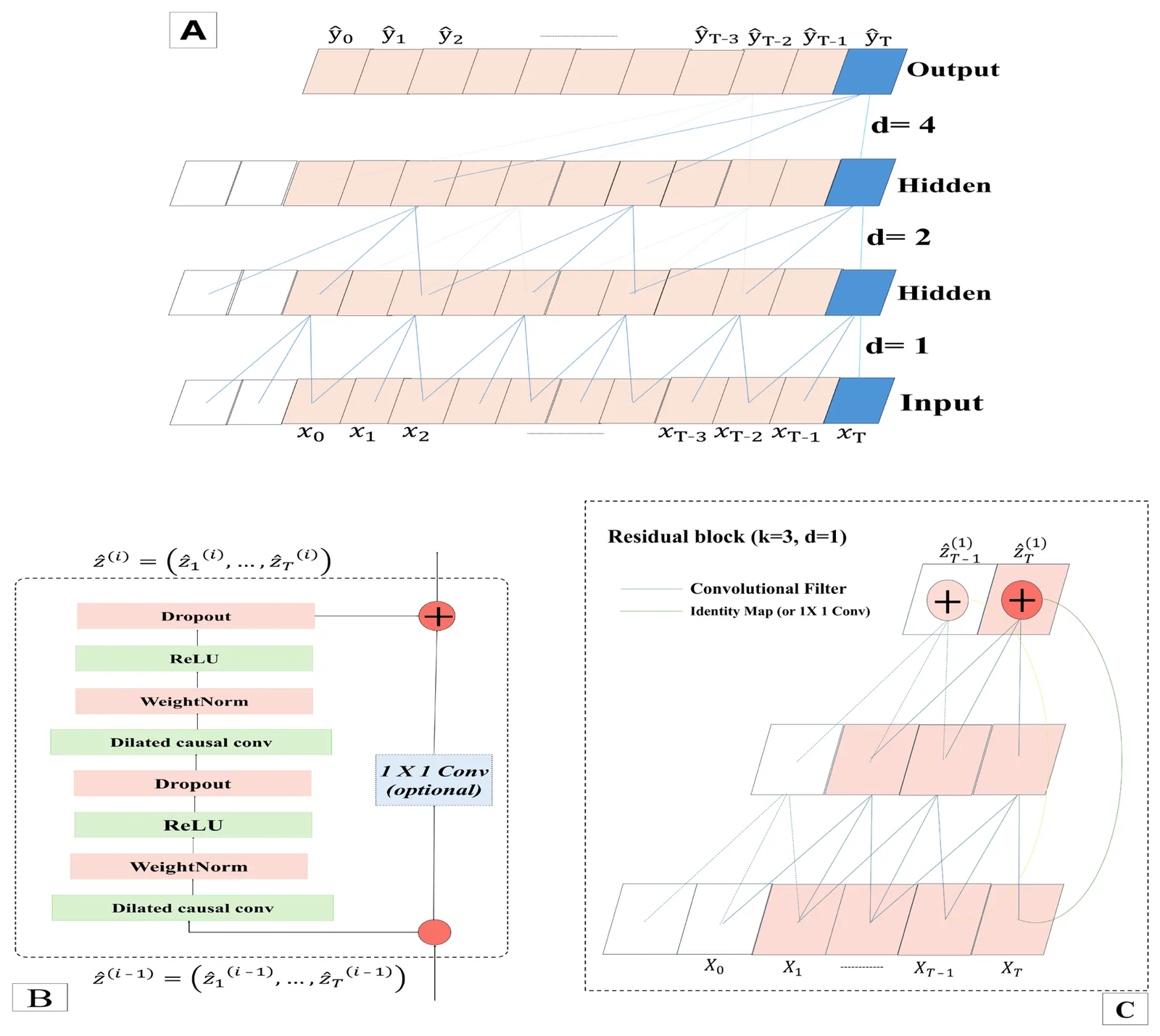
Architectural elements in a TCN. (**A**) A dilated causal convolution with dilation factors d = 1, 2, 4 and filter size k = 3. The receptive field is able to cover all values in the input sequence. (**B**) TCN residual block. A 1 × 1 convolution is added when the residual input and output have different dimensions. (**C**) An example of a residual connection in a TCN. The blue lines are filters in the residual function, and the green lines are identity mappings. Original schematic illustrating dilated causal convolutions and residual connections in a Temporal Convolutional Network, based on the architectural concept described in Bai et al. [126].

The internal structural pipeline and fundamental configuration elements of a Temporal Convolutional Network are schematically illustrated in Figure 16.

Here, *d* denotes the dilation factor, *k* represents the filter size, and the term *s* − *d* ⋅ *i* ensures the convolution operates over past elements. Dilation enables setting a constant spacing between adjacent filter values; when *d* = 1, the operation reduces to a standard convolution. By using larger dilation rates, the high-level features can leverage information from wider inputs, thus increasing the receptive field size of the convolutional model.

TCN architectures typically incorporate residual connections that add the input of each block directly to its output, enabling gradient flow through the network and allowing very deep architectures to be trained effectively. To accommodate extreme depth without gradient degradation, TCNs heavily utilize residual blocks. If *x* is the input of the residual block, the output *O* is expressed as:

Each dilated convolutional stack is wrapped in a residual block *O* = *ϕ*(*x* + ℛ(*x*)), following He et al. [55], with a 1 × 1 convolution applied to x when input and output channel widths differ (Figure 16B,C); this permits deep stacks to be trained without gradient degradation [126]. Because residual additions require mathematically equivalent tensor shapes, the TCN employs an auxiliary 1 × 1 convolution to the input *x* whenever the input and output channel widths differ, ensuring shape alignment before addition. By decoupling the temporal dimension from the sequential computation path, TCNs avoid the vanishing gradient problem entirely, optimize heavily on GPU architectures via parallel execution, and accommodate arbitrary-length sequence inputs.

(b) **Adoption of Temporal Convolutional Networks**

Liu et al. [129] propose a new hybrid neural network model named TCN-GRU for short-term deformation prediction. TCN module generates deep spatial features from time-series data in the first stage, after which the GRU module processes those generated deep spatial features to obtain final predictions. This two-stage framework can mitigate the deficiency of single neural networks that fail to model complex deformation patterns. This coupling method overcomes single-model limitations (LSTM, RNN or stand-alone CNN) by integrating TCN’s efficient feature extraction with GRU’s imitation of temporal memory.

(c) **Key components**
  i. **TCN Architecture Features:**
    - Causal convolutions prevent information leakage by enforcing temporal data processing order
    - Dilated convolutions provide flexible receptive field control and enhanced temporal memory for longer historical sequences.
    - Residual connections replace standard convolutional layers to ensure network stability in deeper architectures.
  ii. **GRU Integration:**
    - Gate mechanisms control data feature flow, addressing long-term memory and gradient problems in traditional RNNs

**Figure 17:**
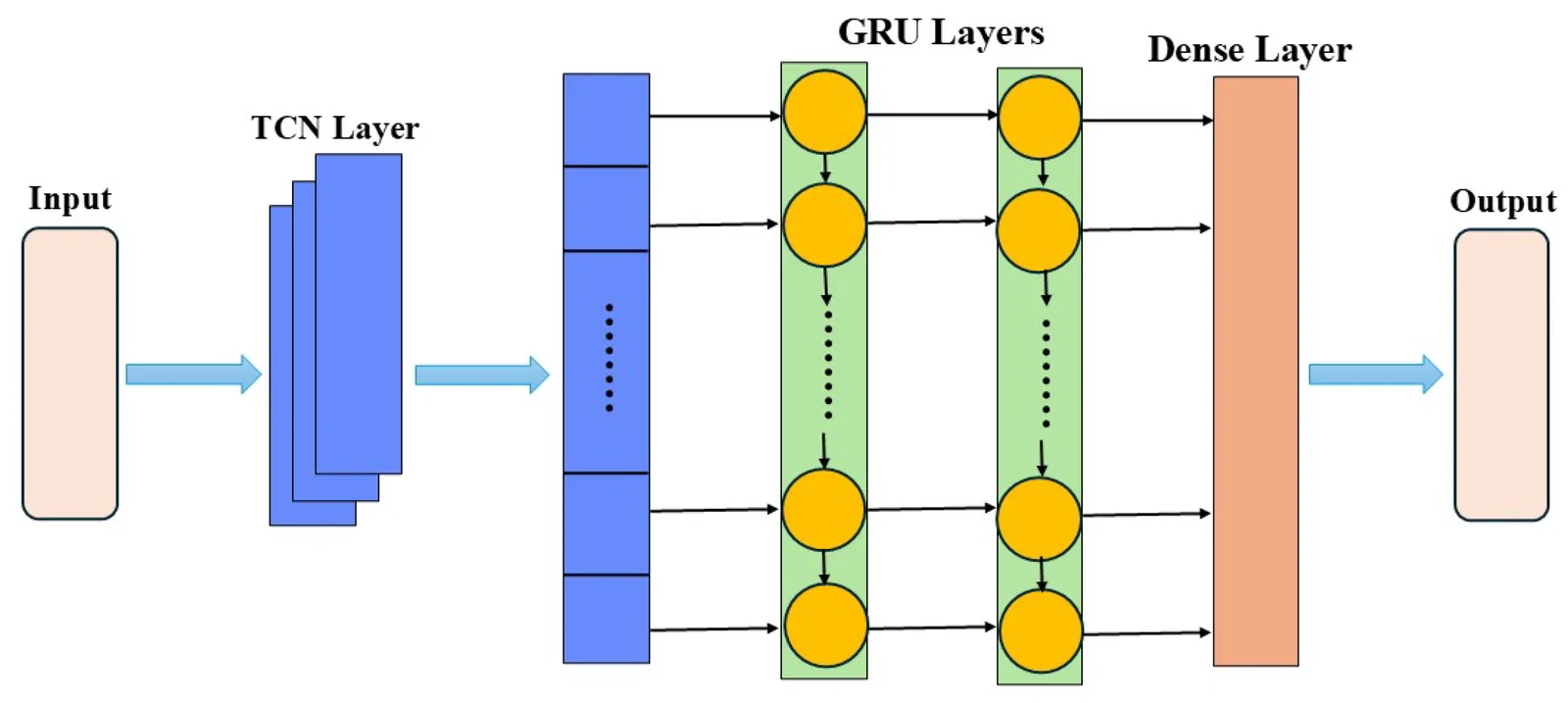
Conceptual illustration of the hybrid TCN–GRU model integrating convolutional temporal feature extraction with recurrent sequence learning for improved time-series prediction accuracy described in Liu et al. [129].

The complete workflow map and layer-wise data routing strategy of the integrated TCN-GRU prediction framework are visualized in Figure 17.

The proposed TCN-GRU model achieved the best performance compared with traditional single models (RNN, LSTM, GRU, TCN) at three test locations within Kunming city. For point K2, the hybrid MAE was 1.82 mm, and the RMSE was 2.48 mm; for point K3, the hybrid model reached a MAE of 2.60 mm and an RMSE of 3.04 mm. The model demonstrated enhanced performance when tested in high-volatility datasets (i.e., K1 point and K2 point) compared to individual TCN and GRU models.

### 5.3. Attention Mechanisms Based Models

(a) **Working Principles of Attention Mechanisms**

Attention mechanisms represent a transformative methodological advance that addresses a fundamental limitation of standard encoder-decoder architectures: the information bottleneck created by compressing the entire input sequence into a single fixed-length context vector. For subsidence prediction, where input sequences may span years of Observations containing complex multi-scale temporal patterns, this compression can result in significant information loss, particularly for long sequences [130].

The attention mechanism computes a dynamic, input-dependent context vector for each decoding Step by learning to selectively focus on the most relevant portions of the encoder’s output sequence. The mechanism operates through three steps:

i. **Score computation**: For each decoder time step *t* and each encoder hidden state *s_k_*, an alignment score is computed that measures the relevance of the encoder state to the current decoding position:

*e_t_*_,*k*_ = *score*(*h_t_*_−1_, *s_k_*) (6)

ii. **Attention weight computation**: The alignment scores are normalized using the SoftMax function to produce attention weights that sum to one:

(7) $$\alpha_{t,k}=\frac{\exp\left(e_{t,k}\right)}{\sum_{j=1}^{T_{x}}\exp\left(e_{\mathrm{tj}}\right)}$$

iii. **Context Vector Computation**: The context vector *c_t_* is the weighted sum of the encoder states:

(8) $$c_{t}=\sum_{k=1}^{N}\alpha_{t,k}s_{k}$$

Equations (6)–(8) present a modified and generalized version of the additive attention framework proposed by Bahdanau et al. [130].

The Context vector changes from one decoding stage to another and thus gives the decoder direct access to the relevant parts of the input sequence, thereby helping the model to “focus” on different parts of the input for different parts of the output generated. In the case of subsidence prediction, the attention mechanism helps the model learn which time periods in the past are most relevant to predict subsidence in the future.

(b) **Adoption of attention mechanisms**

According to Wang et al. [106], the MHAFFM, which integrates multi-head attention with Multi-linear regression (MLR), will take advantage of the capabilities of both approaches to achieve better prediction results. The multi-head attention component can exploit its strength of being able to pay more attention to different parts of the input data and can be combined with MLR since the latter provides an easy-to-understand method of building a relationship between variables. Such a model would solve problems faced by current models.

Liu and Zhang [22] adopted an attention-based Long Short-Term Memory (AT-LSTM) to capture long-term subsidence relationships (LTS-R) for ground subsidence forecasting. The attention mechanism was added so that we could selectively attend to temporal information and highlight important time steps. While the original LSTM treats all time dependencies as being equally important, the attention mechanism weights certain time points as being more relevant than others to the prediction process. This mechanism works by:

- A fully connected layer that trains attention weights from LSTM hidden layer outputs
- SoftMax normalization to generate importance weights for each hidden state vector
- Weighted summation that emphasizes more influential temporal features

This allows the model to recognize that dependence on information at different times varies, with more temporally proximate influences being more significant.

### 5.4. Transformer Models

(a) **Working Principles of Transformer Model**

The Transformer architecture, introduced by Vaswani et al. [109], represents a paradigm shift in sequence modeling by replacing recurrence entirely with self-attention mechanisms. This architectural innovation eliminates the sequential processing constraint of RNNs, enabling full parallelization of computation and more efficient capture of long-range dependencies.

The core building block of the Transformer is the **multi-head self-attention** mechanism, which operates as follows. For each input token, query, key and value vectors are obtained by learned linear projections of the input matrix *Q* = *XW^Q^*, *K* = *XW^K^*, *V* = *XW^V^* [109]. Attention is then computed as Equation (9)

(9) $$\mathrm{Attention}\left(Q,K,V\right)=\mathrm{softmax}\left(QK^{T}/\sqrt{d_{k}}\right)V$$

and multiple heads are computed in parallel. The resulting head outputs are concatenated and subsequently projected through a learned output matrix, as formulated in Equation (10).

(10) $$\mathrm{MultiHead}\left(Q,K,V\right)=\mathrm{Concat}\left(\mathrm{hea}d_{i}\right)W^{O},\;\mathrm{where},\mathrm{each}\mathrm{hea}d_{i}=\mathrm{Attention}\left(QW_{i}^{Q},KW_{i}^{K},VW_{i}^{V}\right)$$

A complete encoder layer wraps the multi-head sub-layer and a position-wise feed-forward network in residual connections [55] with layer normalisation [110]; since self-attention is permutation-invariant, positional encodings are added to the inputs [109].

The Transformer’s ability to compute attention between all pairs of sequence elements in parallel rather than sequentially as in RNNs confers two critical advantages: (1) computational efficiency through parallelization, enabling faster training on modern GPU hardware, and (2) constant path length between any two positions in the sequence, facilitating the learning of long-range dependencies without the gradient propagation challenges that affect recurrent networks [109].

(b) **Adoption of the transformer model**

Wang et al. [45] adopt a supervised **Vision Transformer** (ViT-Base) classifier, pretrained on ImageNet and fine-tuned to classify subsidence severity, using gridded, ground-measured subsidence labels paired with high-resolution optical satellite image patches. The model leverages dense grid measurements from a well-surveyed mine to learn the subtle spectral–textural patterns associated with different subsidence levels, exploiting ViT’s global self-attention mechanism to capture spatial dependencies between neighboring parcels. The trained classifier is then transferred to other coalfields using only single-epoch high-resolution optical imagery and coarse subsidence labels, thereby testing its cross-regional robustness. The findings demonstrate meaningful generalization potential.

The Geo-Temporal Transformer (GTformer) integrates a Geographically and Temporally Weighted (GTW) layer with a standard Transformer encoder to model land subsidence. The GTW layer constructs spatiotemporal distance matrices from geographical coordinates and temporal dimensions, weighting input features by spatiotemporal proximity using a Gaussian kernel, thereby capturing spatiotemporal heterogeneity. This is followed by multi-head attention, feedforward networks, and a linear output layer to capture nonlinear relationships and long-term sequential dependencies. Unlike traditional models that rely solely on historical subsidence data, GTformer incorporates multiple environmental factors, including natural factors (precipitation, changes in groundwater storage) and urbanization factors (metro distance, POI density, population density, road density, impermeable surface area). GTformer achieved an R^2^ of 0.989, representing improvements of 14.6% over LSTM and 4% over standard Transformer models, with a Mean Squared Error of only 0.109 mm and 92.24% of predicted areas attaining an R^2^ greater than 0.6 [27].

**Figure 18:**
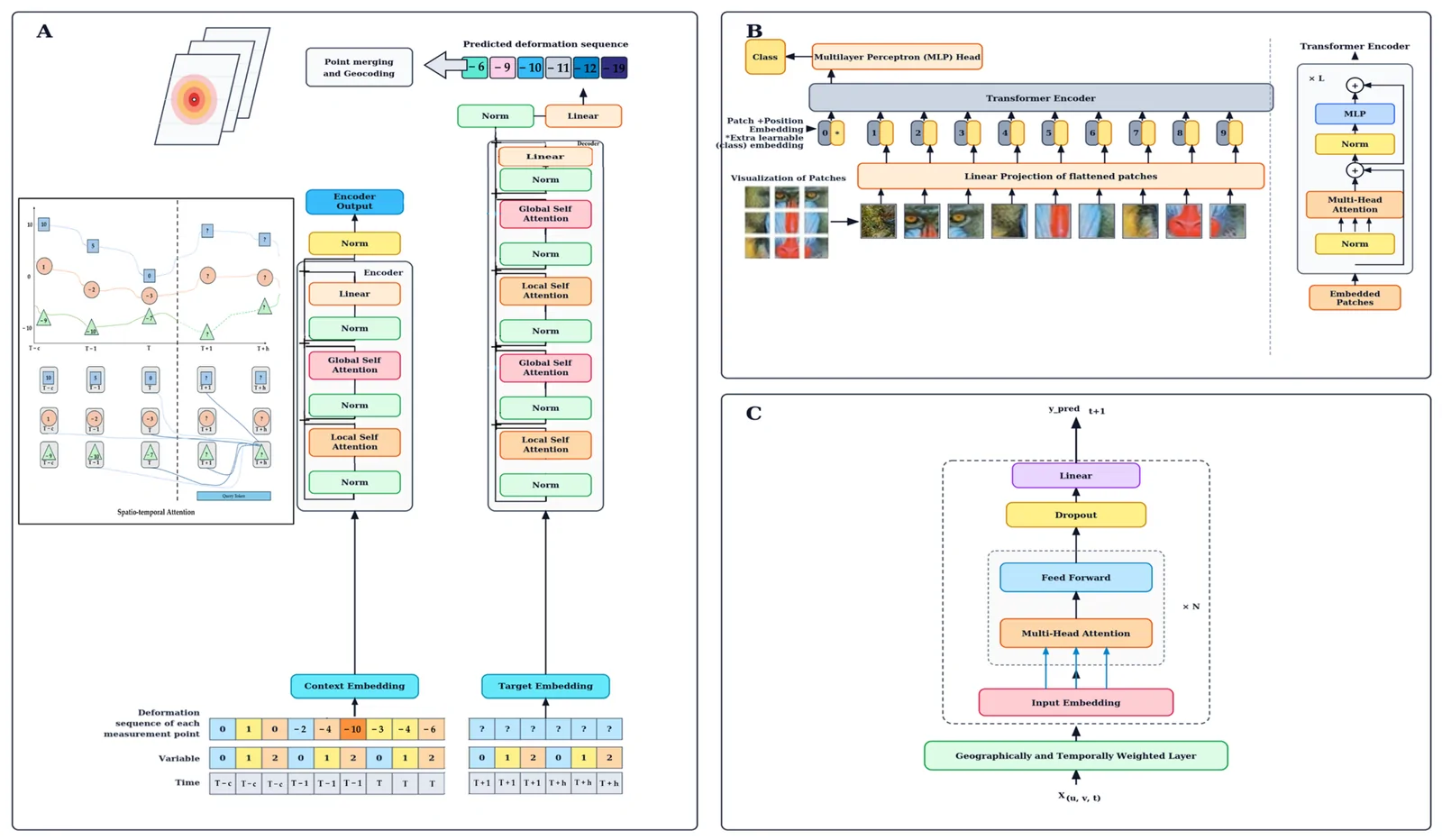
Original comparative schematics of three Transformer-based architectures: (**A**) Spacetimeformer architecture; (**B**) Vision Transformer architecture (* indicates the extra learnable class embedding); and (**C**) Geo-Temporal Transformer architecture - each illustrated based on the concepts described in Wang et al. [113], Kameswari et al. [131], and Zhang et al. [27], respectively.

The specialized architectural topologies developed to leverage multi-head self-attention paths for large-scale spatiotemporal geohazard modeling are categorized and presented in Figure 18. These paradigm variations present the sequence flattening and spatiotemporal attention configurations of the Spacetimeformer pipeline (Figure 18A), the patch-wise linear projection framework of the supervised Vision Transformer model (Figure 18B), and the coupled Geographically and Temporally Weighted encoding mechanics unique to the GTformer network (Figure 18C).

### 5.5. Optimization Strategies and Transfer Learning

There is also substantial progress in model tuning and hyperparameter optimization using metaheuristic algorithms. Yu [132] has used Bayesian Optimization (BO) [133,134] as the main hyperparameter tuning method for both Prophet and Encoder-Decoder deep learning models. Wang et al. [108] used the Snake Optimizer (SO) [135] as their main optimization approach to deal with hyperparameter tuning when pertaining to deep neural networks. SO is a global optimization metaheuristic algorithm that mimics the foraging behavior of snakes in order to find the most competent combination of parameters.

#### 5.5.1. Optimization Algorithms

Meta-heuristic optimization algorithms have been increasingly considered a vital element in the field of subsidence prediction models, used in finding optimal hyperparameters within a given space [134,136]. **Particle Swarm Optimization** is a prominent algorithm that makes use of swarm behavior to discover hyperparameter settings and arrive at optimal model configurations [24,137]. Improved PSO variants that include adaptive inertial weight, dynamic neighborhood topology, and hybrid searches have been found effective in optimizing neural networks [138,139,140].

##### Bayesian Optimization (BO)

**Bayesian optimization (BO)** is a stochastic technique based on probability theory, which helps find the optimal values for hyperparameters through the construction of Gaussian process surrogates modeling the connection between hyperparameters and the performance of the model [133,134]. BO has been implemented alongside LSTM-based predictions of land subsidence, resulting in significantly enhanced performance of the model and superior accuracy and generalization when compared to the models tuned by manual optimization methods [20,132].

##### Snake Optimization (SO)

The **Snake optimization (SO)** [135] initializes a uniform random population, and the solutions are classified by fitness, restricting inefficient ones through competition behavior. The population is split into male and female groups, who interact according to how much they attract each other while solving the problem. The approach seeks solution positions by alternating between exploration and exploitation, while iteratively updating them in search of optimal parameter combinations until convergence is reached. The Snake Optimizer optimizes mainly the following four hyper-parameters, i.e., the learning rate, the LSTM hidden units’ number, attention key dimension and regularization parameter. It is an iterative process to find the best parameter combination through the mimicking of snake foraging behavior.

##### Grey Wolf Optimizer

Zeng et al. [37] used **GWO (Grey Wolf Optimizer)** [141], where the optimal hyperparameters of LSTM are automatically discovered, similar to the hunting behavior of wolves in a hierarchical way. GWO adopts a four-level wolf hierarchy in which 30 wolves stand for different combinations of hyperparameters: *α*-wolf (best solution), *β*-wolf (second-best solution), *δ*-wolf (third-best solution) and 27 *ω*-wolves (left solutions). Each wolf’s position encodes three LSTM hyperparameters: hidden units (0–200), max epochs (0–200), and learning rate (0.001–0.5).

(a) **Optimization process**
  i. Each wolf builds and trains an LSTM model with its hyperparameters
  ii. Performance is evaluated on the test set using RMSE as fitness
  iii. Wolves update positions by moving toward the top three leaders using Equation (11):
    (11) $$x\left(t+1\right)=\frac{\left(\vec{X_{\alpha }}-\vec{A_{1}}{\vec{D}}_{\alpha }\right)+\left(\vec{X_{\beta }}-\vec{A_{2}}{\vec{D}}_{\beta }\right)+\left(\vec{X_{\delta }}-\vec{A_{3}}{\vec{D}}_{\delta }\right)}{3}$$
  iv. Convergence coefficient vector is computed using Equation (12), where the control parameter $\vec{a}\;$ decreases from 2 to 0, transitioning from global exploration to local exploitation
    (12) $$\vec{A}=2\vec{a}\cdot \vec{r}-\vec{a}$$
(b) **Key components:**
  i. *D* = ∣*C* ⋅ *X_P_*(*t*) − *X*(*t*)∣ i.e., the distance of a wolf from each leader, and
  ii. *C* = 2*r* is a stochastic swing factor [141]
  iii. Position update: Wolves average movements toward three leaders, not just one
  iv. Adaptive search: Weight factor *a* linearly decreases, balancing exploration and exploitation.

The Mathematical formulations presented in Equations (11) and (12) are adapted from Zeng et al. [37].

**Figure 19:**
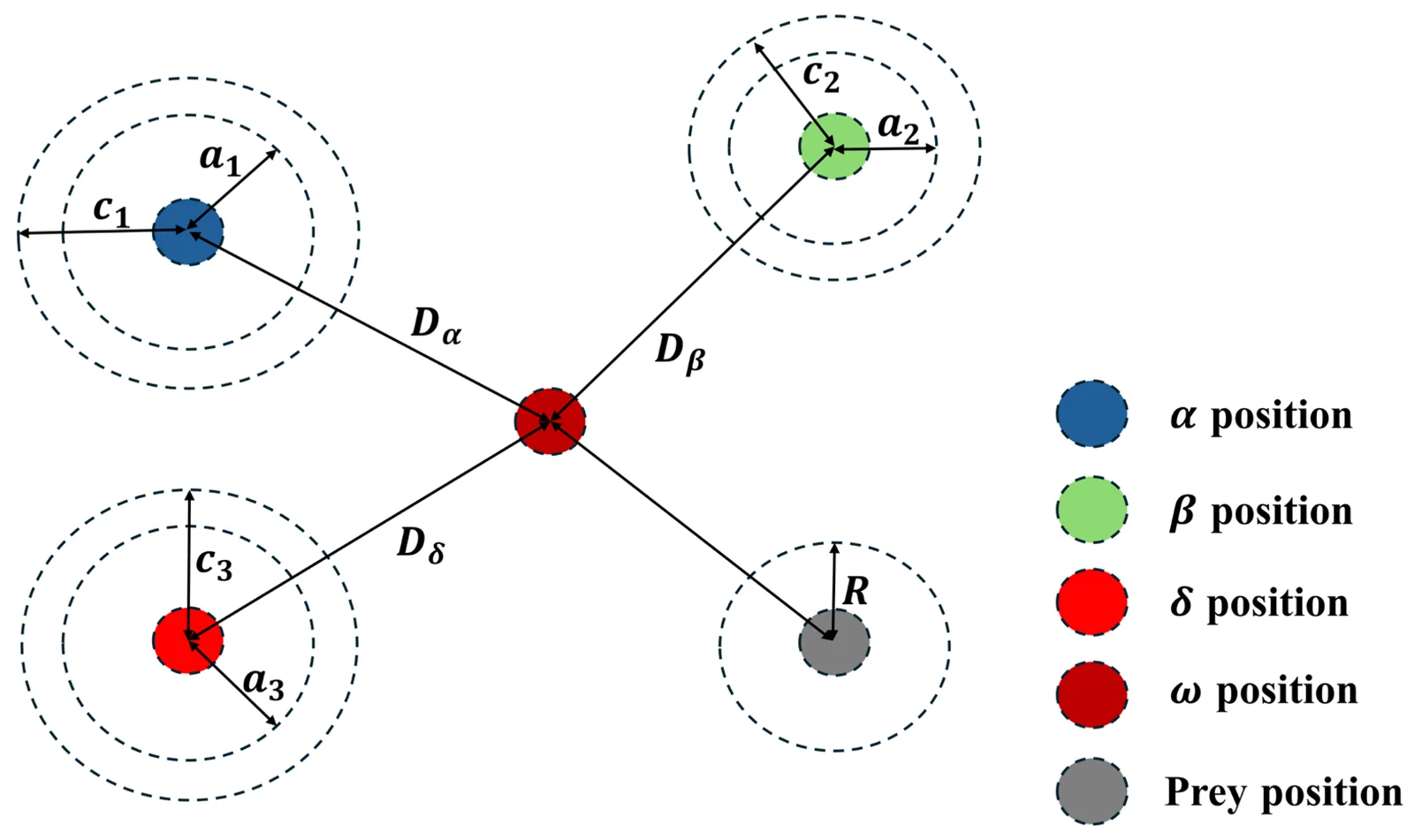
Position update mechanism in the Grey Wolf Optimizer (GWO), showing how a search agent (*ω*) adjusts its position using distance vectors relative to the three dominant solutions (*α*, *β*, *δ*), with the prey representing the global optimum, enabling exploration and exploitation in the search space. Redrawn and modified based on the framework by Zeng et al. [37].

The bio-inspired hyperparameter search matrix follows a strict multi-tier hierarchy to avoid premature local optima traps, as shown in Figure 19.

##### Beetle Swarm Optimization (BSO)

Zhao et al. [24] used the **BSO algorithm** [142] to optimize the initial weights and thresholds of a BP neural network to overcome its random-dependent initialization and trapping in local minima. BSO is a new metaheuristic algorithm based on the combined foraging behavior mechanism of Beetle Antennae Search (BAS) and the information sharing mechanism of PSO, to achieve a balance between local exploitation and global exploration capacities.

For a beetle swarm in an S-dimensional search space, the formula for updating velocity is given as follows:

(13) $$V_{\mathrm{is}}^{k+1}=\omega V_{\mathrm{is}}^{k}+c_{1}r_{1}\left(P_{\mathrm{is}}^{k}-X_{\mathrm{is}}^{k}\right)+c_{2}r_{2}\left(P_{\mathrm{gs}}^{k}-X_{\mathrm{gs}}^{k}\right)$$

where $V_{\mathrm{is}}^{k}$, and $X_{\mathrm{is}}^{k}$ represent the velocity and position of the *i^th^* beetle at iteration *k* respectively; $P_{\mathrm{is}}^{k}$ stands for the personal best position, $P_{\mathrm{gs}}^{k}$ for global best position whereas *c*_1_ and *c*_2_ represent acceleration coefficient.

The formula for updating the position is given as follows:

(14) $$X_{\mathrm{is}}^{k+1}=X_{\mathrm{is}}^{k}+\lambda V_{\mathrm{is}}^{k}+\left(1-\lambda \right)\xi_{\mathrm{is}}^{k}$$

Here, *X*, *V*, and ξ denote the position, velocity, and beetle search increment vectors, respectively.

To stabilize convergence, the inertia weight *ω* in Equation (13) is decreased linearly from *ω_max_* to *ω_min_* over the K iterations [140,142], favoring exploration early and exploitation late (see also Table 1).

The fitness function is defined in terms of the error between the observed and predicted values. The optimization formulations presented in Equations (13) and (14) are adapted from Wang and Yang [142].

##### Whale Optimization Algorithm

Chen et al. [103] used the **Whale Optimization Algorithm (WOA)** to configure the best hyperparameters of the CNN-LSTM prediction model, thus solving the shortcomings that influenced subjective manual parameter tuning and random initialization. WOA is a population metaheuristic based on the bubble-net feeding strategy of humpback whales that demonstrates an advanced trade-off between global exploration and local exploitation by means of different behavioral phases. In this study, WOA optimizes eight crucial hyperparameters: convolutional filter size, kernel size and stride in CNN layers, time steps for LSTM input sequences, dropout rate, learning rate (LR), number of LSTM layers and the number of neurons in a hidden layer. The optimizations include three different hunting strategies that are chosen at each iteration in a probabilistic way. Three hunting strategies are:

A. Encircling prey (exploitation)
B. Spiral motion (exploitation)
C. Search for prey (exploration)

A comprehensive overview of these metaheuristic optimization algorithms and their respective roles in tuning deep learning hyperparameters is synthesized in Table 1.

#### 5.5.2. Transfer Learning

**Transfer learning** techniques that make use of the knowledge gained from other related tasks and domains to improve subsidence predictions are key developments in overcoming issues related to data shortages [36]. Deep learning models pre-trained using large data sets of images or time series data can be optimized for particular subsidence prediction purposes, minimizing the need for extensive training data [58]. The transfer learning method used in InSAR time-series data analysis can share knowledge among diverse geological environments, mining practices, and geographies [36].

(a) **Transfer Learning Architecture and Mathematical Formulation**

The transfer learning approach has two tasks: the source task (synthetic data) and the target task (real-world data). The source task is five-class classification on generated samples, while the target task corresponds to a six-class (including an “undefined” class) real case, related to the complexity of possible application scenarios.

The mathematical formulation of the transfer learning process, adapted from Yang et al. [36], is presented in Equations (15)–(17).

(15) $$\theta_{s}=\arg\;\underset{\theta }{\min}L\left(Y^{s},f\left(X^{s};\theta_{0}\right),g_{s}\left(\cdot \right)\right)$$

*Z^t^* = *f*(*X^t^*;*θ_s_*) (16)

(17) $$\theta_{t}=\arg\underset{\theta }{\min}L\left(Y^{t},Z^{t},g_{t}\left(\cdot ;W_{t},b_{t}\right)\right)$$

where *f*(·) and *g*(⋅) are the feature extraction and classification layers in the DL model, respectively, and ℒ(·) is the loss function. The aim of training is to find model parameters that minimize the loss function. *θ*_0_, *θ_s_* and *θ_t_* are the initialization parameters for the feature extractor from the source-task fine-tuned model, disjoint-source fine-tuning model, and target-task fine-tuning model, respectively. *Z^t^* denotes the initial feature representation of the target domain samples extracted using the source-domain fine-tuned feature extractor. The classification layer of the source task was replaced and reinitialized for the target task, where *W_t_* and *b_t_* are the classification layer parameters of the final target task, as the source task is five-class simulated data while the target tasks are six-class real data.

(b) **Two-Stage Training Process**
  - Stage 1 (Pretraining): Models (BiLSTM, TCN, Transformer) are trained on 10,000 simulated InSAR sequences representing five canonical deformation patterns with Gaussian noise (σ = 0.83 mm) to match real-world characteristics.
  - Stage 2 (Fine-tuning): Feature extraction layers transfer learned parameters, while the classification layer is replaced to accommodate a six-class real-world taxonomy (adding an “undefined” category), fine-tuned on 3566 real labeled samples.
(c) **Key Findings and Performance**

On real data, the TCN model attained 91.4% accuracy following transfer learning, compared to 83% trained solely on real data. A decreasing marginal utility effect was noted, where accuracy grew from 35% to 70% with limited initial data and then plateaued at about 91% after using around 3060 labeled samples. It alleviates the need for a large amount of labeled data and enhances model transferability across different InSAR datasets.

The method of transfer learning successfully compensates for data shortage conditions and effectively exploits pre-trained models based on well-observed regions, which increases prediction accuracy and generalizability to regions with a short history.

### 5.6. Hybrid Model Architectures and Ensemble Learning

An emerging methodology in deep learning is the use of **hybrid models**, which incorporate multiple model components. This allows the incorporation of complementary abilities from different neural networks, providing superior prediction performance. The hybrid model of SBAS-InSAR along with LSTM-Transformer is a representative example, where the former captures local dependencies, and the latter is responsible for modeling global dependencies in the data, including those that occur in seasonality [143].

#### 5.6.1. Hybrid Architecture

Another significant advance is the attention-augmented **hybrid architecture**, where an attention mechanism is employed along with the CNN-LSTM or CNN-BiLSTM framework to formulate triple attention-based predictive models [33,99]. In the DACLnet (Dual-Attention-Mechanism CNN-LSTM Network) architecture, spatial attention is applied in the CNN structure to highlight useful spatial areas, while temporal attention is incorporated into LSTM networks to highlight crucial historical instances, resulting in state-of-the-art accuracy for predicting nonlinear InSAR deformations [33]. Likewise, CNN-BiGRU-attention structures employ bidirectional GRU networks combined with attention-boosted CNN feature learning for precise subsidence prediction in mining [99].

#### 5.6.2. Ensemble Learning

**Figure 20:**
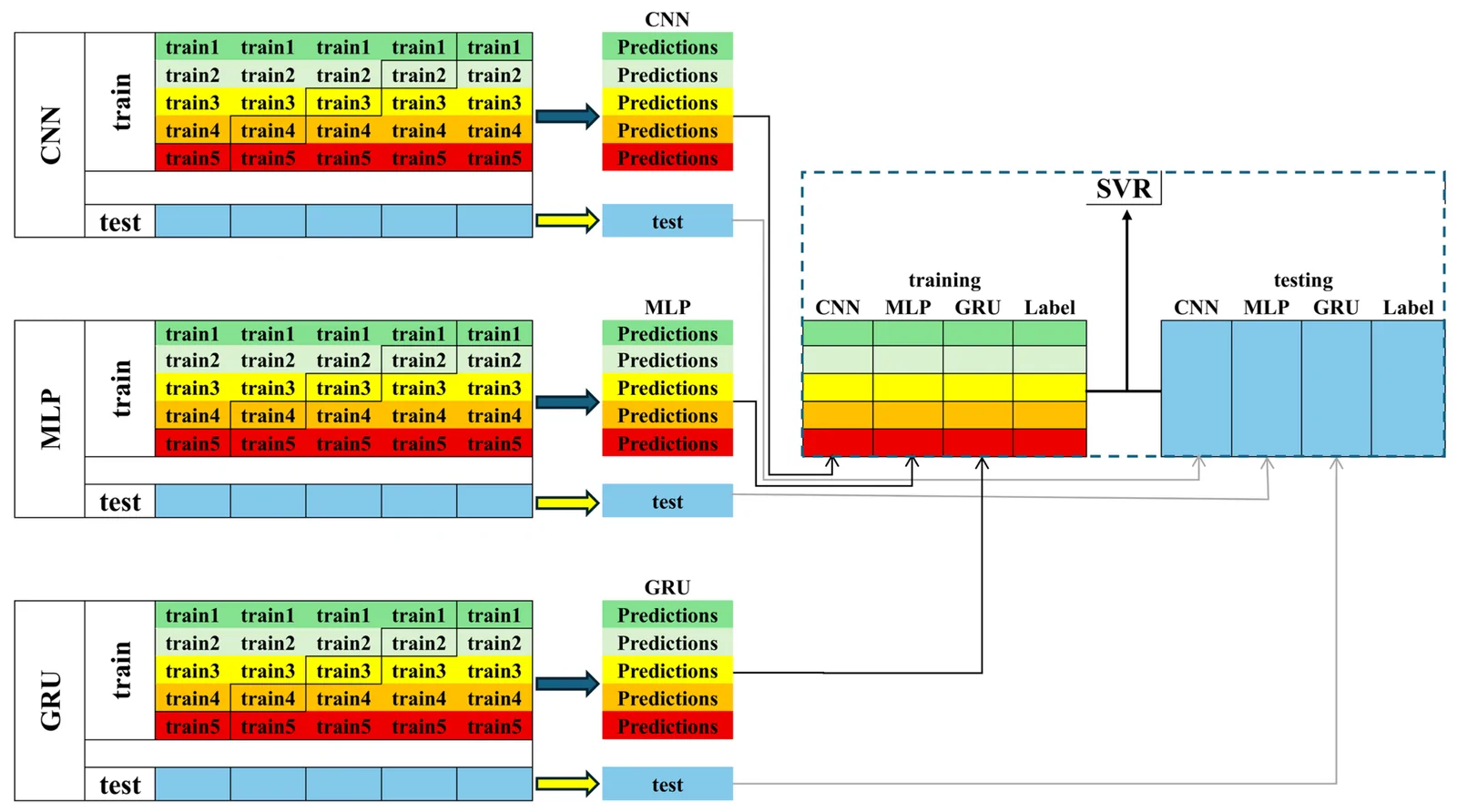
Conceptual illustration of a stacking ensemble learning model structure, based on the approach described in Gao et al. [144]. (Different colour blocks represent the distinct data folds used in 5-fold cross-validation, where green to red blocks denote individual training folds/predictions from base models and blue blocks denote the testing set).

Ensemble learning strategies that combine multiple predictions by means of deep learning models appear to be an effective technique for enhancing predictive performance and estimating uncertainties [34,144]. It has been observed that stacking ensembles utilizing CNNs, LSTMs, GRUs, and Transformers, combined with meta-learning techniques for model aggregation, outperform their individual counterparts due to their ability to exploit varied architectural insights [144]. The stacking ensemble learning methodology is schematically outlined in Figure 20. The ensemble approach also provides natural mechanisms for uncertainty estimation through prediction variance across ensemble members, addressing a critical limitation of single deterministic models.

More attention has been paid to the ensemble model in [79], based on multiple models with weight coefficients using the linear combination method. In the paper, the authors use the Moving Average Method to decompose the total displacement of the monitoring points on the Shengjibao landslide into the trend component and the periodic component. Then, the trend component is predicted using the univariate forecasting model based on the LSTM and the Support Vector Regression (SVR) algorithm, respectively. Finally, the cyclical component is predicted using the MVFT models after independently analyzing the displacement and the triggering factors of the landslide. Then, the total displacement is computed as the sum of the predicted trend component and the periodic component. The ensemble model computes the total prediction as

*E_t_* = *W* ⋅ *L_t_* + (1 − *W*) ⋅ *S_t_* (18)

where *L_t_* and *S_t_* are LSTM and SVR outputs, and *W* (optimal weight) is forecasted by an SVR using factors like triggering inputs, model predictions, and their differences. This linear weighted combination (Equation (18)) reduces single-model instability by assigning higher weights to superior predictions per timestep. This approach decomposes displacement into trend and periodic components, predicts each separately, and combines outputs to outperform single models.

### 5.7. Physics-Informed Deep Learning Approaches

Physics-informed neural networks (PINNs) constitute a recent paradigm wherein physical laws are integrated into the deep learning models via specific loss functions, architectural restrictions, or hybrid modeling techniques [145]. Physical law incorporation can help to decrease the demand for data, promote generalization in cases of unseen data, and ensure that predictions comply with physically sensible subsidence processes.

The utilization of physics-informed ConvLSTM architectures for three-dimensional velocity prediction shows the possibility of applying physical laws in recurrent architecture training for the prediction of geological processes related to subsidence movement [95]. The tensor-train physics-informed ConvLSTM approach involves a tensor decomposition method in order to incorporate spatiotemporal fields in a computationally efficient manner, as well as loss terms that enforce the fulfillment of physical laws. Possible extensions of this approach to mining subsidence include using theories of influence functions, strain compatibility equations, or energy conservation equations.

Hybrid modeling frameworks incorporating both physical simulation via numerical analysis and deep learning techniques constitute yet another path towards incorporating physics principles [13]. In such frameworks, finite element modeling might be used to produce artificial data sets supplementing scarce real-world data, or neural networks can learn corrections to simplified physics-based models or alternatively incorporate both physics-based mechanisms and data-driven features in a modular architecture.

**Figure 21:**
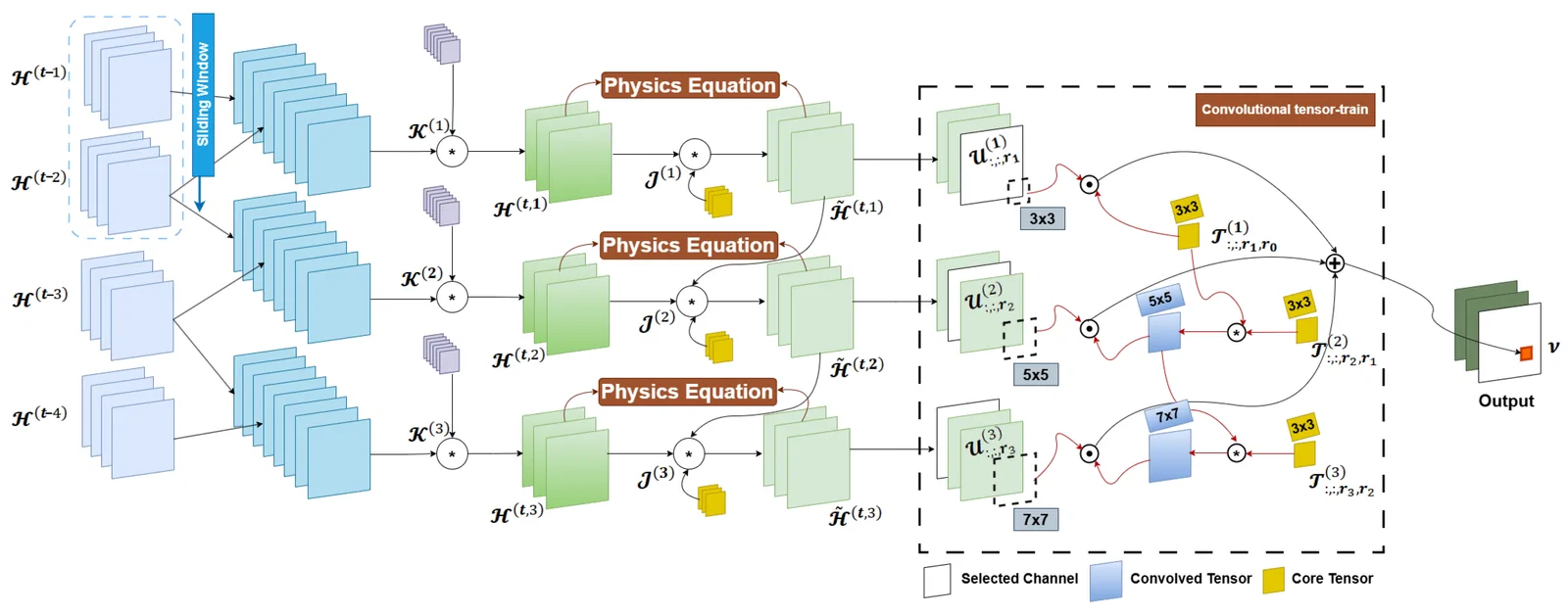
Original schematic of a physics-informed convolutional tensor network architecture, based on the concept described in Huang et al. [95], where * represents the convolution operator across hidden feature maps and tensor-train modules while, ⊙ represents the element-wise multiplication (Hadamard product).

The detailed feature-tensor routing operations, multi-channel convolution steps, and embedded physical equation constraint nodes of the Physics-Informed Convolutional Tensor Network are depicted in Figure 21.

### 5.8. Feature Engineering and Signal Processing Techniques

Well-designed feature engineering and signal processing methods are still indispensable for reliable subsidence prediction. Decomposition methods based on wavelet transform have been extensively used to decompose deformation signals into trend component and periodic component, which can be modeled separately, thus improving predictability. The joining of wavelet decomposition and univariate LSTM for trend forecasting, as well as multivariate CNN–LSTM for periodic displacement modeling, was employed to generate good agreement with InSAR observations.

#### 5.8.1. Variational Mode Decomposition (VMD)

The **Variational Mode Decomposition (VMD)** is suitable for subsidence prediction as it divides the complex non-stationary deformation signal into a group of stable intrinsic mode functions (IMFs), which improve data quality before model training. Wang et al. [108] put forward a VMD-SO-CNN-LSTM-MATT framework in the field of agricultural land subsidence prediction, where VMD is used for signal decomposition and feature extraction. Compared with the time-domain-based EMD, which is susceptible to mode mixing and decomposes modes one by one, VMD finds all variational modes simultaneously in the spectral domain through a convergent iterative optimization procedure. VMD was used in their work to process the cumulative subsidence time series, which reduced the degree of non-stationarity and noise, greatly enhancing the stability of the input for the model. Four was deemed to be the suitable number of decomposition modes, which would have well described the soil subsidence process in a stable manner and assured satisfactory prediction precision. A denoising-related feature pyramid model (DFP) was then created using the decomposed modes, which contains multiple statistical features and frequency-domain features. Feature selection was further conducted to select the most informative features and eliminate irrelevant and redundant variables for model efficiency and prediction accuracy.

#### 5.8.2. Maximal Information Coefficient (MIC)

Zeng et al. [37] made use of the **Maximal Information Coefficient (MIC)** algorithm, i.e., as proposed by Reshef et al. [35], for their feature pre-processing and min-max normalization for its associated data preprocessing in a deep learning approach with their LSTM networks. They also tackle the time-lag dependence that exists in the hydrological time series data of GWL for prediction. Not only the linear functional relation of variables, but also nonlinear functional relations (e.g., exponential, periodic, etc.) can be accomplished through the approach. Here, the MIC method was used to investigate the GWL relationship with trigger factors. MIC models the non-linear relationship between GWL and RWL and/or AR in order to estimate optimal time lags (e.g., 28–35 days for RWL, and 9–13 days for AR). This refinement selects network-relevant triggers, which can decrease redundancy and combine the feature selection of model training to enhance prediction performance. MIC values are between 0 and 1, with strong functional dependence approaching 1, and have an advantage over normal correlations in that they capture both linear and non-linear relationships.

### 5.9. Multivariate Prediction and Integration of Environmental Factors

In the context of current moving-forward models, modern approaches for subsidence forecasting are pursuing multivariate modeling along with deformation time series, considering environmental and human-related factors. These agents typically include groundwater level, precipitation, soil moisture, land use change and temperature variation. The assimilation of GNSS-derived tropospheric products (i.e., IWV and ET) has been shown to largely improve the accuracy for these regions where meteorological stations are scarce, with RMSE values around 3.07 cm/yr compared to what happens when considering only dry troposphere models [21].

The application of the DACLnet model in the Turpan Basin also indicates that it is necessary to incorporate hydrogeological conditions into deep learning models. By directly modeling the relationships between the pumping schedule and surface deformation, the model materially outperformed purely data-driven methods that ignore physical considerations [33].

The taxonomy of DL architectures used in this study, highlighting their core mechanisms and functional strengths, is summarized in Table 2.

**Figure 22:**
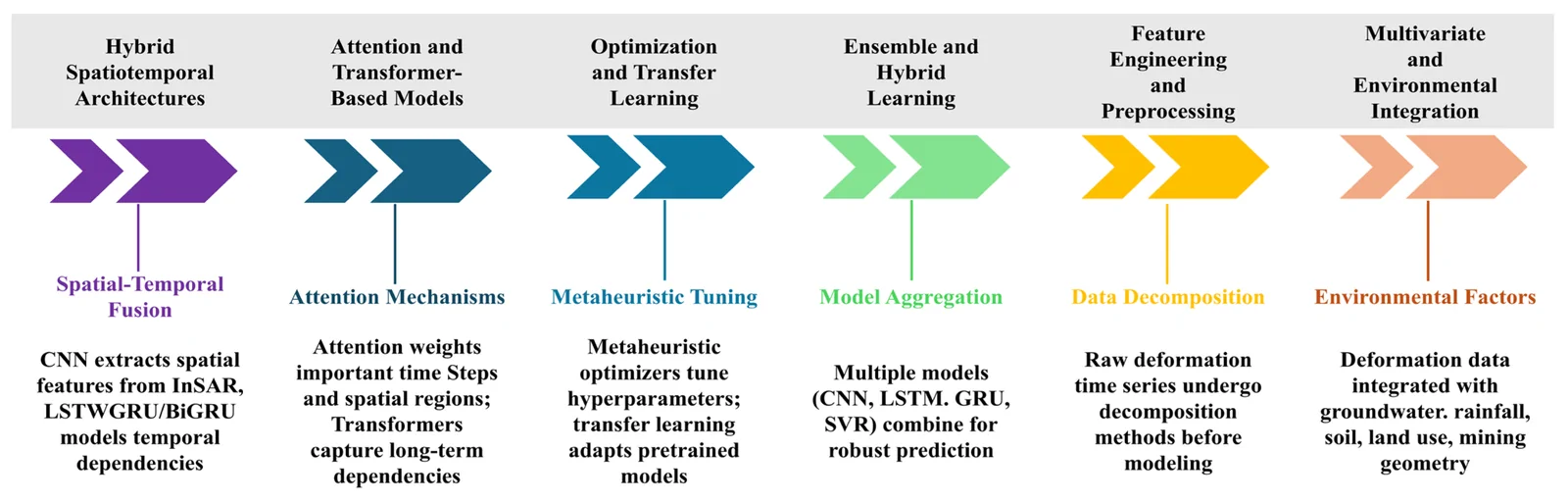
Categorized framework of methodological innovations in deep learning for InSAR ground deformation prediction.

The interconnected evolution, tactical sub-branches, and core signal preprocessing streams of the recent deep learning methodological breakthroughs analyzed in this section are comprehensively synthesized in Figure 22.

## 6. Overview of Deep Learning Applications

This section offers an overview of the application of deep learning models in various geospatial deformation-related applications. As per the PRISMA workflow, 162 studies were ultimately included in this review. Of these, 79 studies apply deep learning models to deformation-related problems (Figure 2a). Seventy-one of them report quantitative prediction results for subsidence or surface deformation and are tabulated in Table 3, Table 4, Table 5 and Table 6 and compared in Table 7; the remaining eight apply deep learning to supporting tasks such as atmospheric delay correction, phase optimization or subsurface property prediction. This indicates that the application of deep learning models in subsidence and surface deformation prediction is becoming increasingly popular. The majority of the application-based studies deal with non-mining and mining subsidence. Landslide prediction and deformation monitoring are the secondary application-based studies. In addition to this, conventional InSAR and GNSS-based monitoring studies were also included in this review. This is because these studies lay the fundamental basis for the application of deep learning-based deformation prediction.

The present section focuses on contextual considerations and characteristics of each domain, specifically, which types of model architectures have been employed in each case study scenario and with respect to which deformation type. Numerical results (RMSE, MAE, R^2^) for each of the studies are not repeated here to avoid duplication. They are presented and compared directly in Table 7 (Section 7) and Table S1 in the Supplementary Material.

It is important to note that these three deformation cases differ physically from one another. As summarized in Table 3b, mining-induced subsidence is caused by engineered geometry and includes the panel and goaf layout, extraction sequence, and overburden failure, resulting in a high-magnitude, rapid process confined within a subsidence bowl. In contrast, groundwater-related urban subsidence is regional in nature and is caused by continuous aquifer compaction due to groundwater extraction; therefore, it features low-magnitude, slow deformations. Lastly, landslide and slope deformation are gravity-induced processes commonly triggered or influenced by rainfall and pore-water pressure, and the spatial consistency in InSAR data is often determined by slope geometry and vegetation cover. This difference explains the inability to directly transfer a well-performing deep learning architecture from one case study scenario to another, which forms the basis for interpreting the results on mining-induced subsidence in Section 6.1.

### 6.1. Mining-Induced Subsidence Applications

Deep learning methods have been increasingly integrated into deformation time series obtained through InSAR technology to monitor and predict surface subsidence caused by mining activities. The most commonly used deep learning methods are LSTM-based models, including traditional LSTM methods for SBAS-InSAR and multi-master InSAR time series, with a maximum absolute error <2 cm for coal field surface subsidence prediction [77], and attention-enhanced LSTM (AT-LSTM) methods that focus on important time segments to improve prediction performance compared to traditional regression methods [65]. Hybrid methods combining decomposition and prediction have also shown improved prediction performance, including VMD-SSA-LSTM methods for prediction of complex multi-component time series for surface subsidence prediction in mining clusters [29], and optimized VMD-multi-model methods that have better prediction performance compared to traditional methods [28], as well as AWC-LSTM methods that combine ARIMA linear decomposition and WOA-optimized CNN-LSTM for nonlinear components, resulting in a RMSE less than 3 mm and an improved prediction accuracy of 28% to 81% compared to traditional methods [103]. More advanced deep learning methods include CNN-BiGRU-Attention methods that use bidirectional and attention mechanisms to improve prediction performance, achieving a sub-millimeter level MAE (approximately 1.27 mm) for coal mine surface subsidence prediction [99], while LSTM-Transformer methods use long-range attention to improve prediction performance compared to traditional LSTM methods [143], and modified LSTM methods that improve efficiency to 98.6% with a reduced RMS error for multi-year surface subsidence prediction [78]. Spatiotemporal modeling has advanced through ConvLSTM networks for complete deformation prediction instead of pointwise series prediction [94], spatio-temporal GRU models with K-means clustering for spatial partitioning achieving an R^2^ value of ~0.99 [68], and multivariate deep learning models with convolutional attention for projecting subsidence expansion over multiple years [23]. Other models included graph-based MRF-GCN for modeling spatial relationships between monitoring points, achieving lower prediction errors than LSTM-based models [118], and CNN-PhLSTM hybrids for joint feature extraction of deformation maps and temporal patterns for two-year prediction [100]. For detection and classification tasks, CNN-based models have been reported to perform better than classical Hough and circlet transform-based models for subsidence trough detection in interferograms [54], deep transfer learning with ResNet50 achieving correct detection of 18 out of 19 troughs for near-real-time early warning systems [58], Vision Transformers achieving ~94% accuracy for classification using optical images [45], and ICOPS (Improved Combined Scatterers Interferometry with Optimized Point Scatterers) InSAR with CNN-based optimized scatterer selection for enhanced robustness of deformation mapping in open-pit mines [57]. Other applications of InSAR-based deep learning models include InSAR-hybrid deep learning for subsidence funnel characterization [14], ConvLSTM for reconstruction of mining-induced fracture and water-inflow channel evolution [96], BO-Prophet for interpretable trend-seasonality decomposition of mine subsidence [132], and joint InSAR inversion-prediction models for coal mine early warning systems [149].

A structured compilation of recent literature mapping specific deep learning models to their respective mining application domains is detailed in Table 3.

### 6.2. Non-Mining Subsidence and Deformation Applications

Following PRISMA screening, non-mining deep learning studies were retained for their direct methodological relevance. Organized by application, these works span six distinct deformation environments.

i. **Urban Land Subsidence**

The most densely populated cluster is related to city-scale settlements resulting from groundwater exploitation, construction, and loading. Chen et al. [82] used a hybrid approach that incorporates SBAS-InSAR and SSA-optimized CNN-LSTM for surface prediction in urban areas, while Liu and Zhang [22] showed that TCN-GRU is better than RNN, LSTM, GRU, and TCN for land surface prediction in Kunming. Yuan et al. [102] also showed that the hybrid approach that incorporates GTWR and LSTM with attention is better for dealing with spatial non-stationarity for land surface prediction in Zhengzhou, while Wen-Jiang et al. [32] added SHapley Additive exPlanations (SHAP)-based explainability to a CNN-BiLSTM-attention pipeline for urban subsidence mapping. Rachmahyastuti et al. [50] applied a Transformer model to GNSS data to analyze land subsidence in Bandung and Yogyakarta, and Zhang et al. [27] introduced a GeoTemporal Transformer specifically for land subsidence monitoring.

ii. **Transportation and Utility Infrastructure**

Several studies focus on deformation, especially concerning direct safety and maintenance implications. For instance, Xue et al. [107] used PS-InSAR and LSTM for railway subsidence, and Li et al. [20] used a combination of SBAS-InSAR and EnKF-optimized LSTM for airport runway forecasting. Similarly, Jin et al. [150] used multi-temporal InSAR for transmission tower foundation subsidence in a salt lake environment, and Zhao et al. [24] used a BSO-BP neural network for ground deformation around the tower. This implies that deep learning has reached a point where maintenance decisions can be made, not just forecasting.

iii. **Heritage Buildings and Geotechnical Structures**

At finer scales, Ma and Lu [26] used an attention-based LSTM for heritage building settlement, where time weighting was used to isolate significant deformation periods associated with construction and groundwater changes. Hu et al. [112] presented an LE-Transformer for foundation pit deformation, and Wang et al. [60] used a U-Net CNN for geotechnical deformation mapping, showing that spatial models are also applicable at the structure level.

iv. **Groundwater and Agricultural Subsidence**

Hydrogeology-based subsidence creates a separate methodological thread in Wang et al. [108], VMD was utilized before CNN-LSTM-MATT for prediction of subsidence in agricultural land, while in Peng et al. [151], ICA-assisted LSTM was utilized for denoising ground motion extracted from InSAR data before prediction. Liu et al. [152] modeled the subsidence caused by measures of reduced groundwater extraction, and Lu et al. [33] applied DACLnet (CNN-LSTM) for groundwater-induced subsidence studies, while Rahmani et al. [153] proposed an interpretable deep learning method for subsidence hazard mapping, where emphasis was given to explainability for decision-making in land-use planning.

v. **Permafrost and Cryosphere Deformation**

Cold region deformation analysis poses a number of challenges due to the effects of the freeze-thaw cycle and the influence of the active layer caused by climatic changes. Wang et al. [113] proposed a variant of the Spacetimeformer network to simultaneously model the interaction and evolution of retrogressive thaw slumps, while Leng et al. [93] approached the problem of the spatiotemporal evolution of regional deformation fields using ConvLSTM as the model for the image sequences of the deformation field. H. Zhou et al. [111] applied the MT-InSAR model with the LSTM network in the Tuotuo River basin to forecast the heaving and subsidence of the active layer caused by climatic changes.

vi. **General Deformation Detection from SAR/InSAR**

A foundational group deals with signal extraction rather than a particular environment. Anantrasirichai et al. [25] successfully extracted slow volcanic and urban deformation signals from InSAR time series using CNNs; Schwegmann et al. [59] successfully separated subsidence features from complex SAR background noise; and Rouet-Leduc et al. [154] successfully allowed for autonomous millimeter-scale deformation extraction at scale. Azarm et al. [52] successfully filled gaps in the data using CNN-based InSAR subsidence interpolation. These methods underpin all domain-specific applications above, as robust signal extraction is a prerequisite for reliable forecasting.

A Summary of the reviewed deep learning studies on non-mining subsidence and surface deformation applications is presented in Table 4.

### 6.3. Landslide and Slope Deformation Applications

Landslide and slope deformation studies are reviewed here not because they share mining subsidence’s physical driving mechanism, but because several of the architectures developed in this domain-particularly graph-based and hybrid decomposition models such as GC-GRU and graph neural network variants-provide direct methodological precedents for the spatial-coupling and graph-based approaches discussed later as future directions for mining subsidence (Section 9.3) and for the hybrid decomposition methods discussed in Section 9.5. The subsection is therefore included for architectural transferability rather than domain overlap.

Deep learning has been extensively employed in landslide susceptibility mapping as well as slope displacement prediction. In the case of landslide susceptibility mapping, stacked ensemble models combining CNN-MLP, InSAR deformation data, and other machine learning algorithms have demonstrated superior performance in AUC value and risk zoning in urban areas [144], while CNN-MLP, InSAR deformation data, and other machine learning algorithms, optimized using the Bayesian approach, have been shown to outperform traditional spatial classifiers in the use of terrain conditioning factors in infrastructure-related landslide susceptibility mapping [157]. In addition, deep CNN-DNN has been shown to produce high-accuracy provincial-scale landslide susceptibility maps, achieving an AUC value of approximately 0.91, which is superior to other machine learning classifiers including SVM, logistic regression, and decision trees [158], while attention-based CNN has been shown to improve the spatial feature learning ability in the creation of more realistic landslide susceptibility zoning maps [66]. Ensemble classifiers combining heterogeneous features, including spectral, terrain, and texture features, have been shown to improve the accuracy of landslide body delineation [159], while integrated neural networks combining dynamic InSAR velocity features have been demonstrated to improve the accuracy of landslide susceptibility mapping compared to other machine learning classifiers only using static features [160]. In the case of displacement prediction, hybrid models combining LSTM-SVR have been shown to reduce errors in the prediction of reservoir-induced step-like deformation [79], while VMD-based decomposition frameworks, either in the presence or absence of deep learning, have been demonstrated to effectively handle the prediction of non-stationary displacement data, achieving superior performance compared to other machine learning classifiers including raw series, CNN-BiLSTM, attention, and VMD-SSA-optimized CNN-BiLSTM-attention [101,156]. In addition, the use of graph-based deep learning has been shown to improve the accuracy of reservoir-landslide forecasting using GC-GRU networks, which utilize the spatial dependencies of GNSS stations as nodes in the graph [114], while the use of the LandGNN model, which combines the spatial dependencies of InSAR monitoring stations, has been shown to improve the accuracy of the prediction of the displacement signal [117], while the use of the GNN model, which combines InSAR point cloud data, has been demonstrated to improve the accuracy of the spatially coherent displacement signal [119], while the use of the graph deep learning model, which combines multivariate inputs including GNSS, rainfall, groundwater, and soil-moisture inputs achieve lower RMSE and MAPE than SVM, XGBoost, and LSTM for highway landslide monitoring [116].

The reviewed deep learning applications in landslide and slope deformation prediction are consolidated in Table 5.

### 6.4. Other Geoscience and Engineering Deformation Applications

Deep learning techniques have been further developed and applied to other geoscience and engineering deformation issues beyond the ones discussed above. For example, in geophysical fluid dynamics, the Physics-Informed Tensor-Train ConvLSTM (PITT-ConvLSTM) model is proposed to embed physical constraints into the higher-order states of the ConvLSTM model for the 4D Loop Current Velocity forecasting problem, showing improved long-lead predictions beyond one week with fewer parameters [95]. For the problem of ground settlement caused by tunneling, improved PSO-BP hybrid networks have achieved millimeter-level accuracy in predicting the surface settlement of ultra-shallow rectangular pipe-jacking tunnels [137], while D-DNN models have captured the coupled effects of the excavation of twin-tube double-shield tunnels on the longitudinal settlement profile [161], and other deep learning models have been developed to accurately forecast the deformation of primary support in large cross-section tunnels compared to other ML methods [162]. Transfer learning methods using pre-trained features with sequence similarity search methods, such as the dynamic time warping distance, have been developed to perform InSAR deformation pattern recognition with fewer labeled samples [36], while U-Net models trained using random field simulations have been developed as efficient tools to perform probabilistic slope stability evaluation [60]. In hydrology, both LSTM and GRU networks outperform traditional ANN networks in short-term rainfall-runoff predictions [83]. The use of multi-head attention networks, i.e., MHAFFM, reduces the oscillations in predictions made by LSTMs, thus stabilizing multi-hour flood forecasting [106]. The MIC-GWO-LSTM network has been shown to improve dynamic groundwater level predictions, which are critical in landslide forecasting [37]. In dam and structure monitoring, BiGRU networks with sliding window mechanisms have been shown to outperform traditional regression networks in modeling nonlinear time-dependent movements in concrete gravity dams [86]. In addition, deep learning networks have been shown to be effective in atmospheric delay corrections using BiGRUs, thus improving accuracy in time series InSAR deformation measurements in urban areas [87]. CNN networks have been shown to be effective in compact feature representations, thus improving the transferability of features in high-resolution remote sensing image retrieval [61].

Table 6 provides a summary of deep learning applications reviewed in other geoscience and engineering deformation domains.

In general, the corpus created based on the PRISMA approach reveals the dominance of the applications of deep learning in the field of subsidence research, specifically in the use of InSAR-based spatiotemporal modeling, including the use of the LSTM, CNN-LSTM, ConvLSTM, Transformer, and GNN approaches. The most recent works in the literature are focusing on hybridization, optimization, attention, transfer, interpretability, and physics-informed learning approaches, as demonstrated in the most recent research works, including those of Wang et al. [14], Wang et al. [108], Ma and Lu [26], Yang et al. [36], Rahmani et al. [153] and Cui et al. [143], respectively.

## 7. Comparative Performance Analysis

This section provides an in-depth analysis of the performance indicators, innovative architectures, and real-world applications extracted from 71 advanced scientific research papers (see Table S1 in the Supplementary Materials). The major development pattern found in these papers is the transition from conventional statistical approaches and machine learning models (such as SVR, MLP) to hybrid deep learning approaches.

It is important to note that the mentioned performance metrics (RMSE, MAE, and R^2^) have been extracted directly from the original papers rather than under a unified benchmarking control. Due to diversity in geological contexts, data reliability, and testing procedures, the performance indicators provide overall tendencies rather than a direct ranking.

In particular, the use of CNNs to extract spatial features, LSTM or GRU to predict temporal sequences, and Attention Models or Transformers to assign weights to dynamic features has resulted in highly accurate predictions of geohazards, structural deformations, and hydrological systems. For a better illustration of how the proposed approach performs against classic ones, see the comparison of six representative models presented in Table 7 below. The complete results for all 71 studies can be found in Table S1 in the Supplementary Materials.

## 8. Challenges and Limitations

Various deep learning architectures such as CNNs, LSTMs, ConvLSTM, Transformers, and hybrid models have shown promising performance in prediction studies. However, subsidence prediction using deep learning still faces several challenges. The most severe limitation is data sparsity in regions with insufficient monitoring or historical information. Interferometric coherence of InSAR data can be poor due to vegetation and atmospheric delays, although this problem has been mitigated by using the GACOS approach [15]. The lack of a common benchmark across works impedes cross-model comparison and hinders the search for optimal architectures for specific subsidence patterns.

Model transfer is difficult; those trained on specific mines or geological types do not generalize well to new locations. Geomechanics-informed approaches that do not rely on data are difficult to adapt to newer mining geometries. Transfer Learning has potential, but its performance is limited by the fidelity of simulated time series and real deformation behaviors. Current approaches only retrain the classifier and may not effectively leverage source-domain knowledge when complex deformation patterns are present. The ability of the model to perform decreases in real-world conditions with growing forecast lead times, as lags and trend imperfections are observed in long-term forecasts, while interannual variability is not effectively captured.

The underlying causes of this poor cross-site generalization are rarely examined explicitly in the reviewed literature. In practice, they typically include differences in overburden lithology and seam depth between mines, differing SAR acquisition geometries and orbital configurations that alter the interferometric baseline and coherence, inconsistent atmospheric correction quality across study areas, and models learning site-specific noise patterns rather than the underlying deformation mechanism. Addressing these causes requires either domain-adaptation techniques that explicitly account for the factors above, or physically constrained models, as discussed in Section 9.2, that rely less on site-specific statistical patterns.

Overfitting is a challenging issue for deep learning, especially when the training dataset is limited, or the models are designed and trained on localized regions. Hyperparameter optimization methods, such as grid search and Bayesian optimization, are computationally intensive and domain-dependent. It is difficult to interpret the model, since deep neural networks are often “black boxes”. SHAP value analysis is promising for better interpretation [162]. The majority of studies are based on data-driven recognition of deformation patterns from InSAR time series that do not consider geomechanical knowledge, and as such provide sub-optimal interpretability and prediction accuracy. The physics-informed neural networks [95,145] use physical laws but are limited to hydrodynamic modeling, underscoring the need for advances such as simplified physics formulations, geomechanically informed loss functions, and physical constraints on finite-element model outputs.

This review also has methodological limitations that should be acknowledged. A formal risk-of-bias or quality-appraisal tool (e.g., AMSTAR-2 or a comparable instrument) was not applied to individual studies; instead, inclusion was governed by the criteria in Section 2.3 (peer-review status, Q1/Q2 journal ranking, and provision of quantitative performance metrics), which served as an implicit quality filter rather than a formal per-study risk-of-bias assessment. Likewise, no formal assessment of reporting bias (e.g., publication bias or selective outcome reporting across the included studies) was undertaken, consistent with the narrative rather than meta-analytic nature of this synthesis. Finally, the review was restricted to English-language, peer-reviewed sources from Q1/Q2 journals and tier-1 conference proceedings, which may have excluded relevant regional or grey-literature studies on mining-induced subsidence.

## 9. Emerging Trends and Future Research Directions

Despite the remarkable progress achieved by deep learning models in InSAR-based ground subsidence monitoring and prediction, several key limitations remain unresolved. These limitations simultaneously highlight the most promising directions for future investigation:

### 9.1. Interpretability and Explainability of Deep Learning Models

One of the major limitations, which appears in the current literature on the topic, refers to the problem of model interpretability. As far as the problem of land subsidence prediction using deep learning, an important limitation arises: a lack of interpretability of the inner working mechanism. Such a limitation affects the validity of the model in practical usage, as it cannot relate predictions to the phenomenon itself. In order to overcome the problem, there is an increasing tendency to use methods like SHAP (SHapley Additive exPlanations), as can be seen in Ref. [32]. There is a proposal of the MGTWR-CNN-BiLSTM-AM [32] approach enhanced with SHAP. Using explainable AI (XAI) tools leads to an increase in the reliability and validity of the results. Thus, the result becomes actionable for the government, planners, and disaster management authorities. For future research, attention-based models should be used in combination with post hoc explainability like SHAP/LIME in order to provide a physical link between predictions and geophysical processes.

In the mining studies that were examined, interpretability has been used to increase precision rather than to confirm the model’s knowledge: attention modules decreased errors in AT-LSTM [22], FeTLnet [23] and CNN-BiGRU-Attention [99], but normally the learned weights were not matched with the panel extraction schedule; SHAP-based explanation has been utilized only for urban subsidence [32] and hazard mapping [153]. For mining-induced subsidence, explanations are only relevant if the model has process-related inputs, such as the distance from each surface point to the working face, the face advance rate, mining depth, seam thickness extracted, panel width-to-depth ratio and goaf age [16,39]. The explanation then needs to be validated against known subsidence behavior: temporal attention or SHAP importance should be highest when the face is within the influence radius of the point; the spatial saliency of CNN/ConvLSTM models [14,94] should correspond with the goaf outline; and the predicted maximum subsidence should vary directly with the extracted thickness and inversely with the depth. A model that is precise but fails these tests has found a statistical shortcut and is not suitable for early warning [81]. Similar tests are relevant in other models: in groundwater-driven subsidence, the importance of pumping should emerge with a delay consistent with aquitard drainage [33,37]; in landslides, attention should focus on rainfall or reservoir-drawdown events [79]; the 20-day thermal lag in permafrost models reported in [155] offers a direct comparison.

### 9.2. Integration of Physics-Informed Neural Networks (PINNs)

Current data-driven models suffer from the following inherent weaknesses: these models lack geological process constraints and physical mechanisms, which necessitate further optimizations and explorations. One potential possibility for model improvement may include the combination of the empirical physical model and the deep learning frameworks, using the concept of physics-informed neural networks (PINNs). This approach allows achieving model predictability while keeping the data-driven approach to model development and forcing the model to satisfy the empirical laws; hence providing a number of promising prospects for future research. The basic concept of PINNs was developed by Raissi et al. in Ref. [145], but its application within the realm of subsidence modeling is still in its infancy. Moreover, the use of the PINN framework may provide an intermediate constraint for the standard data-driven framework. For the subsidence models, empirical physical equations accounting for the mining-induced deformation can be derived from the geometric parameters and included in the learning process.

Physics-informed learning is potentially most effective in mining-induced subsidence because, in contrast to rainfall or pumping, the driving process is known. Mine plans specify the panel outline, extraction dates, seam thickness m, depth H and dip α; from these, the probability integral method (PIM) calculates the expected final basin (maximum subsidence W_max_ = q·m·cos α, main influence radius r = H/tan β) and the Knothe time function describes its evolution over time [16,17,39]. None of the mining prediction models in Table 3 take advantage of this information as a training constraint: PIM is used for design [17,39] and deep learning for monitoring-based forecasting [14,22,77,99,143], although a subsidence model has already been applied to assist InSAR phase unwrapping in mining areas [97]. A physics-informed model would minimize L = L_data_ + λ·L_PIM_, where L_data_ is the misfit to the InSAR observations, and LPIM penalizes deviation of the predicted basin and its time evolution from the PIM prediction. If q and tan β are also learnable parameters, the network predicts subsidence and at the same time estimates these parameters for the coalfield-an output mine engineers are already familiar with-and because the constraint is based on the mine plan, it can predict subsidence over panels not yet extracted, something none of the autoregressive models in Table 3 are capable of. Two qualifications must be added: PIM is a single-seam, homogeneous-overburden model and so performs poorly beneath thick, unconsolidated cover, multi-seam mining and faults [17], so λ must be a soft constraint; and InSAR measures line-of-sight motion whereas PIM predicts vertical and horizontal components, so data from ascending and descending passes or an explicit projection is necessary. For the other scenarios, the constraint would change to compaction proportional to head decline in groundwater-driven subsidence [33,152], monotonically increasing displacement in landslides [79,156], and settlement that approaches an asymptote under railway or runway loading [20,107].

### 9.3. Spatiotemporal Feature Coupling: Expansion of Graph Neural Networks (GNNs)

The existing GNN-based approaches discussed in Refs. [114,115,116,117,118,119] have faced some significant limitations in the domain. The existing deep learning models generally concentrate on either temporal or spatial features without capturing interactions between them and their dependencies, leading to decreased prediction accuracy. In the landslide-oriented GNN studies [114,116,117,119], the key challenges were sparse sampling points, integrating the global landslide structural information, mining the latent correlations among landslide features, and extracting spatiotemporal characteristics from global historical data. These works prove the importance of spatiotemporal features but are still not capable of capturing spatiotemporal coupling to the full extent; CNN-LSTM is able to model nonlinear deformations but struggles with long-range temporal dependencies. The future research direction should lie in combining GNN frameworks with transformer-based temporal models, which will allow modeling both heterogeneous spatial relations and dynamic temporal dependencies between multiple deformation data sites at once.

In mining, spatial relationships between surface points are dictated by the extraction layout rather than by actual proximity: two points located above the same panel experience deformation together even if they are far apart, while points separated by a barrier pillar are effectively isolated. Of the mining studies in the corpus, only MRF-GCN [118] constructed a graph among the surface monitoring points and obtained an R^2^ = 0.865, lower than the point-wise LSTM studies (R^2^ > 0.95 [14,77,143]); the panels responsible for the deformation were not included in the graph. A mining-specific graph should include two types of nodes: monitoring points (PS/SBAS pixels, GNSS and leveling stations [48,49,50,51]) and elements of each extraction panel carrying attributes such as seam thickness, depth, and extraction date, which are progressively added as the mining face advances, similarly to the time-varying ST-GNN for roof deformation in [115]. A panel node is connected to all surface points that lie within its influence radius (r = H/tan β [16]); the edge weight is initialized to the value of the influence function and then refined during training, allowing the network to represent a potentially complex, multi-source influence function that surpasses the homogeneity assumptions of PIM. Surface points within the same overburden unit are also connected. The graph would then need to be validated by a direct comparison to the point-only graph in [118] on the same dataset. In landslides, the panel nodes are substituted by trigger nodes (such as rain gauges or reservoir levels) which connect with stations located in the same slope unit [114,116]; while in urban subsidence, well nodes take their place and connect with points found within the well’s cone of depression [33].

### 9.4. Transfer Learning and Cross-Domain Generalization

Ref. [36] uses transfer learning for InSAR deformation pattern recognition, and [58] has applied deep transfer learning for subsidence monitoring. However, an important limitation is that most of the current models are region-specific and have been trained on limited datasets, which makes it difficult to generalize. Transfer learning is a relatively recent development that has found successful applications in various fields of artificial intelligence, including deformation prediction. Future research can be done by developing large-scale pre-trained geospatial foundation models that can be fine-tuned for different geological environments, mining operations, and urban areas, achieving high accuracy with limited labeled data.

In the mining studies we reviewed, transfers were only demonstrated for detection or classification. For example, ImageNet features were transferred to interferograms for trough detection [58], synthetic-to-real pre-training was used for pattern recognition (91.4% versus 83% accuracy) [36], and there was a cross-coalfield severity classification with a Vision Transformer [45]. No studies transferred a model that predicts subsidence magnitude or rate, and every predictive model we reviewed (Table 3) was trained and tested at a single site. Differences between coalfields, such as depth, seam thickness, overburden lithology, extraction method, and radar geometry, are known and measurable, so they should be inputs to the model rather than hidden causes of failure. A model that is conditioned on such descriptors learns a family of responses rather than the response of a single site [16,39]. A practical two-step protocol would be (i) pre-training on PIM-simulated InSAR series that span the published parameter ranges with realistic noise, extending the synthetic strategy of [36], and (ii) fine-tuning on the target coalfield with a handful of labeled points [148] (using a leave-one-coalfield-out evaluation and reporting accuracy as a function of the number of target samples). A geospatial foundation model for mining subsidence that is truly transferable would require this kind of multi-coalfield pre-training. Three concrete problems currently stand in the way of this: the limited availability of labeled, multi-site mining subsidence datasets; the computational costs of pre-training large spatiotemporal models; and the geological diversity across coalfields that may elude a single pretrained representation. The concept should be interpreted as an open research question rather than a near-term solution. City-to-city transfer of urban models seems more feasible since the pumping driver is the same for both cities [27,32]; landslide transfer requires that displacement be normalized by trigger intensity [79,156]; mining-to-urban transfer is not expected due to the fundamental differences in temporal signatures.

### 9.5. Hybrid Signal Decomposition and Multi-Source Data Fusion

VMD and wavelet decompositions (Refs. [28,29,30,31]) demonstrated high potential in the analysis of complicated nonstationary deformation signals. Nevertheless, nowadays existing mining subsidence monitoring systems do not offer adequate spatiotemporal resolution, while subsidence prediction models require good-quality data and correct parameter estimations. One of the promising areas in this field is data fusion using InSAR, GNSS (Refs. [48,49,50,51]), geological logging, measurements of the groundwater level, and meteorological factors in one framework. Furthermore, future studies should focus on the development of conditional forecasting methods including additional factors like mining activities and changes in the groundwater level. Moreover, MT-InSAR combined with GRU models could become a new way to improve subsidence prediction.

As far as the mining literature is concerned, decomposition techniques have been used exclusively on the displacement series: methods like Variational Mode Decomposition (VMD), Singular Spectrum Analysis (SSA), and wavelet decompositions [28,29,149], as well as the ARIMA-plus-network separation of AWC-LSTM [103], separate the time series into generic trend and fluctuation components. Yet in the case of mining, those components represent extraction events. The inclusion of auxiliary drivers has been done in urban models [27,32,33], but they have been rarely used in mining models, although mining drivers are the most extensively documented among all studied domains. Three methodological extensions appear to be necessary. First, physics-residual learning: compute the expected deformation due to the mine based on PIM and its function over time (Section 9.2) and ask the network to forecast the residual, i.e., the quasi-stationary difference between the actual and predicted deformation, which is easier to learn, in agreement with the mine plan beyond the training window; this is an alternative to the ARIMA component of [103]. Second, include extraction-related input data-face position and advance rates, seam thickness and depth, goaf age and borehole lithology-as well as ascending and descending InSAR series to decompose vertical from horizontal deformations, and validate the results against GNSS or leveling data [48,49,50,51]. Third, use mining events (start/end of faces and completion dates of panels) instead of frequency for the segmentation and apply causal decomposition (rolling window) to avoid the inclusion of future data in the training process. In other cases, the auxiliary drivers consist of pumping volume and pressure [33,152], rainfall and reservoir levels with lag selection as in [37,79], thawing degree-days [155], and load/construction phases [20,107].

### 9.6. Transformer Architecture and Large-Scale Spatiotemporal Modeling

Transformer-based methods for deformation prediction have been applied in Refs. [23,27,45,50,109,112,113,143], while the Informer architecture proposed in Ref. [111] has proven highly efficient in long-sequence time-series prediction. Though such models have numerous strengths, they are computationally expensive and always require large, labeled training datasets. Data-driven models, especially deep learning models, have proven powerful at predicting the temporal evolution of ground-surface deformation induced by mining activity. Nevertheless, prediction results can vary with the forecasting horizon and region under consideration, due to local irregularities and the evaluation criteria used. For future work, it would be interesting to develop lightweight Transformer architectures, e.g., Informer and PatchTST, for working with sparse InSAR time series data. Besides, it would be interesting to introduce multi-scale spatiotemporal attention models that can learn both temporal and spatial characteristics of the processes.

The two mining Transformer studies in Table 3, FeTLnet [23] and LSTM-Transformer [143], motivate self-attention by long-range dependencies, yet InSAR series in an individual coalfield are short (typically tens of acquisitions per extraction campaign), so the real constraint is overfitting rather than computational cost, and the dominant driver-extraction-is external and known. Three adaptations follow. First, treat the extraction schedule as a known future input: future face positions are fixed by the mine plan, so architectures that accept known future covariates, such as the Temporal Fusion Transformer applied to GNSS subsidence in [50], turn the task from extrapolation into conditional forecasting (“what is the subsidence if the face advances 150 m next month?”), an advantage unavailable for rainfall or pumping. Second, restrict spatial attention to the influence radius r = H/tan β [16] instead of the whole scene, which imposes physics, reduces parameters and prevents spurious correlations with unrelated ground. Third, prefer lightweight variants (Informer [111], PatchTST) with synthetic pre-training (Section 9.4). Forecast horizons should be defined in terms of face advance, and Transformer, LSTM, ConvLSTM and GNN baselines should be compared on identical InSAR epochs, inputs and splits-a comparison still missing in most studies. In urban subsidence, seasonal pumping plans can serve as partially known future inputs; for landslides, rainfall is unknown, and probabilistic ensembles are required; for permafrost, climate scenarios play this role [113].

### 9.7. Development of ConvLSTM and 3D Spatiotemporal Architectures

ConvLSTM-based spatiotemporal forecasting methods have been proposed in references [92,93,94,95,96]. Nonetheless, a major drawback is that conventional AI algorithms are constrained in their feature selection and in modeling temporal dependencies, preventing them from fully leveraging the spatiotemporal nature of subsidence data [99]. For future studies, hybrid models combining 3D ConvLSTM and Graph Attention Networks can be designed in order to capture both pixel-wise spatiotemporal deformation information and multi-point temporal relationships. Moreover, improving deep neural network-based techniques for atmospheric delay correction (Refs. [87,89,90,91]) remains a critical open issue due to atmospheric noise in InSAR deformation measurements.

The ConvLSTM works in mining [94,96] consider the deformation raster as a video-like image sequence: [94] takes the InSAR displacement maps as the only input and [96] models overburden fracturing, but neither links extraction, overburden state and surface response, and both tend to smooth basin edges. Two improvements follow. One is multi-channel source coupling: the input tensor should include, in addition to the cumulative displacement, a graded goaf mask that indicates the area and thickness of the goaf extracted at each epoch, the overburden thickness and lithology proportion obtained from boreholes, and the DEM, so that the convolutional kernels can learn the transfer function from underground extraction to the surface basin; physics-guided losses from Section 9.2 applied on the grid would sharpen basin boundaries, and a grid-graph hybrid model (ConvLSTM for dense grids, graph attention for sparse PS points, Section 9.3) would handle irregular sampling. The other is true three-dimensional forecasting: by ingesting the ascending and descending tracks with GNSS control [48,49,50,51], the model should predict vertical subsidence and horizontal displacement, from which tilt, curvature, and horizontal strain-the quantities that actually damage buildings, railways, and pipelines [16,39]-are derived; single-geometry line-of-sight forecasts cannot provide these. Since many coalfields lie in areas of complex topography where tropospheric delay correlates with elevation, the deep-learning atmospheric correction methods developed for city clusters [87,89,90,91] should be tested in mining terrain before being placed at the front of such processing pipelines [46]. In open-pit mining [57] and landslides, a DEM-change channel captures material removal or sliding; in urban areas, the thickness of the compressible clay layer serves as a static channel [32].

**Figure 23:**
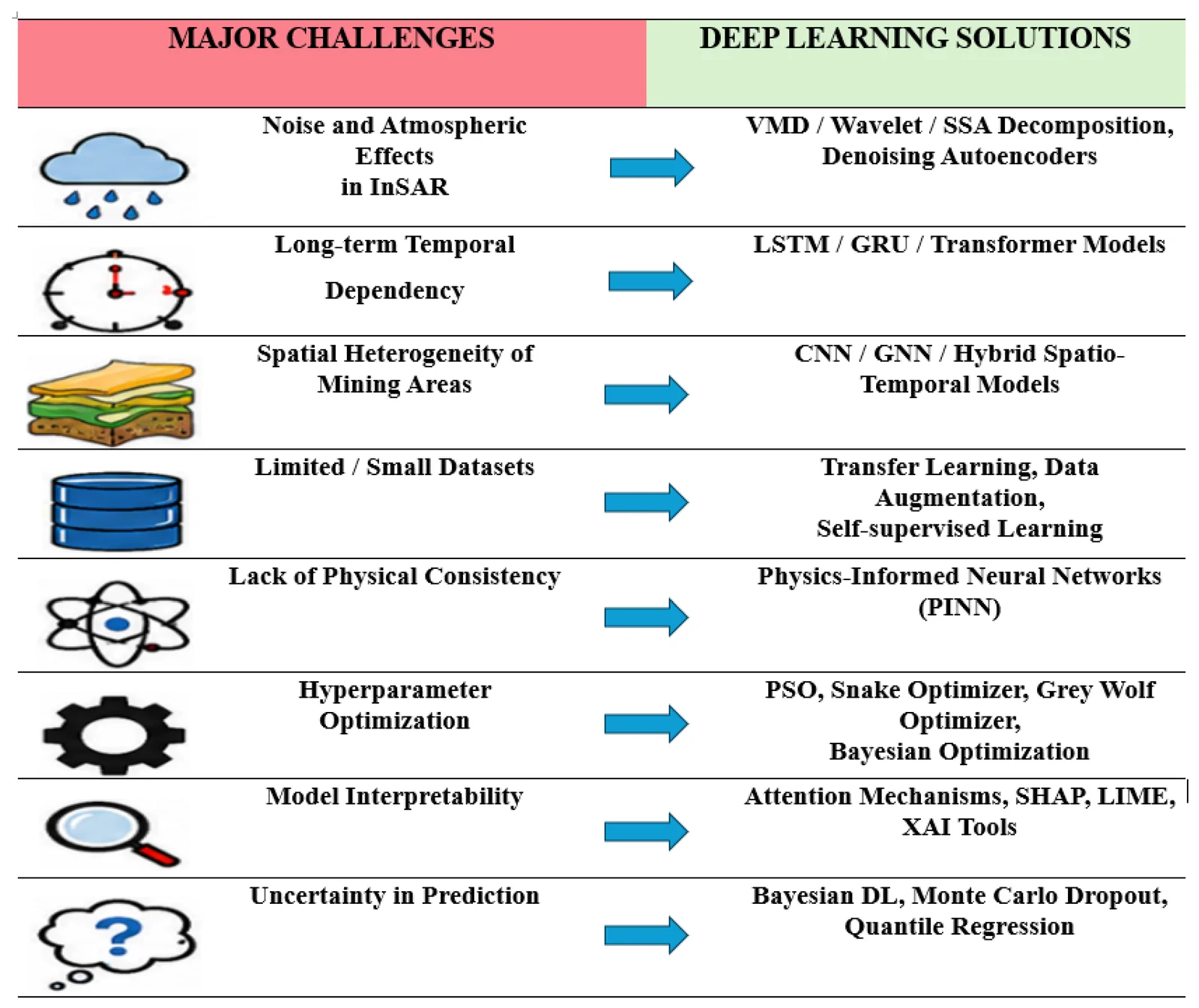
Major challenges in deep learning-based subsidence prediction and their corresponding solutions.

This section discusses the major challenges and limitations that deep learning models suffer from. To overcome these, some future research directions have been suggested. A summary of the major challenges and corresponding deep learning solutions for subsidence is presented in Figure 23.

## 10. Conclusions

This systematic review shows that the analysis of mining-induced subsidence has experienced a significant paradigm shift from Traditional empirical models and physical modeling (Such as the probability integral method and finite element analysis) to advanced, data-driven DL architectures. In essence, the current development of this area is driven by the seamless integration of time-series satellite and GNSS datasets to form an integrated database of high-resolution, millimeter-level displacement measurements. Our findings demonstrate a consistent trend towards a hybrid approach within the literature on spatiotemporal analysis. Specifically, CNNs have emerged as the standard for spatial feature extraction and subsidence trough identification. On the other hand, LSTM networks and their variations (e.g., BiGRU) have been extensively used to forecast the settlement process. Therefore, the state-of-the-art research has largely converged into hybrid spatiotemporal CNN-LSTM models (e.g., ConvLSTM, CNN-PhLSTM). The predictive accuracy of these models is achieved using innovative signal decomposition methods (e.g., VMD, SSA and ICA) and metaheuristic optimization algorithms (e.g., PSO, Sparrow Search, Grey Wolf and novel snake optimizers). Looking ahead to cutting-edge research, the Transformer framework and self-attention paradigm have enabled a significant leap in evolution via efficient identification of long-range global dependencies in deformations using sequence data without the limitations imposed on conventional sequential RNN architectures. In addition, Graph Neural Networks (GNNs) have demonstrated their remarkable capacity to model continuous, non-Euclidean topological relations associated with regional failures.

In addition to providing an overview of trends in architectures used in this domain, the present review is unique from other reviews of DL architectures in InSAR applications, which mainly concentrate on the issue of urban infrastructure monitoring and only a few mining subsidence cases. Nevertheless, despite all the successes achieved with DL-InSAR techniques, several challenges in relation to the problem of the “black-box” nature of DL models, limited generalization of models to other sites, lack of benchmarks for comparing models, as well as the integration of geomechanical physics in learning frameworks, are still present. All of these challenges, however, are not considered just as an area for future research in general, but rather as a part of a seven-point research roadmap:

1. Physics-informed neural networks;
2. Explanation methods grounded in physical principles using SHAP/LIME;
3. Coupled GNN-Transformer architecture for capturing spatio-temporal relations;
4. Geospatial foundation models with transferability between domains with limited labeled samples;
5. Integration of InSAR/GNSS/water table/mining schedule;
6. Lightweight Transformers for addressing irregularities and sparseness in InSAR time series;
7. 3D ConvLSTM-Graph Attention models hybrid for capturing multiple point correlations both pixel-wise and temporally.

Unlike many calls for future work in the field, this particular roadmap offers a concrete way forward. Overall, all of these approaches would contribute to the development of an autonomous early warning system for mining subsidence.

## Figures and Tables

**Table 1:** Overview of optimization algorithms and hyperparameter tuning roles in advanced deep learning models.

References	Algorithm	Key Features	Hyperparameters Optimized
[24]	BSO (Beetle Swarm Optimization)	Hybrid of Beetle Antennae Search (BAS) and PSO. Balances global exploration (early iterations, high ω) and local exploitation (late iterations, low ω)	BPNN weights and thresholds
[108]	Snake Optimizer (SO)	Adaptive position-update mechanism inspired by snake motion behavior Faster convergence than PSO/GA	LSTM neurons, attention heads, learning rate, regularization
[132]	Bayesian Optimization (BO)	Gaussian process modeling + Acquisition function guide’s search Minimal trials needed	Prophet (seasonality parameters) and LSTM (size, layers, window) hyperparameters
[103]	WOA (Whale Optimization Algorithm)	Nature-inspired metaheuristic based on humpback whale bubble-net hunting behaviour Three hunting strategies: 1. Encircling prey (exploitation) 2. Spiral motion (exploitation) 3. Search for prey (exploration)	CNN-LSTM architecture (layers, neurons, dropout, learning rate) Optimizer: ADAM
[137]	Improved PSO (IPSO)	Adaptive inertia weights (0.9 → 0.4) Particle mutation mechanism	Hidden layer nodes Learning rate Network target loss: 0.0004

**Table 2:** Taxonomy of deep learning architectures highlighting core mechanisms, spatiotemporal data handling, and functional strengths in prediction.

References	Architecture	Core Mechanism	Handles Spatial	Handles Temporal	Key Strength
[2]	CNN	Convolution + Pooling	Yes	No	Spatial feature hierarchy
[67]	RNN	Recurrent connections and hidden states	No	Yes	Sequential memory
[19]	LSTM	Gated memory cells	No	Yes	Long-term sequential memory
[80]	BiLSTM/BiGRU	Bidirectional RNN	No	Yes	Full-context sequence labelling
[146]	GNN	Message passing	Yes (graph)	No	Non-Euclidean graph data
[147]	Convolution-LSTM	CNN encoder + LSTM	Yes	Yes	Spatiotemporal sequences
[109]	Transformer	Self-attention	Yes (ViT)	Yes	Long-range dependencies
[148]	Transfer Learning	Pre-train + Fine-tune	Yes	Yes	Low-data generalisation

**Table 3:** Summary of literature on deep learning applications in mining-induced subsidence. (**a**) a. Horizontal comparison of the 21 core mining-induced subsidence studies, added to directly compare model adaptability across the core literature. (**b**) Mechanistic differences between the three deformation regimes.

References	DL Model	Mining Application	Reported Limitations and Applicability Boundaries
[14,22,23,28,29,77,78,99,100,103,132,143,149]	Univariate LSTM and multivariate CNN-LSTM [149]; AT-LSTM [22]; LSTM [77]; Transformer + Bi-LSTM + CNN-Attention [23]; CNN-LSTM [14]; VMD-SSA-LSTM [29]; BO-Prophet [132]; CNN-BiGRU-Attention [99]; VMD + multi-model [28]; LSTM-Transformer [143]; Improved LSTM [78]; AWC-LSTM [103]; CNN-PhLSTM [100]	Time-series prediction of mining-induced surface subsidence/deformation from InSAR (underground coal/metal mines; e.g., Shigouyi Coalfield [77], Jincheng mining clusters [29], Khetri Copper Belt [78], Shilawusu coal mine [103], Jinchuan [100])	**Recurrent models** (LSTM [77], mLSTM [78], AT-LSTM [22], AWC-LSTM [103], LSTM [149]): no explicit spatial coupling between neighbouring PS/SBAS points; recursive multi-step forecasting accumulates error, so reliable lead time is only a few acquisition epochs; ARIMA/wavelet pre-stages [103,149] must be re-identified for every series; all are single-mine validations with no transfer test. CNN hybrids (CNN-LSTM [14], CNN-PhLSTM [100]): require regularly gridded, coherent deformation maps (PS interpolation introduces artefacts); high parameter count and long training (≈2.6 h in [100]); receptive field fixed by patch size. Attention/Transformer hybrids (CNN-BiGRU-Attention [99], FeTLnet [23], LSTM-Transformer [143]): quadratic self-attention memory cost and extra parameters; data-hungry–overfitting risk when InSAR stacks contain only tens of epochs; gain over plain LSTM shrinks at low-deformation points. Decomposition-based (VMD-SSA†-LSTM [29], SCSSA-VMD-ARIMA-BP [28]): VMD mode number K and penalty factor α are site-specific; decomposing the full series before the train/test split leaks future information (boundary effect); sparrow-search tuning adds cost and local-optima risk; BP/ARIMA sub-models are shallow. BO-Prophet [132]: additive trend + seasonality assumption cannot follow abrupt accelerations when the working face passes beneath a point; BO adds search overhead. Boundary: valid for slow-to-moderate, temporally smooth subsidence in coherent (sparsely vegetated) terrain sampled by ≥30–50 SAR epochs; not valid where deformation exceeds the InSAR phase-gradient limit, in decorrelated zones, or for horizons beyond a few months without retraining.
[68,94,118]	GRU + K-means + Optimization [68]; ConvLSTM [94]; GCN [118]	Spatiotemporal (regional/multi-point) prediction of mining-area surface subsidence	**K-means + GRU + SO** [68]: spatial partitioning assumes internally homogeneous clusters; cluster number and SO hyper-parameters need tuning (search overhead, local-optima risk); still point-wise inside each cluster; 0.9% of samples remain above 8 mm error. **ConvLSTM** [94]: needs gridded displacement maps of fixed extent; memory/computation grow with sequence length and resolution; cannot extrapolate outside the training footprint; smooths sharp basin edges. **MRF-GCN** [118]: requires a predefined graph (distance/geological adjacency)-results sensitive to node/edge definition; R^2^ = 0.865 is below point-wise LSTM studies, i.e., spatial fusion alone does not guarantee accuracy; a static graph cannot follow the advancing working face. **Boundary:** suited to regional, multi-panel subsidence with dense and spatially continuous InSAR coverage; not suited to sparse PS networks (ConvLSTM) or where spatial adjacency is poorly defined (GCN).
[45,54,58]	CNN [54]; Deep transfer learning (CNN) [58]; Transformer/ViT [45]	Automatic detection/identification of mining subsidence (troughs) from SAR interferograms [54,58] and optical satellite imagery [45]	**CNN detector** [54]: needs hundreds–thousands of labelled interferogram samples; recognizes trough-shaped fringes only, so atmospheric/topographic fringes generate false positives; AUC 0.87–0.89; no temporal context. **Transfer learning (ResNet-50)** [58]: accuracy depends on source–target similarity (ImageNet natural images vs. wrapped interferograms); validated on a very small sample (19 troughs, 2 false positives); detection only, no magnitude. **ViT** [45]: requires dense ground-surveyed labels for training; outputs categorical severity classes, not subsidence values; optical imagery limited by cloud/vegetation; recall lower for mild classes; high compute/memory. **Boundary:** screening/early warning in coalfields with distinct trough signatures; not applicable to diffuse low-gradient subsidence or quantitative rate estimation.
[57]	DL algorithm optimization (with improved CSI)	Open-pit mining subsidence monitoring	Improves scatterer selection for **monitoring only**-no forecasting component; depends on stable scatterers, whereas open-pit benches change rapidly and decorrelate; hyper-parameter tuning overhead; the “non-significant point” threshold is site-specific. **Boundary:** monitoring of pit walls/waste dumps with persistent scatterers; not a prediction tool.
[96]	ConvLSTM	Mining water inflow channel prediction (overburden fracturing evolution)	Trained on modeled fracture-image sequences rather than in-situ observations, so validity is bounded by the model’s geological assumptions; moderate reconstruction quality (PSNR 10.32); high memory cost for high-resolution image sequences; no uncertainty quantification. **Boundary:** qualitative prediction of overburden fracture/water-channel evolution for panels with geology similar to the modeled case.
(**a**)			
**References**	**DL Model**	**Architectural Category**	**Cross-Site Applicability Note**
[149]	univariate LSTM/multivariate CNN-LSTM	Time-series decomposition + hybrid recurrent	Site-specific; not tested for cross-site transfer
[22]	AT-LSTM	Attention-augmented recurrent	Site-specific
[77]	LSTM	Baseline recurrent	Site-specific
[23]	Transformer + Bi-LSTM + CNN-Attention	Hybrid attention/recurrent/convolutional	Multi-component; higher complexity
[14]	CNN-LSTM	Hybrid convolutional-recurrent	Site-specific
[68]	GRU + K-means + Optimization	Clustering + optimized recurrent	Requires optimization overhead
[29]	VMD-SSA-LSTM	Decomposition + recurrent	Decomposition parameters site-tuned
[132]	BO-Prophet	Optimized statistical/DL hybrid	Primarily univariate
[99]	CNN-BiGRU-Attention	Hybrid convolutional/recurrent/attention	Higher model complexity
[28]	VMD + multi-model	Decomposition + ensemble	Decomposition parameters site-tuned
[54]	CNN	Convolutional detection	Detection-oriented, not full time-series prediction
[57]	DL optimization (general)	Optimization-driven DL	Architecture not specified in source
[100]	CNN-PhLSTM	Physics-informed hybrid	Requires physical priors
[96]	ConvLSTM	Spatiotemporal convolutional-recurrent	High computational cost
[94]	ConvLSTM	Spatiotemporal convolutional-recurrent	High computational cost
[143]	LSTM-Transformer	Hybrid recurrent/attention	Higher model complexity
[45]	Transformer	Attention-based	Needs large training data
[118]	GCN	Graph-based	Requires defined graph structure
[78]	Improved LSTM	Modified recurrent	Site-specific modification
[103]	AWC-LSTM	Weighted/attention recurrent variant	Site-specific modification
[58]	Transfer learning	Cross-domain adaptation	Depends on source-target domain similarity
(**b**)			
**Aspect**	**Mining-Induced Subsidence**	**Groundwater/Urban Subsidence**	**Landslide/Slope Deformation**
Driver	Goaf/void closure, roof caving, overburden movement following extraction	Aquifer compaction from head decline; building load; consolidation	Gravity-driven shear on a slip surface; rainfall, reservoir level, seismicity
Spatial form	Localized trough/funnel bounded by the working panel; steep gradients; well-defined boundary angles	Regional bowl (km–tens of km); smooth gradients	Elongated body along slope; strongly anisotropic
Temporal signature	Rapid onset tied to face advance, then decay (active → residual); possible post-closure uplift from groundwater rebound [23]	Slow, quasi-linear with seasonal (pumping) cycles; partially recoverable	Step-like accelerations, creep-to-failure; strongly triggered
Dominant magnitude	cm–dm per year, up to >15 cm/yr at open-pit dumps [57]; cumulative up to several dm [14]	mm–cm per year	mm–m; can be catastrophic
Physical prior available	Yes-probability integral/influence-function models [16]; consolidation models [17]	Terzaghi/Biot poroelastic consolidation	Limit-equilibrium, creep laws
InSAR challenge	Phase gradient exceeds unwrapping limits [97]; vegetation decorrelation in rural coalfields [14]	Generally coherent (urban PS); atmospheric delay dominant	Layover/shadow, vegetation, LOS geometry
Implication for DL	Needs non-stationary, panel-aware, extraction-schedule-conditioned models; transferability across coalfields is the open problem	Long-window LSTM/Transformer with hydrological covariates work well	Trigger-conditioned models; graph models on GNSS networks

**Table 4:** Summary of literature on deep learning applications in Non-mining subsidence and deformation applications.

References	DL Model	Application	Reported Limitations and Applicability Boundaries
[27,32,50,82,102,129]	SSA-CNN-LSTM [82]; TCN-GRU [129]; GTWR + LSTM + Attention [102]; MGTWR-CNN-BiLSTM-AM [32]; Transformer [50]; GeoTemporal Transformer [27]	Urban land subsidence/surface deformation prediction (Zhengzhou [102], Shanghai [32], Bandung and Yogyakarta [50])	**SSA-CNN-LSTM** [82]: hyper-parameter search overhead/local optima; depends on soil-temperature (3 depths) and humidity inputs seldom available; accuracy varies between points (r = 0.83–0.93). **TCN-GRU** [129]: point-wise, no spatial coupling; receptive field fixed by dilation depth; validated at only three points. **GTWR-LSTM-AM** [102]: GTWR bandwidth selection; MAPE still ≈20% in low-rate areas; attention adds parameters. **MGTWR-CNN-BiLSTM-AM** [32]: multi-module complexity; needs monthly groundwater-extraction/precipitation and annual land-use/soil layers; SHAP explanation is post hoc. **Transformer/TFT on GNSS** [50]: few stations → no spatial coverage; data-hungry; near-perfect metrics (R^2^ 0.997, RMSE 0.01 mm) indicate very smooth series/possible overfitting and need independent verification. **GTformer** [27]: requires urbanisation covariates (metro distance, POI/road density) absent outside cities; ≈8% of cells still have R^2^ < 0.6; quadratic attention cost. **Boundary:** cities with dense PS/SBAS coverage and accessible hydro-urban covariates; not transferable to rural or mining terrain without retraining and new covariates.
[20,107]	LSTM [107]; LSTM + EnKF [20]	Linear transport infrastructure subsidence (high-speed railway [107], airport runway [20])	**LSTM** [107]: plain LSTM showed a one-cycle lag that had to be corrected with VMD + AdaBoost (extra tuning); PS density drops in vegetated railway sections; point-wise. **LSTM + EnKF** [20]: EnKF assumes Gaussian error statistics; ensemble size vs. computation trade-off; single airport and short series; no spatial coupling. **Boundary:** linear structures with continuous PS (rails, pavements) and slow settlement; not suitable for abrupt failures or PS gaps longer than the model’s context window.
[24,150]	Deep learning (MT-InSAR) [150]; BSO-BP [24]	Transmission tower foundation subsidence	**GWO-LSTM/CNN-LSTM** [150]: salt-lake setting only; cross-site generalisation not verified; GWO search overhead. **BSO-BP** [24]: shallow BP network–limited capacity for long non-stationary sequences; BSO tuning cost; small, conference-level dataset; cross-site not verified. **Boundary:** tower-by-tower trend estimation from sparse records; no spatially continuous forecast.
[26,112]	LSTM + Attention [26]; LE-Transformer [112]	Building/construction-site deformation (heritage building communities [26], foundation pit [112])	**LSTM + Attention** [26]: few PS on small heritage structures → short, noisy series; attention overhead; validated at cluster rather than single-building scale. **LE-Transformer** [112]: efficient additive attention approximates full attention (accuracy trade-off); construction-stage series are short whereas Transformers are data-hungry; correlation 0.92, below LSTM hybrids. **Boundary:** structure-scale monitoring with high-resolution SAR and dense PS; not for medium-resolution wide-area products.
[60]	U-Net CNN	Geotechnical deformation and failure mechanism (geosystems)	Trained on random-field finite-element simulations, so surrogate accuracy is bounded by the FE constitutive assumptions; needs large synthetic training sets; no temporal component; error increases where failure surfaces localize **Boundary:** probabilistic geosystem analysis with a known soil model; not an InSAR data-driven forecaster.
[108]	VMD-SO-CNN-LSTM-MATT	Agricultural land subsidence	VMD mode number (K = 4) and penalty factor chosen empirically; SO search overhead; five-stage pipeline with many hyper-parameters; RMSE 4.38 mm/MAPE 10.5% is higher than urban/mining studies because hilly farmland is decorrelation-prone. **Boundary:** seasonally (irrigation) driven subsidence on farmland; degrades under dense vegetation.
[33,151,152]	DACLnet (CNN-LSTM) [33]; ICA-LSTM [151]; LSTM [152]	Groundwater-extraction-induced land subsidence prediction (Turpan Basin [33], Willcox Basin [151], Choshui Delta, Taiwan [152])	**DACLnet** [33]: requires pumping-schedule/hydrogeological inputs; dual attention adds parameters; single-basin validation; metrics reported on a normalized scale (MAE 0.0015), not comparable in mm. **ICA-LSTM** [151]: number of independent components chosen subjectively; assumes statistically independent sources; RMSE spans 0.88–5.56 mm. **LSTM** [152]: low explanatory power (test R^2^ 0.69, below ANN 0.74); electricity consumption used as a pumping proxy; gap-filled input data. **Boundary:** aquifer systems with groundwater-level/pumping records; without driver data these models degrade to pure autoregression.
[52,74,93]	ConvLSTM [93]; SSA-LSTM [74]; Deep CNN + InSAR [52]	Regional land subsidence prediction/spatiotemporal interpolation	**ConvLSTM** [93]: gridded input; MAE 10.73 mm (larger than point models); smooths local maxima; high compute. **SSA-LSTM** [74]: hyper-parameter search overhead/local optima; point-wise; normalized metrics. **Deep CNN interpolation** [52]: needs paired sparse/dense training data; RMSE 9.0 mm; extrapolation beyond InSAR coverage unverified. **Boundary:** basin-scale mapping where smoothed continuous fields are acceptable; not for site-specific mm-level forecasting.
[153]	CNN + LSTM	Land subsidence hazard/susceptibility mapping	Categorical hazard labels with subjective thresholds; static susceptibility map (no timing); class imbalance; SHAP explanation post hoc; cross-site not verified. **Boundary:** land-use planning zonation; not early warning.
[25,154]	CNN [25]; Convolutional deep autoencoder [154]	Automatic detection/extraction of slow tectonic and volcanic deformation in InSAR (volcanoes [25], fault creep [154])	**CNN** [25]: needs labelled wrapped interferograms; over-wrapping sensitive to noise; atmospheric fringes cause false positives; no temporal context. **Autoencoder** [154]: trained on synthetic signals; cross-site not verified; requires long, dense (6–12-day) stacks and adequate SNR. **Boundary:** detection of slow (mm-cm/yr) sustained signals in large archives; not quantitative forecasting.
[113,155]	Spacetimeformer [113]; LSTM [155]	Permafrost degradation/retrogressive thaw slump deformation	**Spacetimeformer** [113]: high compute; cross-site generalization not verified; needs multi-year stacks to learn freeze-thaw seasonality. **LSTM** [155]: point-wise; RMSE 4.45 mm is large relative to seasonal amplitude; requires air-temperature input (20-day lag). **Boundary:** periodic active-layer signals with multi-year Sentinel-1 coverage; not for abrupt thaw-slump collapse (decorrelation).
[59]	Deep CNN	Subsidence feature discrimination/classification in SAR imagery	Requires labelled data; highly unbalanced dataset (96.6% accuracy dominated by negatives); classification only; no temporal context. **Boundary:** scene-level screening for subsidence-like features

**Table 5:** Summary of literature on deep learning applications in Landslide and slope deformation applications.

References	DL Model	Application	Reported Limitations and Applicability Boundaries
[79,101,114,116,117,119]	LSTM + SVR [79]; (SSA-VMD)-(CNN-BiLSTM-Attention) [101]; GCN + GRU on GNSS [114]; Graph DL on GNSS [116]; Attentive GNN [117]; Spatiotemporal GNN on InSAR [119]; VMD + LSTM/GRU/CNN with cloud-model weighted combination [156]	Landslide displacement prediction (time-series/spatiotemporal, using GNSS or InSAR monitoring data)	**LSTM + SVR** [79]: point-wise; moving-average trend/periodic split; SVR-estimated weights need trigger data (rainfall, reservoir level); step-like jumps remain difficult. **(SSA-VMD)-(CNN-BiLSTM-Attention)** [101]: pre-processing complexity (VMD parameters optimized by sparrow search); attention overhead; RMSE 9.5–12.4 mm reflects high slide dynamics. **Graph models** (GC-GRU-N [114], Graph DL [116], LandGNN [117], GMTC [119]): require a well-defined graph–edge rules (distance thresholds/correlations) strongly affect results; need dense GNSS networks or PS point clouds; a static graph cannot follow evolving slide boundaries. **Boundary:** instrumented (GNSS) or coherent InSAR slopes with multi-year records and known triggers; not for first-time or rapid failures.
[66,157,158,159,160]	Attention-dilated residual CNN [66]; CNN + Bayesian optimization [157]; CNN–DNN [158];Heterogeneous ensemble learning; TD-CNN + Bi-GRU + MSCNN parallel fusion network with InSAR time-series features [160]	Landslide susceptibility mapping/assessment; Landslide detection/inventory mapping [159]	**DADRCNN** [66]: F1 0.767 despite AUC 0.904 → class imbalance; attention/dilation overhead; inventory labels required. **CNN + BO** [157]: search overhead/local optima; AUC 0.863 (moderate); single infrastructure corridor. **CNN-DNN** [158]: cross-site generalisation not verified; static conditioning factors only. Increased cost and reduced interpretability; F1 63.8% and recall 69.9% → ≈30% of slides still missed; needs multi-source features; class imbalance [159]. **TD-CNN + Bi-GRU** [160]: requires InSAR velocity features → limited to coherent slopes (coverage 84.8%); cross-site not verified. **Boundary:** regional static zonation where complete inventories exist; no information on failure timing.
[144]	Stacking ensemble learning + InSAR	Landslide risk evaluation	Increased computational cost; meta-learner reduces interpretability; needs diverse base learners; risk of meta-learner overfitting on small labelled sets; +8% AUC gain is dataset-specific. **Boundary:** urban risk zoning where several trained models and InSAR rates are available.
[160]	VMD + LSTM/GRU/CNN with cloud-model weighted combination [156]	Landslide displacement prediction	**VMD + LSTM/GRU/CNN cloud model** [156]: decomposition parameters; cloud-model weights need calibration; three sub-models → compute cost.

**Table 6:** Summary of literature on deep learning applications in Other geoscience and engineering deformation applications.

References	DL Model	Application	Reported Limitations
[95]	Physics-informed ConvLSTM	Ocean current forecasting	Requires accurate physical priors; can be computationally intensive to train.
[137]	PSO-BP NN	Pipe jacking tunnel settlement	Additional hyperparameter-search overhead; risk of convergence to local optima.
[36]	Transfer learning	InSAR deformation pattern recognition	Performance depends on similarity between source and target domains; not guaranteed to generalize.
[37]	Combined intelligence	Groundwater level prediction	Cross-site generalization not independently verified in source study.
[60]	U-Net	Geosystem deformation prediction	Cross-site generalization not independently verified in source study.
[83]	GRU/LSTM	Short-term runoff prediction	Limited explicit spatial context modeling; can be costly for very long sequences.
[106]	Multi-head attention	Flood forecasting	Added parameter and computational overhead relative to non-attention baselines.
[161]	Deep learning	Tunnelling surface settlement	Cross-site generalization not independently verified in source study.
[162]	Deep learning	Tunnel deformation prediction	Cross-site generalization not independently verified in source study.
[61]	CNN	Remote sensing image retrieval	Requires substantial labeled training data; limited long-range temporal context.
[86]	BiGRU	Dam displacement prediction	Limited explicit spatial context modeling; can be costly for very long sequences.
[87]	Bidirectional gated recurrent unit (BiGRU)	Improving the accuracy of ground deformation measurements by Atmospheric Delay (AD) Correction Signal Decomposition Handling Data Irregularity	Limited explicit spatial context modeling; can be costly for very long sequences.

**Table 7:** Performance Comparison between models (some examples): best conventional statistical and machine learning approaches versus deep learning hybrid models.

References	Proposed Model/Architecture	Key Performance Metrics	Major Innovation/Improvement
[77,94,100]	SVR	Used as comparator, not primary model	Reported as outperformed by the corresponding DL model in each source study
[28,103]	ARIMA/GM	Used as comparator, not primary model	Reported as outperformed by 63–79% error reduction in [103]
[68]	MLR/RNN	MLR R^2^: 0.9254 (as reported in [68])	Reported as outperformed by the hybrid GRU-based model in [68]
[77]	LSTM	MAPE: 1.1%, WIA: 0.999	Reported significantly higher fidelity than SVR baseline
[103]	AWC-LSTM	RMSE: 0.46–1.06 mm, R^2^: 0.999	Reported 63–79% error reduction vs. ARIMA/GM, 51% vs. standard LSTM
[68]	K-means + GRU + Snake Optimization	R^2^: 0.9918, RMSE: 2.35 mm	Reported to significantly outperform MLR, RNN, and SVR baselines

## Data Availability

No new data were created or analyzed in this study. Data sharing is not applicable to this article.

## Supplementary Materials

The following supporting information can be downloaded at: https://www.mdpi.com/article/10.3390/s26196358/s1. Table S1: Performance Comparison Table [14,20,22,23,24,25,26,27,28,29,32,33,36,37,45,50,52,54,57,58,59,60,61,66,68,74,77,78,79,82,83,86,87,93,94,95,96,99,100,101,102,103,106,107,108,112,113,114,117,118,119,124,129,132,137,143,144,149,150,151,152,153,154,155,156,157,158,159,160,161,162]; File S1: PRISMA 2020 Checklist.
