# Supplementary material for: Advances in Deep Learning Models for Ground Deformation Prediction Using InSAR Approach: A Systematic Review with Special Emphasis on Subsidence as a Geo-Environmental Hazard
Source: Sensors (Basel). 2026 Oct 8;26(19):6358. doi: 10.3390/s26196358 (PMC13667577; doi:10.3390/s26196358)
Supplement: Supplementary file 1 [file sensors-26-06358-s001.zip › sensors-4482861-supplementary.pdf]

Table S1. Performance Comparison Table.

References	Proposed Model / Architecture	Key Performance Metrics	Major Innovation / Improvement	Reported Limitations
[149]	Wavelet Transform + CNN-LSTM	RMSE: 3.676 mm	Decomposition improved accuracy over undecomposed predictions (RMSE 4.446 mm).	Added preprocessing complexity; decomposition parameters need careful tuning.
[22]	AT-LSTM (Attention-LSTM)	MAE: 0.71 – 1.52 mm	Attention mechanism eliminated heavy LSTM fluctuations (MAE dropped from 5.36 to 1.52 mm at Point E).	Added parameter and computational overhead relative to non-attention baselines.
[77]	Deep Learning (LSTM)	MAPE: 1.1 %, WIA: 0.999	Demonstrated significantly higher fidelity and near-perfect consistency compared to SVR.	Limited explicit spatial context modeling; can be costly for very long sequences.
[23]	FeTLnet (CNN-Att + Transformer + BiLSTM)	74.8 % RMSE imp., 74.3 % MAE imp.	Multi-module architecture vastly outperformed standard multivariate Bi-LSTM.	High computational/memory cost; needs large training data to avoid overfitting.
[14]	Hybrid CNN-LSTM	R^2: 0.98 – 0.99	Outperformed standalone CNN and LSTM by capturing both spatial features and temporal trends.	Limited explicit spatial context modeling; can be costly for very long sequences.
[68]	K-means + GRU + Snake Opt. (SO)	R^2: 0.9918, RMSE: 2.35 mm	Significantly outperformed MLR (0.9254), RNN, and SVR; mitigated "big misses".	Limited explicit spatial context modeling; can be costly for very long sequences.
[29]	VMD-SSA-LSTM	Test RMSE: 0.586, MAE: 1.84 mm	Sub-millimeter test error demonstrated high generalization without overfitting.	Added preprocessing complexity; decomposition parameters need careful tuning.
[132]	BO-Prophet	MAE: 2.02 mm, RMSE: 2.36 mm, $R^2 > 0.96$	Demonstrated exceptional precision and consistency in tracking spatial-temporal trends.	Additional hyperparameter-search overhead; risk of convergence to local optima.
[99]	CNN-BiGRU-Attention	MAE: 1.27 mm, RMSE: 1.44 mm	Maintained < 5 mm absolute error even in high-subsidence zones.	Added parameter and computational overhead relative to non-attention baselines.
[28]	SCSSA-VMD-ARIMA-BP	RMSE: 0.41 – 0.89 mm	~58 % to 96 % error reduction over	Added preprocessing complexity;

			standalone baseline models.	decomposition parameters need careful tuning.
[54]	CNN (SSD VGG16 vs TinyYOLOv2)	AUC: 0.89 (Base) / 0.87 (Fast)	TinyYOLOv2 achieved >10x faster operational speed with minimal accuracy loss.	Requires substantial labeled training data; limited long-range temporal context.
[57]	CNN (Deep Learning Opt.)	N/A (Filtering methodology)	Successfully filtered "non-significant" points, improving total deformation reliability.	Requires substantial labeled training data; limited long-range temporal context.
[100]	CNN-PhLSTM	MAE: 1.42, SSIM: 0.9949, PSNR: 54.4	Massive jump in image clarity/structure vs SVR; 2h 37min training efficiency.	Limited explicit spatial context modeling; can be costly for very long sequences.
[96]	ConvLSTM	PSNR: 10.32, MSE: 0.093, BCE: 0.327	Produced the sharpest fracturing images, outperforming TCN architectures.	High computational cost for long or high-resolution sequences.
[94]	ConvLSTM	MAE: ~1.8–2.4, $R^2 > 0.99$, SSIM: ~0.98	Successfully integrated spatiotemporal correlation; superior to SVR, MLP, and GM.	High computational cost for long or high-resolution sequences.
[143]	LSTM-Transformer	RMSE: 1.96 mm, MAE: 1.59 mm	Zone-specific R^2 of 0.97–0.99; topped hierarchy over Transformer, CNN-LSTM, and CNN.	High computational/memory cost; needs large training data to avoid overfitting.
[45]	ViT-Base (Vision Transformer)	OA: 94 %, F1: 0.94 (Internal)	Highly robust external generalization across 10 regions (Recall >94 % for severe cases).	High computational/memory cost; needs large training data to avoid overfitting.
[118]	MRF-GCN	R^2: 0.865, MSE: 1.5989	Graph structure captured spatial interactions; 89 % higher R^2 than standard LSTM.	Requires a well-defined graph structure; sensitive to node/edge definition quality.
[78]	mLSTM (Modified LSTM)	Accuracy: 98.57 %, RMSE: 4.22 mm/yr	Achieved ~0.3 mm/yr max error, dramatically improving upon basic RNN and LSTM.	Limited explicit spatial context modeling; can be costly for very long sequences.
[103]	AWC-LSTM	RMSE: 0.46–1.06, R^2: 0.999	Error reductions of 63-79 % vs ARIMA/GM, and 51 % vs standard LSTM.	Limited explicit spatial context modeling; can be costly for very long sequences.
[58]	ResNet-50 (Transfer Learning)	Acc: ~95 %, F1: 0.951	Correctly detected 18 troughs (vs 14/10 in traditional models) with only 2 false positives.	Performance depends on similarity between source and target domains; not guaranteed to generalize.

[82]	SSA-CNN-LSTM	R^2: >0.96, MAPE: 1.93-2.50 %	Multifactorial input (temp/humidity); stable SSA optimization over traditional MLP/BP.	Added preprocessing complexity; decomposition parameters need careful tuning.
[129]	TCN-GRU	MAE: 1.82–2.29, RMSE: 2.48–2.92	Handled high-complexity non-linear fluctuations better than RNN/LSTM/TCN baselines.	Limited explicit spatial context modeling; can be costly for very long sequences.
[102]	GTWR-LSTM-AM	R^2: 0.972, RMSE: 2.403 mm	MAPE reduced from 29.2 % to 20.1 %; highly robust in severe risk zones.	Limited explicit spatial context modeling; can be costly for very long sequences.
[32]	MGCBA (MGTWR-CNN-BiLSTM-AM)	R^2: 0.993, RMSE: 1.86 mm	Multi-scale integration utilizing SHAP to dynamically weight groundwater & soil moisture.	Limited explicit spatial context modeling; can be costly for very long sequences.
[50]	TFT (Temporal Fusion Transformer)	R^2: 0.997, RMSE: 0.010 mm	Vastly outperformed traditional Transformers (R^2 -0.010); actionable predictive insights.	High computational/memory cost; needs large training data to avoid overfitting.
[27]	GTformer	R^2: 0.989, MSE: 0.109 mm	Incorporated urbanization factors; 14.6 % higher correlation than standard LSTM.	Cross-site generalization not independently verified in source study.
[107]	VMD-LSTMAda-LSTMAda	RMSE: 0.82–1.32 mm, R^2: 0.94–0.95	AdaBoost+VMD solved the LSTM "one-cycle lag" issue, enabling safe railway tracking.	Added preprocessing complexity; decomposition parameters need careful tuning.
[20]	EnKF-LSTM (Seq2Seq)	R^2: 0.955–0.979, MAPE: 1.5–1.9 %	12 % accuracy gain; dynamic EnKF backpropagation allowed real-time adaptation.	Limited explicit spatial context modeling; can be costly for very long sequences.
[150]	GWO-LSTM & CNN-LSTM	R^2: 0.923–0.972, RMSE: 0.48–1.41	Decomposed predictions optimized early warnings for critical grid infrastructure.	Limited explicit spatial context modeling; can be costly for very long sequences.
[24]	BSO-BP (Beetle Swarm Opt.)	MSE: 0.130, MAE: 0.244, MAPE: 1.1 %	Adaptive inertia weight prevented local optima; outperformed PSO-BP & standard BP.	Cross-site generalization not independently verified in source study.
[26]	LSTM + Attention	R^2: 0.95, MSE: ~0	Effective focal extraction of dense building clusters despite object similarities.	Added parameters and computational overhead relative to non-attention baselines.

[112]	LE-Transformer	Corr: 0.92, MAPE: 2.5 %	Efficient Additive Attention (EAA) replaced quadratic matrices, boosting computational speed.	High computational/memory cost; needs large training data to avoid overfitting.
[108]	VMD-SO-CNN-LSTM-MATT	RMSE: 4.38 mm, MAPE: 10.5 %	Snake Optimizer (SO) bypassed local optima traps; superior global noise filtering.	Added preprocessing complexity; decomposition parameters need careful tuning.
[151]	ICA-assisted LSTM	RMSE: 0.88–5.56 mm	17-34 % accuracy gains via independent component analysis of seasonal/linear datasets.	Limited explicit spatial context modeling; can be costly for very long sequences.
[152]	LSTM & ANN	R^2: 0.69 (Testing LSTM), 0.74 (ANN)	Reconstructed missing data effectively mapping electricity consumption to ground levels.	Limited explicit spatial context modeling; can be costly for very long sequences.
[153]	Interpretable DL (SHAP/PSO)	N/A (Feature Interpretation)	Moved beyond prediction to interpretability, identifying GDR as the primary trigger.	Additional hyperparameter-search overhead; risk of convergence to local optima.
[33]	DACLnet (CNN-LSTM + Dual-Att)	MAE: 0.0015, RMSE: 0.0055	Staggering 85-92 % error reduction over standard LSTM; highly data efficient.	Limited explicit spatial context modeling; can be costly for very long sequences.
[113]	Spacetimeformer	RMSE: 1.249 mm, R^2: 0.95	Spatiotemporal attention flattened sequences, beating standard LSTMs in freeze-thaw cycles.	Cross-site generalization not independently verified in source study.
[93]	ConvLSTM	MAE: 10.73 mm, SSIM: 0.965	Preserved local geographical laws; reduced high-subsidence error by >21 mm vs LSTM.	High computational cost for long or high-resolution sequences.
[155]	LSTM	RMSE: 4.45 mm, MAE: 3.42 mm	Correlated environmental factors, proving a 20-day lag between temp spikes and deformation.	Limited explicit spatial context modeling; can be costly for very long sequences.
[25]	CNN	AUC: 0.985 – 0.989	Detected exceptionally slow sustained deformation (3.5 cm/year) by overwrapping fringes.	Requires substantial labeled training data; limited long-range temporal context.
[59]	Deep CNN	Accuracy: 96.6 %	Automated false positive discrimination (99.4 %)	Requires substantial labeled training data;

			even on a highly unbalanced dataset.	limited long-range temporal context.
[154]	Deep Autoencoder	SNR ~0.5 %, SSIM > 0.7	Millimeter-scale detection (2 mm); revealed slow earthquakes twice as extensive as thought.	Cross-site generalization not independently verified in source study.
[52]	Hybrid Deep CNN + InSAR	R^2: 0.98, RMSE: 9.00 mm	85 % accuracy improvement over Kriging/IDW; successfully extrapolated sparse radar data.	Requires substantial labeled training data; limited long-range temporal context.
[74]	SSA-LSTM	MAE: 0.0184, RMSE: 0.0207	Automated tuning solved gradient vanishing; residuals consistently distributed near zero.	Added preprocessing complexity; decomposition parameters need careful tuning.
[144]	Stacking Ensemble (SEL)	AUC: +8 % improvement	Accurately mapped five risk tiers, proving correlation with InSAR deformation rates.	Increased computational cost and reduced model interpretability.
[79]	LSTM + SVR Ensemble	MAPE: 0.40 % – 0.73 %	Dynamic weighting leaned into respective model strengths per time step.	Increased computational cost and reduced model interpretability.
[156]	CNN + Bayesian Opt.	AUC: 0.863, Freq Ratio: 5.73	Piece-wise decay learning mapping risk for 1,658 transmission tower foundations.	Requires substantial labeled training data; limited long-range temporal context.
[101]	SSA-VMD-CNN-BiLSTM-Att	R^2: 0.975–0.988, RMSE: 9.5–12.4	Handled stochastic noise gracefully; outperformed static ML and standalone DL methods.	Added preprocessing complexity; decomposition parameters need careful tuning.
[158]	Heterogeneous Ensemble	F1: 63.81 %, Recall: 69.89 %	Merged FCN-8s, U-Net, Attention U-Net; reduced missed alarms from >50 % to 30.11 %.	Increased computational cost and reduced model interpretability.
[159]	TD-CNN + Bi-GRU	AUC: 0.91, Coverage: 84.79 %	Parallel unified extraction of spatial topography and temporal cumulative deformation.	Limited explicit spatial context modeling; can be costly for very long sequences.
[160]	VMD + LSTM/GRU/CNN	R: 0.987–0.990, MAPE: ~0.5 %	"Cloud model" weighting brilliantly captured sudden "step-	Added preprocessing complexity; decomposition

			like" displacement triggers.	parameters need careful tuning.
[114]	GC-GRU-N	MAE: 3.682, RMSE: 4.429	47 % RMSE reduction vs LSTM; top-tier computational training speed (44.88s).	Limited explicit spatial context modeling; can be costly for very long sequences.
[117]	LandGNN (Attentive GNN)	R^2: 0.957–0.985, RMSE: ~0.007	Interpretable attention weights proved that the model focused on geographic neighbor correlations.	Requires a well-defined graph structure; sensitive to node/edge definition quality.
[119]	GMTC (GCN-MHSA-TCN)	R^2: 0.96, MAE: 0.135 mm	Hybrid framework captured both long-range temporal dependencies and abrupt local changes.	Requires a well-defined graph structure; sensitive to node/edge definition quality.
[124]	GTS (Graph for Time Series)	MAE: 1.14 mm, MAPE: 0.25	Effectively predicted full 3D displacement (E-W, N-S, Vertical) over 1-to-12-hour windows.	Requires a well-defined graph structure; sensitive to node/edge definition quality.
[157]	CNN-DNN	AUC: 90.9 %, F1: 85.5 %	Extracted features from spatial patches; 40 % higher AUC than shallow learning models.	Requires substantial labeled training data; limited long-range temporal context.
[66]	DADRCNN	AUC: 0.904, F1: 0.767	Dilated convolutions expanded the receptive field; mitigated gradient vanishing.	Requires substantial labeled training data; limited long-range temporal context.
[95]	PITT-ConvLSTM	SSIM: 0.687 (5 days), MSE: 6.97	Physics-informed constraints extended reliable forecast windows from 2 days to 15 days.	High computational cost for long or high-resolution sequences.
[137]	IPSO-BP (Improved PSO)	R: 0.98, MAE: 1.07–1.81 mm	Navigated sharp geometric terrain changes better than traditional BP (Max error drops).	Additional hyperparameter-search overhead; risk of convergence to local optima.
[36]	Transfer Learning	Acc: 91.4 %, Kappa: 0.897	Transferred knowledge from 10k synthetic datasets to classify 6 complex urban patterns.	Performance depends on similarity between source and target domains; not guaranteed to generalize.
[37]	MIC-GWO-LSTM	R^2: 0.983, RMSE: 0.0457	Filtered redundant data (MIC) identifying specific lags (e.g., 28-35	Limited explicit spatial context modeling; can be costly for very long sequences.

			days for reservoir levels).	
[60]	U-Net	Acc: 92.3–99.6 %, Error ~1 %	Surrogate for Random-Field Finite Elements; mapped full displacement fields 77x faster.	Cross-site generalization not independently verified in source study.
[83]	GRU & LSTM	N/A	Processed sample generation natively without requiring complex time-step optimization.	Limited explicit spatial context modeling; can be costly for very long sequences.
[106]	MHAFFM	NSE: 0.922, KGE: 0.896	Eliminated gating oscillations; 30.6 % RMSE reduction with interpretable peak control.	Cross-site generalization not independently verified in source study.
[161]	D-DNN + SMA (Double-Input)	R^2: >0.85, MAE: -53.2 %	Modeled combined twin-line excavation effects over simplified single-tunnel models.	Cross-site generalization not independently verified in source study.
[162]	FDNN (CNN+LSTM Fusion)	R^2: 0.910, MAE: 0.043	Resisted overfitting issues seen in XGBoost/DTR; synergistic multi-data integration.	Limited explicit spatial context modeling; can be costly for very long sequences.
[61]	LDCNN	ANMRR: 0.019 (RSD)	2.6x fewer parameters than VGG; 30-D features outperformed standard 4096-D features.	Requires substantial labeled training data; limited long-range temporal context.
[86]	BiGRU	R^2: 0.89, RMSE: 1.70 mm	Captured structural hysteresis (time-lag effects) with 30.4 % lower MAE than standard GRU.	Limited explicit spatial context modeling; can be costly for very long sequences.
[87]	pGA_Fi_BiGRU	53.5 – 64.3 % Noise Reduction	Handled data irregularities (missing data) while resolving seasonal atmospheric interference.	Limited explicit spatial context modeling; can be costly for very long sequences.
