# Supplementary material for: Advances in Deep Learning Models for Ground Deformation Prediction Using InSAR Approach: A Systematic Review with Special Emphasis on Subsidence as a Geo-Environmental Hazard
Source: Sensors (Basel). 2026 Oct 8;26(19):6358. doi: 10.3390/s26196358 (PMC13667577; doi:10.3390/s26196358)
Supplement: Supplementary file 1 [file sensors-26-06358-s001.zip › File_S1_PRISMA_2020_Checklist.pdf]

PRISMA 2020 Checklist

Section and Topic	Item #	Checklist item	Location where item is reported
**TITLE**			
Title	1	Identify the report as a systematic review.	Title (p. 1) now reads "...A Systematic Review with Special Emphasis on Subsidence as a Geo-environmental Hazard."
**ABSTRACT**			
Abstract	2	See the PRISMA 2020 for Abstracts checklist.	Abstract (p. 1) states that the PRISMA framework was applied, reports systematic evaluation of 162 studies (21 core + 58 supporting), and summarises key findings/implications in the accompanying Highlights box.
**INTRODUCTION**			
Rationale	3	Describe the rationale for the review in the context of existing knowledge.	Introduction, p. 2–4, particularly the paragraph beginning "With growing interest in DL-InSAR integration, existing systematic reviews primarily focus on urban infrastructure..." (p. 4), which identifies the gap this review addresses.
Objectives	4	Provide an explicit statement of the objective(s) or question(s) the review addresses.	Section 2.1 "Research Objective and Scope" (p. 4): four explicit research questions (RQ1–RQ4) are stated.
**METHODS**			
Eligibility criteria	5	Specify the inclusion and exclusion criteria for the review and how studies were grouped for the syntheses.	Section 2.3 "Inclusion and Exclusion Criteria" (p. 4–5).
Information sources	6	Specify all databases, registers, websites, organisations, reference lists and other sources searched or consulted to identify studies. Specify the date when each source was last searched or consulted.	Section 2.2 "Search Strategy" (p. 4): Scopus, Web of Science, IEEE Xplore, Google Scholar, ScienceDirect, and SpringerLink were searched; search conducted iteratively from

PRISMA 2020 Checklist

Section and Topic	Item #	Checklist item	Location where item is reported
			January 2024 to July 2025. Exact 'last searched' date per individual database is not separately itemised.
Search strategy	7	Present the full search strategies for all databases, registers and websites, including any filters and limits used.	Section 2.2 (p. 4): Boolean keyword groups (Group 1: Remote Sensing; Group 2: Deformation; Group 3: Deep Learning) combined with AND/OR are reported. Full per-database search strings and database-specific filters/limits are not individually reproduced in the main text.
Selection process	8	Specify the methods used to decide whether a study met the inclusion criteria of the review, including how many reviewers screened each record and each report retrieved, whether they worked independently, and if applicable, details of automation tools used in the process.	Section 2.4 "Study Selection Process" (p. 5–6) and Figure 1 describe the two-round screening (title/abstract, then full-text). Number of reviewers and whether screening was performed independently is specified in the statement newly added at the start of Section 2 (p. 4).
Data collection process	9	Specify the methods used to collect data from reports, including how many reviewers collected data from each report, whether they worked independently, any processes for obtaining or confirming data from study investigators, and if applicable, details of automation tools used in the process.	Section 2.5 "Data Extraction and Categorization" (p. 6–7) lists the extracted fields. Number of reviewers and independence of extraction is addressed in the statement newly added at the start of Section 2 (p. 4).
Data items	10a	List and define all outcomes for which data were sought. Specify whether all results that were compatible with each outcome domain in each study were sought (e.g. for all measures, time points, analyses), and if not, the methods used to decide which results to collect.	Section 2.5 (p. 6–7): performance evaluation criteria (RMSE, MAE, R^2, etc.) specified as outcomes sought; results tabulated per study in

Section and Topic	Item #	Checklist item	Location where item is reported
			Table 7 (p. 47–51).
	10b	List and define all other variables for which data were sought (e.g. participant and intervention characteristics, funding sources). Describe any assumptions made about any missing or unclear information.	Section 2.5 (p. 6–7): bibliographic information, InSAR technique, DL technique, decomposition/pre-processing technique, optimisation technique, application domain, and study area are listed as extracted variables.
Study risk of bias assessment	11	Specify the methods used to assess risk of bias in the included studies, including details of the tool(s) used, how many reviewers assessed each study and whether they worked independently, and if applicable, details of automation tools used in the process.	Section 8 (p. 51–52): a formal risk-of-bias/quality-appraisal tool (e.g., AMSTAR-2) was not applied to individual studies; the inclusion criteria in Section 2.3 (peer-review status, Q1/Q2 journal ranking, requirement for quantitative performance metrics) served as an implicit quality filter rather than a formal per-study risk-of-bias assessment.
Effect measures	12	Specify for each outcome the effect measure(s) (e.g. risk ratio, mean difference) used in the synthesis or presentation of results.	Not applicable – this is a narrative/taxonomic review of deep learning architectures rather than a quantitative meta-analysis with a pooled effect measure.
Synthesis methods	13a	Describe the processes used to decide which studies were eligible for each synthesis (e.g. tabulating the study intervention characteristics and comparing against the planned groups for each synthesis (item #5)).	Section 2.6 “Synthesis and Analysis Framework” (p. 7–8): studies grouped into six themes based on primary DL architecture.
	13b	Describe any methods required to prepare the data for presentation or synthesis, such as handling of missing summary statistics, or data conversions.	Sections 2.5–2.6 (p. 6–8): categorisation into DL Theory/Review/Book, DL Model with Application, ML Theory, Optimization-Based, and Monitoring-only categories (Figure 2,

Section and Topic	Item #	Checklist item	Location where item is reported
			p. 7).
	13c	Describe any methods used to tabulate or visually display results of individual studies and syntheses.	Tables 1–7 and Figures 2–21 throughout Sections 4–9 tabulate and visually display results of individual studies and syntheses.
	13d	Describe any methods used to synthesize results and provide a rationale for the choice(s). If meta-analysis was performed, describe the model(s), method(s) to identify the presence and extent of statistical heterogeneity, and software package(s) used.	Section 2.6 (p. 7–8): thematic/comparative narrative synthesis organised by architecture family (Sections 4–5), application domain (Section 6), and performance metrics (Section 7, Table 7). No meta-analysis was performed, so no statistical model, heterogeneity method, or software package applies.
	13e	Describe any methods used to explore possible causes of heterogeneity among study results (e.g. subgroup analysis, meta-regression).	Not applicable – no formal subgroup analysis or meta-regression was performed; studies are instead organised narratively by architecture family and application domain (Sections 4–6).
	13f	Describe any sensitivity analyses conducted to assess robustness of the synthesized results.	Not applicable – no sensitivity analysis was performed, consistent with the narrative (non-meta-analytic) synthesis approach.
Reporting bias assessment	14	Describe any methods used to assess risk of bias due to missing results in a synthesis (arising from reporting biases).	Section 8 (p. 51–52, newly added paragraph): explicitly states that no formal assessment of reporting bias (e.g., publication bias, selective outcome reporting) was undertaken.
Certainty assessment	15	Describe any methods used to assess certainty (or confidence) in the body of evidence for an outcome.	Not applicable – certainty-of-evidence grading (e.g.,

Section and Topic	Item #	Checklist item	Location where item is reported
			GRADE) is not used in this architecture/methodology-focused review.
**RESULTS**			
Study selection	16a	Describe the results of the search and selection process, from the number of records identified in the search to the number of studies included in the review, ideally using a flow diagram.	Figure 1 "PRISMA Flow Diagram" (p. 6): 257 records identified (199 via database search + 58 via other sources), 235 after duplicate removal, 200 assessed at full-text stage, 162 studies included in the review.
	16b	Cite studies that might appear to meet the inclusion criteria, but which were excluded, and explain why they were excluded.	Figure 1 (p. 6) gives aggregate exclusion counts and categories at the full-text stage (unrelated application domain n=13, no deep learning n=9, subsidence relevance n=7, hardware/architecture n=4, insufficient methodological detail n=3, full text not accessible n=3), but does not cite individual excluded studies by name.
Study characteristics	17	Cite each included study and present its characteristics.	Tables 3–6 (p. 39–51) and the reference list [1–162]; study characteristics (DL model, InSAR technique, application domain) are presented per study.
Risk of bias in studies	18	Present assessments of risk of bias for each included study.	Not reported, consistent with item 11 – a per-study risk-of-bias assessment was not performed or presented.
Results of individual studies	19	For all outcomes, present, for each study: (a) summary statistics for each group (where appropriate) and (b) an effect estimate and its precision (e.g. confidence/credible interval), ideally using structured tables or plots.	Table 7 "Performance Comparison Tables" (p. 47–51): for each of the 71

Section and Topic	Item #	Checklist item	Location where item is reported
			compared studies, the proposed model, key performance metrics (RMSE/MAE/R^2/MAPE, etc.), and major innovation are presented.
Results of syntheses	20a	For each synthesis, briefly summarise the characteristics and risk of bias among contributing studies.	Section 7 "Comparative Performance Analysis" (p. 46–51) and Table 2 (taxonomy, p. 38) narratively summarise characteristics across the 71 compared architectures. Risk of bias among contributing studies is not separately summarised per synthesis (see item 18).
	20b	Present results of all statistical syntheses conducted. If meta-analysis was done, present for each the summary estimate and its precision (e.g. confidence/credible interval) and measures of statistical heterogeneity. If comparing groups, describe the direction of the effect.	Not applicable – no meta-analysis/pooled statistical synthesis was performed; Section 7 and Table 7 report each study's own metrics comparatively rather than a pooled summary estimate.
	20c	Present results of all investigations of possible causes of heterogeneity among study results.	Not applicable – no investigation of statistical heterogeneity was conducted (no meta-analysis was performed).
	20d	Present results of all sensitivity analyses conducted to assess the robustness of the synthesized results.	Not applicable – no sensitivity analysis was conducted (no meta-analysis was performed).
Reporting biases	21	Present assessments of risk of bias due to missing results (arising from reporting biases) for each synthesis assessed.	Section 8 (p. 51–52, newly added paragraph): acknowledges that reporting bias due to missing results was not formally assessed for any synthesis.
Certainty of evidence	22	Present assessments of certainty (or confidence) in the body of evidence for each outcome assessed.	Not applicable – see item 15.

Section and Topic	Item #	Checklist item	Location where item is reported
**DISCUSSION**			
Discussion	23a	Provide a general interpretation of the results in the context of other evidence.	Sections 8–10 (p. 51–56), especially the Conclusion (Section 10, p. 54–56), interpret findings in the context of prior urban-infrastructure-focused DL-InSAR reviews (e.g., discussion contrasting with Fontes et al.).
	23b	Discuss any limitations of the evidence included in the review.	Section 8 “Challenges and Limitations” (p. 51–52): discusses data sparsity, poor InSAR coherence from vegetation/atmospheric delay, lack of standardised benchmarks, poor cross-site model transferability, overfitting on localised datasets, and limited model interpretability as limitations of the underlying evidence base.
	23c	Discuss any limitations of the review processes used.	Newly added paragraph at the end of Section 8 (p. 51–52) explicitly discusses limitations of the review process itself: absence of a formal risk-of-bias tool, absence of formal reporting-bias assessment, and restriction to English-language, peer-reviewed, Q1/Q2-ranked sources.
	23d	Discuss implications of the results for practice, policy, and future research.	Section 9 “Emerging Trends and Future Research Directions” (p. 52–54): seven-point roadmap covering explainable AI, physics-informed neural networks, GNN-Transformer

PRISMA 2020 Checklist

Section and Topic	Item #	Checklist item	Location where item is reported
			integration, geospatial foundation models, multi-source data fusion, lightweight Transformers, and 3D ConvLSTM-Graph Attention hybrids.
**OTHER INFORMATION**			
Registration and protocol	24a	Provide registration information for the review, including register name and registration number, or state that the review was not registered.	Newly added statement at the start of Section 2 (p. 4): "This review was not registered in a public registry (e.g., PROSPERO)..."
	24b	Indicate where the review protocol can be accessed, or state that a protocol was not prepared.	Newly added statement at the start of Section 2 (p. 4): "...no separate review protocol was prepared prior to conducting the review."
	24c	Describe and explain any amendments to information provided at registration or in the protocol.	Not applicable, since no protocol/registration exists (see items 24a–24b).
Support	25	Describe sources of financial or non-financial support for the review, and the role of the funders or sponsors in the review.	Funding statement (p. 56): "This research received no external funding."
Competing interests	26	Declare any competing interests of review authors.	Conflicts of Interest statement (p. 56): "The authors declare no conflicts of interest."
Availability of data, code and other materials	27	Report which of the following are publicly available and where they can be found: template data collection forms; data extracted from included studies; data used for all analyses; analytic code; any other materials used in the review.	Data Availability Statement (p. 56): "Data used have been provided by the corresponding author on request." Authors may wish to strengthen this by depositing the 162-study data-extraction table (Section 2.5 fields) in a repository (e.g., OSF or Zenodo) or as Supplementary Material.

PRISMA 2020 Checklist

From: Page MJ, McKenzie JE, Bossuyt PM, Boutron I, Hoffmann TC, Mulrow CD, et al. The PRISMA 2020 statement: an updated guideline for reporting systematic reviews. BMJ 2021;372:n71. doi: 10.1136/bmj.n71. This work is licensed under CC BY 4.0. To view a copy of this license, visit https://creativecommons.org/licenses/by/4.0/
